# Perceived Workload Using Separate (Filtering Facepiece Respirator and Face Shield) and Powered Air-Purifying Respirator and Integrated Lightweight Protective Air-Purifying Respirator: Protocol for an International Multisite Human Factors Randomized Crossover Feasibility Study

**DOI:** 10.2196/36549

**Published:** 2022-12-01

**Authors:** Amy Price, Ying Ling Lin, Anna S Levin, Fabio Tumietto, Rodrigo Almeida, Ana Almeida, Caroline Lopes Ciofi-Silva, Luca Fontana, Naila Oliveira, Nicola Francesco Parisi, Giulia Marcelino Mainardi, Luciana Cordeiro, Marco Roselli, Paul Shepherd, Luana Morelli, Najmeh Mehrabi, Kathleen Price, Whitney Chan, Shrinidhy Srinivas, T Kyle Harrison, May Chu, Maria Clara Padoveze, Larry Chu

**Affiliations:** 1 Stanford Anesthesia Informatics and Media Lab Stanford University School of Medicine Palo Alto, CA United States; 2 World Health Organization Geneva Switzerland; 3 Department of Infectious Diseases Faculdade de Medicina Universidade de São Paulo São Paulo Brazil; 4 Unit of Antimicrobial Stewardship Local Health Authority, City of Bologna Bologna Italy; 5 Federal University of Itajubá Minas Gerais Brazil; 6 School of Nursing University of Campinas (UNICAMP) São Paulo Brazil; 7 School of Nursing University of São Paulo São Paulo Brazil; 8 University of Bologna Bologna Italy; 9 Animation and Media Arts Concentration Academy of Film Hong Kong Baptist University Hong Kong China; 10 College of Health Sciences and Technology St Thomas University Miami, FL United States; 11 Department of Anesthesiology, Perioperative and Pain Medicine Stanford University School of Medicine Stanford, CA United States; 12 Colorado School of Public Health University of Colorado Aurora, CO United States

**Keywords:** N95, SARS-CoV-2, personal protective equipment, human factors simulation, human factors field study, human factors, health care workers, health care, safety, patient care

## Abstract

**Background:**

The design of personal protective equipment (PPE) may affect well-being and clinical work. PPE as an integrated item may improve usability and increase adherence by healthcare professionals. Human factors design and safety may reduce occupational-acquired diseases. As an integrated PPE, a lightweight protective air-purifying respirator (L-PAPR) could be used during health procedures where healthcare professionals are exposed to airborne pathogens. The human factors affecting the implementation of alternative PPE such as L-PAPR have not been thoroughly studied. The population of interest is health care professionals, the intervention is the performance by PPE during tasks across the three PPE types 1.) N95 respirators and face shields, 2.)traditional powered air-purifying respirator(PAPR), and 3.) L-PAPR. The outcomes are user error, communications, safety, and end-user preferences.

**Objective:**

This study will assess whether the L-PAPR improves health care professionals’ comfort in terms of perceived workload and physical and psychological burden during direct patient care when compared with the traditional PAPR or N95 and face shield. This study also aims to evaluate human factors during the comparison of the use of L-PAPR with a combination of N95 respirators plus face shields or the traditional PAPRs.

**Methods:**

This is an interventional randomized crossover quality improvement feasibility study consisting of a 3-site simulation phase with 10 participants per site and subsequent field testing in 2 sites with 30 participants at each site. The 3 types of respiratory PPE will be compared across medical tasks and while donning and doffing. We will evaluate the user’s perceived workload, usability, usage errors, and heart rate. We will conduct semistructured interviews to identify barriers and enablers to implementation across each PPE type over a single continuous wear episode and observe interpersonal communications across conditions and PPE types.

**Results:**

We expect the research may highlight communication challenges and differences in usability and convenience across PPE types along with error frequency during PPE use across PPE types, tasks, and time.

**Conclusions:**

The design of PPE may affect overall well-being and hinder or facilitate clinical work. Combining 2 pieces of PPE into a single integrated item may improve usability and reduce occupational-acquired diseases. The human factors affecting the implementation of an alternative PPE such as L-PAPR or PAPR have not been thoroughly studied.

**International Registered Report Identifier (IRRID):**

PRR1-10.2196/36549

## Introduction

### Background

This study aims to evaluate human factors during the comparison of the use of lightweight protective air-purifying respirator (L-PAPR) with a combination of N95 respirators plus face shields or the traditional powered air-purifying respirators (PAPRs). Health care professionals include medical doctors, nurses, respiratory therapists, physical therapists, and occupational therapists. The rationale for this research is to evaluate human factor–friendly options for hospitals facing a shortage of disposable N95 respirators or other respirators approved by the National Institute for Occupational Safety and Health (NIOSH). We evaluate the use of PAPRs to protect health care professionals against exposure to aerosols containing the coronavirus SARS-CoV-2 during direct patient care. In these situations, the design of personal protective equipment (PPE) may impact overall well-being and the PAPR design may hinder or facilitate clinical work [1]. The use of PAPRs is an alternative to conventional PPE and is currently used for the care of patients with Ebola and SARS-CoV-2 [2,3]. During the tragic 2014-2016 Ebola outbreak in West Africa, in which many health care workers died, health care professionals commonly used 10 or more distinct and disparate pieces of PPE to protect themselves. Simple protocols to correctly and comfortably don or apply PPE (donning) and remove or doff PPE (doffing) can save lives by reducing infection [2,3]. In Ebola, fear led to donning and doffing protocols that were cognitively burdensome and presented a safety risk [4]. The need to integrate PPE is a necessary design challenge to render it simple, safe, and user-friendly [5]. The use of a face shield in PPE combined with respirators can limit the visualization of facial expressions as they are obscured by the respirator [6]. In these situations, the PPE design can impact professional morale and present an obstacle to clinical care. During the COVID-19 pandemic, health professionals reported discomfort after wearing N95 respirators and eye protection (goggles or face shields) for extended shifts [7]. In addition, N95 respirators were in short supply worldwide during the pandemic, which required taking unusual decontamination measures so that they could be reused; however, decontamination does not clean PPE and led to concerns of wearing stained and unclean PPE [7]. The use of reusable PAPRs may offer improved usability, patient interaction leading to more humane clinical care, and reusability and sustainability, limited only by the maximum number of disinfection cycles before filters degrade.

The L-PAPR is an integrated PPE that may confer safety and comfort advantages to workers, which a clinician can don and doff without an assistant, unlike the traditional PAPR where assistance is needed to don and doff safely. The L-PAPR may reduce the respirator supply challenges as it is designed for multiple years of use rather than a single use only and this may in turn reduce solid waste production. Combining 2 pieces of PPE into a single integrated item may improve usability and comfort, increasing the adherence to an adequate use of PPE, and thus minimizing the risk of occupational-acquired diseases. Studying the human factors that can affect the use and implementation of alternative PPE could support the decision-making process when defining PPE usability for health care settings during the COVID-19 pandemic and in ongoing or future infectious disease outbreaks worldwide.

**Figure 1:**
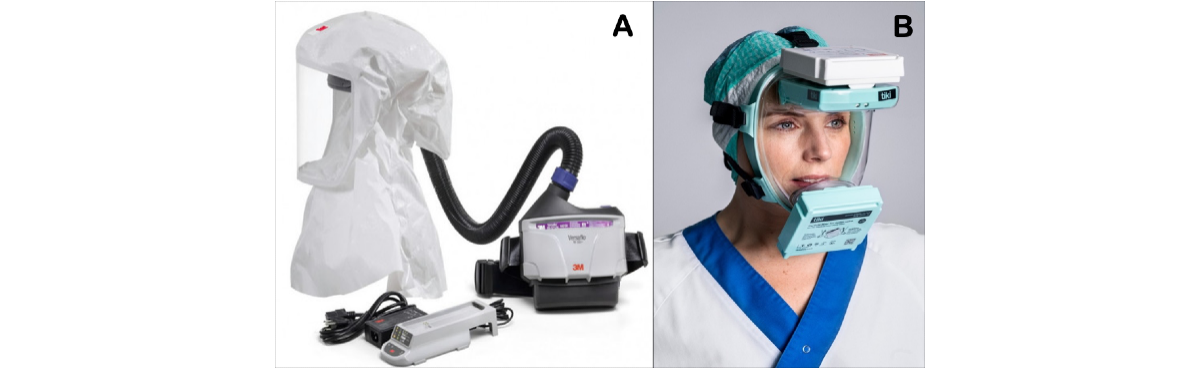
(A) Traditional powered air-purifying respirator (PAPR) and (B) lightweight protective air-purifying respirator (L-PAPR).

The PAPR is an air-purifying respirator that uses a blower to force air through the filter cartridges into the user’s breathing zone (Figure 1B). The process creates a flow of air within the faceplate and hood or helmet, providing a higher assigned protection factor than the reusable elastomeric air-purifying faceplate (half mask) or N95 respirators [8]. A PAPR could be used during health procedures in which the health care professional is exposed to aerosol pathogens to reduce acute respiratory infections [8]. PAPRs can reduce the user-inhaled aerosol concentration to at least one-quarter of that in air, compared with a one-tenth reduction for N95 respirators. This is largely due to its perfect fit to the user’ face, which reduces the inhalation of unfiltered ambient air. Traditional PAPRs have ventilation systems attached and connected to a filtered air supply mechanism. L-PAPRs are battery-operated ventilation systems that allow independent ventilation and air filtration without the encumbrance of the hose. The models we will use for this study are the Versaflo (PAPR A; 3M) [9] and the TIKI Medical Respirator (L-PAPR B; Figure 1A,B) [10].

A previous study demonstrated that PAPRs mitigate the hemodynamic brain effects induced by the prolonged use of N95 respirators during 12- and 24-hour shifts [7]. In another study, health care professionals indicated a preference for PAPRs over N95 respirators in high-risk settings compared with “usual circumstances,” citing comfort, ease of communication, and an enhanced sense of personal safety [3]. A systematic review identified no difference in the contamination of health care professionals by comparing a PAPR with other respiratory protection equipment; additionally, the PAPR was identified as providing greater heat tolerance despite decreased mobility and auditory function [8].

This study aims to evaluate human factors during the comparison of the use of a lightweight PAPR with a combination of N95 respirators plus face shield or traditional PAPR to provide data on usability, design, and implementation for future work. We propose to measure the perceived workload during clinical tasks while using 3 combinations of PPE, first in a simulation project with 3 sites (Stanford, CA, USA; Bologna, Italy; and São Paulo, Brazil), followed by on-site implementation in places of care—COVID-19 wards and the intensive care unit (ICU) at 3 clinical sites (1 in Bologna and 2 in São Paulo, where health care for severe COVID-19 is provided).

### Objectives

The specific objectives of this study are follows:

1. To measure the perceived workload of 3 PPE combinations and to evaluate and compare across objectives 2-4.
2. To measure human errors, equipment failure, and human factors across each of the 3 PPE conditions over a single continuous wear episode.
3. To observe the effects of PPE on interpersonal communications.
4. To evaluate parameters related to stress across PPE conditions and tasks to evaluate the usability perception of health care professionals about the 3 PPE conditions.

## Methods

### Study Design

**Figure 2:**
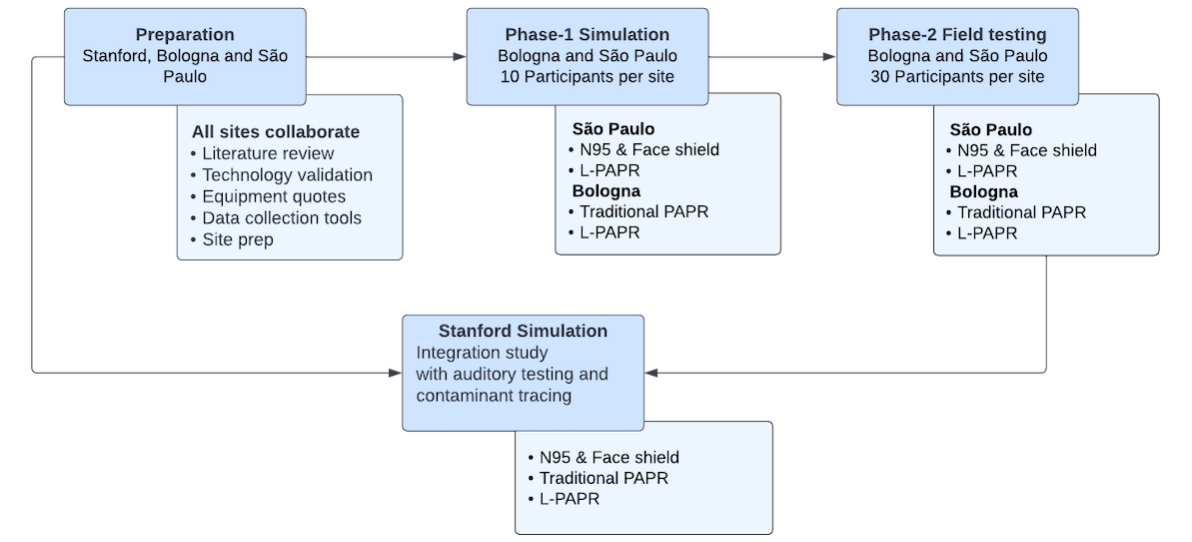
Phases of the study design and sites where the protocol will be put into action. L-PAPR: lightweight protective air-purifying respirator; PAPR: powered air-purifying respirator.

This is an interventional crossover human factors feasibility study combining a simulation study (phase 1) performed in 3 sites (Stanford, Bologna, and São Paulo). Field testing (phase 2) will be performed in 2 sites (Bologna and São Paulo) across 3 ICUs. The results from phase 1 will be integrated with a collaborative study conducted at Stanford (CA, USA). The study design and phases of the research are summarized in Figure 2.

### Inclusion and Exclusion Criteria

See Textbox 1 for details.

A limited number of participants who join the simulation in phase 1 may also volunteer to participate in the field observation phase of the study to provide observations for error similarity between field and simulation sites. Participants who withdraw from the study for any reason after initial consent will be excluded from the analysis.

This research is a 2-phase project consisting of a simulation phase and a field test phase. The results from the simulation phase will be augmented with a parallel study performed at Stanford, where visual and auditory testing will be provided. Stanford will also use part-task trainers as well as high-fidelity complex simulations. During the simulation phase, the Stanford site will test multiple intubation methods. Training with complex and part-task trainer simulations allows testing of the fidelity of part-task trainers. Their lower cost may make them a more viable option in lower-resource settings [11].

This assessment of PPE evaluates human factors in the relationship between PPE (technology) and the clinician (end user). It is hoped that insights from this research might be used to tailor the design of PPE technology for mucosal protection in situations such as admissions, emergency department and ICU care, and in triage centers, where first responders deliver the patient for emergency medical services. According to the Clinical Human Factors Group, “Human factors are organizational, individual, environmental, and job characteristics that influence behavior in ways that can impact safety” [12].

**Inclusion and exclusion criteria.**

**Inclusion criteria**

1. Physicians, residents, nurses, and other health care staff who work the majority of a full-time equivalent in the intensive care unit, the emergency department, or the operating room.
2. Adult health care professional working at the site hospitals who are knowledgeable to complete the simulation exercises.
3. Competent to give informed consent.

**Exclusion criteria**

1. Minors, health care professionals under 18 years of age.
2. Nonhospital site employees.
3. Clinicians without experience of caring for patients with COVID-19.
4. COVID-19 exposure.
5. Positive prestudy screening for COVID-19, including respiratory symptoms or fever.
6. Participants who fail the NIOSH fit test for the L-PAPR.
7. Any individual for whom simulation is contraindicated.

### Sample Size Justification

This is an early feasibility study across 3 continents and multiple languages using a mixed methods approach. In this study, descriptive statistics and mixed methods will be used to ascertain the findings. Recommendations vary from 10-12 per group to 60-75 per group for feasibility size; however, this depends on the study objectives [13]. In this feasibility study, it is not expected that statistical significance will inform study results as it is not powered to report this with accuracy. Formative usability testing and interviews will be carried out with 5-7 individuals as is the standard for this form of research [14]. We selected our usability testing and interview duration to allow for the observation of PAPR use and to observe how well it is suited to COVID-19 intensive care clinical tasks. Saturation of data is our aim, but we chose the number of participants based on studies with similar sample sizes and these studies used interviews lasting 15-20 minutes to reach saturation [14-16]. It was necessary for us to comply with the pandemic research and health policy restrictions put in place by our institutions across 3 continents as the research will be conducted even under pandemic surge conditions. Phase 2 will again use mixed methods; in the quantitative phase 2, field observations will be performed with 30 individuals at each site. In simulation and field testing we aimed for usability numbers and not for statistical significance. A sample size of 30 participants for the quantitative field testing phase was determined using a usability calculation for the conditions across 2 user groups (nurses and physicians) and not for statistical power as it would be premature to conduct a full study before feasibility is established and in addition, the sites were funded and equipped adequately for feasibility alone. Later, using the findings from this pilot feasibility work we will be positioned to apply for full study funding.

### Randomization

Participants will be recruited among health care professionals employed in field settings. Participants will be computer randomized and assigned to 1 of 3 PPE conditions on the day of the study and randomization will occur before study and between conditions. The Stanford simulation site will compare the 3 PPE types, namely, A (L-PAPR), B (respirator and face shield), and C (traditional PAPR). São Paulo and Bologna sites will have 1 intervention arm, A (L-PAPR), and 1 comparator arm, B (respirator and face shield, São Paulo) or C (traditional PAPR, Bologna). The randomizing sequence for each site will be AB or BA (São Paulo) and AC or CA (Bologna). A minimum of 10 participants will be recruited in each simulation site. As this is a crossover design, participants will be assigned to the intervention and comparator arms, and they will be randomized to the use of equipment to reduce order effects, a well-documented phenomenon that suggests different orders or times in which the interventions are presented can influence outcomes.

### Recruitment

The investigators will follow recruiting procedures established by their institutional review boards (IRBs). They will work with hospital staff to explain the study to the potential participants for usability testing, interviews, and field observations. The participant may refuse involvement, in which case another potential participant may be approached. Participants will be recruited using posters visible in common clinical areas, via an email to appropriate departments, and by word of mouth from clinical and research staff in São Paulo, Bologna, and Stanford. Interested volunteers can contact the sites for information by email, phone, or in person. During recruitment, participants will be informed that activities in the simulation phase will occur outside of their routine work shift, to avoid disruption of inpatient care. Participants will be informed about the times and dates of the simulation in advance. Participants will be compensated according to the policies approved for this study by the hospital and university IRBs and their time will be protected to enable them to complete these tasks.

### Consent

Participants will sign an informed consent form in their native language. The risk to participants is less than what they encounter in a clinical workday. Before signing, the participants who gave consent will receive detailed information on the types of PPE and monitoring devices to be used, including photographs, infographics, and instructional videos to facilitate their understanding of the methods to be employed. The participants will be informed that they can withdraw at any time of the study. Each participant will be provided with a unique code to protect their anonymity. The investigators will ensure participants understand the implications of their involvement (risk and benefits), their right to withdraw, that their participation is voluntary, and how information collected during the study will be used and reported. Participants’ comprehension of the materials will be tested using teach-back methods and a questionnaire. The informed consent script “Verbal Consent Script to Inform Participants in a Research Study: Field Observations” will be used to guide this discussion and versions for each country can be accessed from Multimedia Appendix 1.

### Phase 0: Preparation

In this preparatory phase, we will plan a consultation with the WHO for an internal equipment review, and we will test the chosen technology on-site, define data collection, prepare our cross-country site communication network, translate forms to be usable in all site languages, and prepare materials for institutional and WHO IRB approvals (Multimedia Appendix 2).

### Phase 1: Simulation Phase

The simulation will be performed in the institution’s simulation laboratories according to different tasks. The methods for tasks will be as uniform as possible and the fine-tuning of these methods will be augmented during weekly communications with study investigators and research personnel across sites. Participants will use instructional videos and a quick guide, translated into local languages to avoid the effect of excess errors or discomfort that could be attributed to a learning curve that may not occur when participants are familiar with the equipment.

All participants will wear smartwatches while performing the tasks to capture consistent heart rate (HR) and movement (number of steps). Participants will use the smartwatches during a 24-hour period before or after the simulation study to establish baseline physiological parameters. Following the simulation shift, researchers will administer the National Aeronautics and Space Administration Task Load Index score (NASA-TLX) questionnaire [17] and the System Usability Scale (SUS) questionnaire [18]. The SUS measure is validated for perceived usability of the equipment, while the NASA-TLX is used to measure the cognitive load or thinking effort that the equipment requires while using it. These measures combined can offer us a rough estimate of how useful the equipment is from a human factors perspective. Following this, the researchers will conduct a 15-20-minute semistructured interview to elicit human factors related to the success and failure of PPE implementation. Further details about conducting all steps, including adapted questions and time recording per task, are presented in Multimedia Appendix 1.

The Stanford Simulation Lab located within The Stanford Anesthesia Informatics and Media Lab will accommodate additional tasks. The Stanford Lab is self-contained with computer equipment for testing participants and video equipment for recording the research tasks to review observer accuracy. In this setting, objective confirmation of hearing and visual deficits can be computer tested. Visual acuity will be tested with Snellen charts [19] for distance vision and the Jaeger chart [20] will be used to test reading clarity. The Snellen chart is used in schools’ optical stores and many workplaces as a fast validated measure of how far people can see accurately. The Jaeger test asks participants to read different sizes of print to test for reading acuity. Auditory acuity will be tested with the American National Standards Institute–certified Modified Rhyme Test (MRT) [21] word list containing 300 words [22]. From this list, randomized sets of 75 words are generated and participants will be tested in the control condition of no PPE and in each of the 3 conditions they are randomized to. Each set contains 6 monosyllabic words with the same initial consonant. This testing can reveal where language communication has deficits [21]. We will report if auditory deficits increase during the use of any of the 3 PPE conditions. The results will be compared with qualitative feedback and the recording of communication errors during donning, doffing, and medical tasks while performing simulations and in the field.

### Phase 2: Field Testing

#### Steps Overview

Phase 2 field testing will follow simulation testing so that early observations can be applied, and conditions adapted to improve field testing. Over a single shift of direct patient care, the following tasks will be performed, and they will be timed per task and participant. Our hope is that timing the tasks will contribute to assessing how errors occur; for example, we will report whether errors are task related, whether tasks with longer run times produce increased errors, or if errors occur early in the process and become less likely as health care professionals become accustomed to using PPE or performing specific tasks.

#### Step 1

Participants will be supplied with smartwatches, which will allow their movement (eg, number of steps) and HR to be recorded and automatically uploaded to the smartphone software before being recorded on the smartwatch company servers.

#### Step 2

Health care professionals will don the PPE to which they are randomized. Researchers will observe the health care professionals using each PPE combination and will record the instances of PPE readjustments (eg, repositioning of the mask) and user or equipment errors. If the health care professional removes the PPE combination for reasons unrelated to the clinical task, this event and the time of occurrence will be recorded.

#### Step 3

Following the simulation shift, researchers will administer the NASA-TLX questionnaire [17] and the SUS questionnaire [18]. Following this, the researchers will conduct a 15-20-minute semistructured interview to elicit human factors related to the success and failure of PPE implementation. For those seeking more details for conducting all steps including time recording per task, see Multimedia Appendix 1. Table 1 is an approximation of time for tasks, and this was piloted across the sites.

**Table 1:** Tasks time; comparator and intervention simulation will be randomized.

Start	Stop	Time	Description
		0:15	Sign consent forms and study check-ins
		0:05	Donning Change into PPE^a^ and smartwatch function check
		0:15	Acclimation period Take baseline vitals
		0:20	Stanford only, visual and auditory testing
		0:35	Medical Task Series
		0:05	Doffing
		0:20	Take vitals Survey (NASA-TLX^b^ and SUS^c^)
		0:20	Qualitative interview
		0:05	Debriefing and checkout
		1:40	Time per PPE session

^a^PPE: personal protective equipment.

^b^NASA-TLX: National Aeronautics and Space Administration Task Load Index score.

^c^SUS: System Usability Scale.

### Outcome Measures

**Figure 3:**
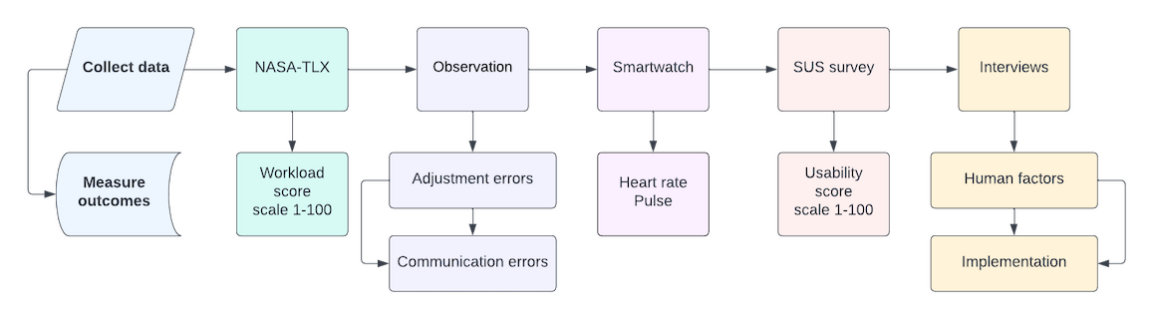
Schema of data collection methods and respective outcomes measures. NASA-TLX: National Aeronautics and Space Administration Task Load Index score; SUS: System Usability Scale.

We will measure the following outcomes, from both phases 1 and 2 (Figure 3):

- The NASA-TLX score [17], on a scale of 0-100, with higher scores indicating higher perceived workload.
- Health workers’ PPE adjustments and the number of errors over a single continuous wear episode (phase 1) or shift (phase 2).
- Errors to observe will include interpersonal communication hindrances during the activities and equipment flaws or breakdowns (phase 1 or 2)
- HR differences as a proxy for stress and the number of steps as a proxy for physical demand. HR and steps will be compared with participant-reported stress outcomes, errors, and time duration of tasks.
- NASA-TLX [17] and SUS [18] questionnaires will be administered and these use a scale of 0-100, with higher scores indicating the best overall usability of the system under study.
- Human factors potentially affecting the implementation of L-PAPR as PPE for health care will be gathered using a semistructured qualitative interview analyzed and coded in the native language and then in English across sites.

### Data Collection

Prior to simulation and field testing, we will provide a video-assisted training procedure with teach-back feedback to check participant comprehension. The functional simulation sequence can be found in Multimedia Appendix 1. For field testing, participants will be observed during 1 work shift.

During simulation and field testing, each participant will be observed by at least two researchers. Researchers will record adjustments, user errors, and communication problems manually or using an electronic tablet. Data collection tools for expected use errors will reserve free-form note taking for observed unexpected use errors. In addition, simulations will be video recorded to allow for verification of the initial data collection.

### Smartwatch Data

HR can serve as a marker for stress and this research will ask participants to wear the smartwatch for 24 hours while not working so that it can serve as a baseline HR without shiftwork wearing PPE. The baseline HR will be collected and compared with HR during the simulation session and the same procedure will take place during field testing and will occur over a single field shift. We will explain as a limitation of our study that it was not possible to control for all environmental effects. Quantitative data for physiological monitoring will be gathered through smartwatches. All sensors are noninvasive: photoplethysmography to monitor HR using infrared light, displacement using the MEMS 3-axis accelerometer, and travel history using connected GPS if attached to a GPS-enabled cell phone. These data are processed to give extra/derivative information: walking and running steps, distance, and calories burnt; HR (beats per minute); connected GPS (distance, pace, and elevation); and sleep (deep and light sleep phases and sleep interruptions) [23].

The reliability of the smartwatches was tested against the validated Hexoskin [24] wearable vests for HR and movement and pulse oximeters for HR. The Hexoskin [24] collects continuously a 1-lead electrocardiogram (256 Hz) and is equipped with 2 respiratory inductive plethysmography sensors technology (128 Hz each) and a 3-axis accelerometer (64 Hz), generating a high-resolution data set (over 42,000 data points per minute). The Hexoskin vests were consistent and superior to the smartwatches for measuring HR variability and activity; however, the condition of extended wear and the need to officially validate decontamination for pandemic conditions meant that we could not use the Hexoskin vests for pandemic research in the ICU.

All data recorded within the smartwatch are sent to a smartphone and then uploaded to smartphone GDPR (General Data Protection Regulation)- and HIPPA (Health Insurance Portability and Accountability Act)-compliant company servers with secure transmission across countries. The sites have IRB-compliant data agreements in place to protect participant privacy for data collection and storage. The smartwatch accelerometer records step frequency and speed. The processes are compliant with Regulation (EU) 2016/679 General Data Protection Regulation compliance [25] and Brazilian Law of Data Protection number 13.703, 2018 [26].

### Interviews and Questionnaire

Standard validated questionnaires (NASA-TLX [17] and SUS [18]) are administered following the end of the shift or simulation (Multimedia Appendix 1). The questionnaires and interview questions will be piloted to estimate interview duration so that we can best adapt the questions according to end user feedback. Interviews will be conducted by the researchers following the completion of the shift or simulation set and will contain 12 questions. The interview, containing 12 questions, will be conducted in person or by videoconferencing and will be digitally recorded and transcribed verbatim in each native language and then analyzed by native speakers. Researchers from each language will agree on the representative accuracy of the coded materials translated into English and when there is not a match this will be reported, and the differences explained. In this way, the research can account for cultural and language variances. The interview questionnaire was developed based on the Consolidated Framework for Implementation Research [27] proposed by Damschroder et al [28]. All data will be centralized to a secure REDCap (Research Electronic Data Capture) [29] database with site-specific access and data collection forms. The data flowchart shown in Figure 3 will be used for simulation and field testing.

### Project Management

All project members meet remotely every week to work through advances and challenges together and to provide methodological support to remain aligned with the protocol. The principal investigators (PIs) will hire and train researchers, regulate safety conditions, and oversee the data collection and analysis. The coinvestigators will support site preparation for phase 1 and the development of phase 2. The researchers will prepare data collection tools and perform data collection and ensure the materials required are adequate and in good working condition for use in the simulation and in the field. Regular meetings between sites, PIs, and researchers will occur to ensure homogeneous data collection procedures and timely follow-up.

### Safety Considerations

All PPEs will be certified according to international standards (the EN 12942 [30] or EN 149 standard [31] for respiratory protective devices). Power-assisted filtering devices incorporate full face masks, half masks, or quarter masks for P5 filters [8]. Additional testing with spectacle usage for the L-PAPR was carried out by BSI UK Labs to assure safety and usability for our participants. The light PAPRs, or PAPRs without a hose, were tested for fit (inward leakage) with participants who wore eyeglasses, under laboratory-controlled conditions and using the test method of the EN 12941 standard. This type of PAPR typically conforms to a maximum allowable total inward leakage rate of 0.05% when on, with positive pressure, compared with a nonpowered, negative pressure filtering facepiece respirator rated at the European Filtering Face Protector (FFP2) standard of less than 11% leakage. BSI UK Labs carried out leakage testing on 10 human participants. No participant experienced a fit [8] with leakage of more than 0.3518%. This leakage is minimal and well below that of an FFP2 filtering facepiece respirator at 8% or 5% N95, assuming completely leakproof or perfect fit [9,10].

During the simulation phase, the number of participants and research assistants in the simulation laboratory will be limited to allow for physical distancing. Research personnel and participants will be prescreened by COVID-19 antigen testing with brands approved by each hospital setting, country and university policy, symptom checking, and vaccination status. All involved personnel will be provided with N95 respirators and alcohol hand sanitizer. In the field testing, all measures to avoid potential infection risk will be taken, according to the local infection prevention and control recommendations for the activities in the hospital setting. Researchers will wear N95 respirators and ICU attire and use alcohol hand sanitizer. All devices and surfaces used among participants during the simulation and in the field will be cleaned and disinfected with adequate sanitizers, before, between, and following uses as appropriate.

### Quality Assurance

Researchers are trained to ensure consistency in methods, ethics, and data management. Participants will be trained on the use of PPE equipment they will use in the study. We will hold weekly video meetings between the 3 sites with researchers, PIs, and invited experts, to problem-solve and consolidate data collection to ensure standardization. Additional support will be provided as additional needs arise and need to be addressed. Investigators will also communicate by email, phone, and in person when feasible.

### Data Management and Governance

Questionnaires are hosted in the REDCap software [29]. We will compare NASA-TLX scores [17] and physiological parameters between the intervention and comparator arms. We will collect HR and the number of steps as an indirect indicator for physical activity and stress over time. Qualitative data will be transcribed verbatim and analyzed as per their content according to Krippendorf [32], identifying themes and categories that emerged from the participants’ answers. Quantitative data will analyze differences in the NASA-TLX scores between the intervention and comparator arms. There is a multisite data management and security plan in place agreed upon by the WHO and individual institutional IRBs. Identifiable data will be replaced with numeric coded identifiers. The codes used for participants in phase 1 and phase 2 will aid anonymity. Only research staff, PI, and co-PIs will know who has declined or withdrawn from the study. The identity of participants or those who decline or withdraw will not be shared outside of the local research group. Data will be stored and kept secure in accordance with IRB agreements and country legislation.

### Public and Patient Involvement

Public and patient involvement is carrying out research with the public rather than on them. Coproduction in research is the action of patients or members of the public becoming partners with the research team and as partners they will cocreate, co-design, and coproduce element of the research with the research team. There is evidence that public and patient involvement along with research coproduction can improve study quality, increase human factors accuracy, and promote health literacy [33,34]. The study was initiated because of the interest and urgency of clinicians, patients, and members of the public concerning human factors and PPE. Their feedback was formative for developing our research questions and methods. University undergraduates, summer interns, parents, and members of the public were invited to comment and coproduce all aspects of the study. They also assisted with survey design, testing, and qualitative analysis. Two first-year undergraduate university students are coauthors (WC and SS).

### Ethics and Institutional Review Board Approvals

The study was submitted and approved by ethical research committees in all 3 sites (Stanford, São Paulo, and Bologna) and by the World Health Organization (WHO) Ethics Review Committee for COVID-19 (Approval #0100). Details are in Multimedia Appendix 1. See also [17,18,27,35-41].

## Results

Our systematic review [42] was completed and informed our protocol. The findings were that PPE implementation involves multilevel transdisciplinary complexity and relies on the development of context-driven implementation strategies. Context-driven strategies can inform and harmonize infection prevention control policy in collaboration with local and international health bodies. The study protocol was presented internally by video and slideshow in fall 2021 to the WHO, Infection and Prevention Control. Stanford is in the preparation and recruiting phase. Bologna and São Paulo have recruited a total of 80 participants. Preliminary results of simulation and the field study from the São Paulo ICU site were presented at ECCMID in April 2022 [43]. The preliminary data show that well-being and comfort are increased with the use of PAPRs by decreasing respiratory effort and eliminating heat accumulation. Participants reported communication difficulties, the noise generated by positive airflow, and facial discomfort with the use of the light PAPRs. The expected publication date for the full multisite study results is December 2022 or the first quarter of 2023.

## Discussion

### Expected Findings

Prior findings highlight the need for human factors research on PPE. Health care professionals on extended shifts report skin breakdowns, headaches, discomfort, and temporary vascular changes while wearing the N95 respirator [42-46]. We found that manufacturers were open to our suggestions for product improvement based on what we learned while getting the protocol ready and that they are assessing how to improve PPE human factors, safety, and functional use. We will supply them with concrete data following full data collection and provide them with ongoing feedback.

In another example, one of the PAPR designs required a customized 3D-printed add-on for those with spectacles which added to the cost of the device. Approximately 70% of the US working population needs corrective vision according to the Vision Council 2021 first quadrant report [47]. The research team developed an idea that provided a simple and cost-free solution that the manufacturer tested at an NIOSH-approved lab. The solution was viable and demonstrates the cost-saving benefits of including all stakeholders and being transparent with them from the inception of a study.

The expected outcomes of this research are a human factors analysis across conditions and sites. The investigators uncovered this need through the DeMaND study, which included 52 investigators [46]. We will explore the design improvement of PPE for facial protection, through outcomes such as improvement in perceived workload (mental demand, physical demand, temporal demand, performance, effort, and frustration), experienced stress via HR variability proxy, and a decrease in the need to readjust PPE indicating better ergonomic compatibility. We will report how the qualitative survey aligns with the quantitative measures. This provides details on where manufacturers can use this research to redesign and adapt their products for better usability. The use of human factors, where design is adapted for human preferences, is preferable to expectations that humans will adapt to existing technology [48]. To assess human factors, this study will use quantitative, self-reported metrics related to workload and usability scores. Participants will be engaged across multiple tasks using 3 forms of PPE so they can compare the equipment for breathing, comfort, and ease of use.

### Limitations

As stated in the “Methods” section, we note the limitations regarding using the smartwatch as a reliable indicator for HR variability that can be correlated with stress. The internal mechanisms of the smartwatch were not evolved to capture this with reliability, so as an alternative we collected steps and HR with a 24-hour baseline for comparison. Our team has persevered through personal loss, pandemic restrictions, reduced access to laboratory facilities, administrative funding delays, and supply chain shortages. This is especially challenging since this research will be conducted across 3 continents.

### Data Transparency

Deidentified aggregated data will be made available to the public with a DOI at the time of results publication.

### Dissemination Strategy

We will share study results with participants and staff in the health care settings where they were recruited. Our dissemination strategies include making posters available with summary results for clinic notice boards, and the creation of an easy-to-read summary report through the communication channels of the WHO and the participating institutions. Relevant information for immediate improvements related to the use and adherence to PPE will be discussed with equipment manufacturers and ICU unit managers. The study results will be presented to the WHO Emergencies Program Experts and Advisor Panel for Infection Prevention and Control Preparedness, Readiness, and Response to COVID-19. Results will be shared by the institutions, in scientific meetings, and through social media. The findings will be published in relevant peer-review journals. Dissemination of the results will be discussed in future meetings of the WHO COVID-19 Infection Prevention and Control Research Working Group WHO R&D infection prevention and control pillar. Feedback on the results of the study will be gathered from participants and distributed to key stakeholders.

### Conclusion

We are committed to achieving safe pandemic research throughout the pandemic and we will report the limitations we face as a guide to researchers who will face future pandemic conditions. We are awed by the kindness, gentle humor, and resilience of our team. They make research worth the investment and help us to continue, knowing that the total COVID-19–related deaths as of May 2022 was 6.3 million people. Let us make PPE better by improving human factors. Potential future research consists of a larger, well-powered multinational study. We have applied for additional funding to build a multi-PPE use functional template. Members of our team will rewrite PPE technical manuals for clarity, and we are working on an international PPE plan for implementation considering all stakeholders.

## Appendix

Multimedia Appendix 1 Supplementary Annex to the Study Protocol.

Multimedia Appendix 2 Simulation Methods for Human Factors: Assessment of PPE use by Medical Personnel for COVID-19 Patient Care.

## Abbreviations

GDPR: General Data Protection Regulation
HR: heart rate
ICU: intensive care unit
L-PAPR: lightweight protective air-purifying respirator
MRT: Modified Rhyme Test
N95: National Institute for Occupational Safety & Health (NIOSH)-approved 95% filtration single-use respirator
NASA-TLX: National Aeronautics and Space Administration Task Load Index score
NIOSH: National Institute for Occupational Safety and Health
PAPR: powered air-purifying respirator
PI: principal investigator
PPE: personal protective equipment
REDCap: Research Electronic Data Capture
SUS: System Usability Scale
WHO: World Health Organization
