# Supplementary material for: Perceived Workload Using Separate (Filtering Facepiece Respirator and Face Shield) and Powered Air-Purifying Respirator and Integrated Lightweight Protective Air-Purifying Respirator: Protocol for an International Multisite Human Factors Randomized Crossover Feasibility Study
Source: JMIR Res Protoc. 2022 Dec 1;11(12):e36549. doi: 10.2196/36549 (PMC9756122; doi:10.2196/36549)
Supplement: Multimedia Appendix 1 [file resprot_v11i12e36549_app1.pdf]

1. ANNEXES OF THE STUDY PROTOCOL

1.1 Annex 1: Simulation Protocol

1. ANNEXES OF THE STUDY PROTOCOL 1

1.1 Annex 1: Simulation Protocol 1

1.1.1 Participants 3

1.1.2 Study Protocol, Simulation 3

1.1.3 Distractors 4

1.2 Annex 2: NASA Task Load Score 8

1.3 Annex 3: System Usability Scale 9

1.4 Annex 4: qualitative questionnaire 10

1.5 Background work 11

1.5.1 Scenario-based simulation 13

1.5.2 Clinical Practice Differences across Field Sites 14

1.6 Demographics form (P2) 15

1.7 Participant instructions for using the Withings smartwatch (P3) 16

Participant instructions for using the Withings smartwatch (P3) 16

1.8 Diary of activities while on the watch use (P4) 19

1.9 Check LPAPR Mask Comprehension Form (google forms) 20

1.10 Instructions - Session Check List (R0) 22

1.11 Smartphone and smartwatch data (R1) 25

1.12 N95 and Face Shield usage checklist 26

1.13 LPAPR mask usage checklist (R2) 27

1.14 PAPR mask usage checklist 30

1.15 Observation data collect form (R3_NN95) 32

1.16 Observation data collect form (R3_NLPAPR) 33

1.17 Observation data collection form (R3_PN95) 34

1.18 Observation data collection form (R3_PAPR) 35
1.19 Script of debriefing (R4) 36
1.20 Field Study – Data Collect Form (R5) 37
1.21 Simulation check list (S4) 41
1.22 Scenery Script (S5) 49
1.22.1 Reference: 56
1.23 Arterial blood gas analysis (S6) 56
1.24 Lab exams (S7) 57
1.25 Nurse assessment (S8) 58
1.26 Physican assessment (S9) 59
1.27 Qualitative interview (S11) 61
1.28 Clinical tasks across site (N95 respirator and traditional PAPR) 62
1.29 Clinical tasks across site (L-PAPR) 64

1.1.1 Participants

participants, in groups of 2 or 3 (2 nurses, 1 physician) or working individually on a task.

1.1.2 Study Protocol, Simulation

Series of tasks, for direct care by nurses

Participant wears smartwatch

The researcher facilitates each task with a maximum duration of the task

Record time to don, until time cut-off; Number of hints to complete don

1. The participants will be shown a video of how to don PPE and will teach back to ensure they understand the materials
2. Donning Use WHO standard for donning PPE / Record time to don [5mins]
3. Start IV - involves fine motor skills, visual acuity, attention, HYPOTHESIS: PAPR has a wide field of vision and is more comfortable people will be less distracted and perform fine motor tasks easier [15 mins]
4. Airway and bag ventilation tasks [5 mins] involve fine motor skills, visual acuity, attention, and nd movement HYPOTHESIS: PAPR has a wide field of vision and is more comfortable people will be less distracted and perform fine motor tasks easier. Communications may be limited [15 mins]
5. Listen to the lungs/heart (Nasogastric tube for Bologna site) - HYPOTHESIS: Tests fan noise interfering with auscultation (PAPR), N95 would not. [5 mins]
6. Prone patient - Involves team communication, and flexibility while wearing and expand active dynamic movement while wearing PPE. Give a checklist of steps to the team to prone the patient, forcing team communication. HYPOTHESIS: There will be barriers to each PPE type, which has the least contamination and is best for (Bologna and Sao Paolo)
7. Read volume on urine bag and empty urine bag - Involves crawling on the floor (knock off PAPR or goggles), reading small numbers HYPOTHESIS (fogging of goggles with N95 and readjustment with regular PAPR) [5 mins]
8. Doffing the Use WHO standard for doffing PPE / Record time to doff
9. NASA TLX Questionnaire
10. SUS questionnaire
11. Qualitative interview [20 minutes]

Series of tasks, for direct care by the physician: The participant wears a smartwatch for each task

Record time to don, until time cut-off

Number of hints to complete don

1. Donning Use WHO standard for donning PPE [5 mins]
2. Arrive at the bedside and get patient status: Involves team communication, vis
3. Listen to the lungs/heart - HYPOTHESIS: Tests fan noise interfering with auscultation (PAPR), N95 would not. [5 mins]
4. Check vital signs on the physiological monitor: vision acuity, attention [5 Mins]

5. Modify ventilator settings: involves fine motor skills, visual acuity, and attention [10 mins]
6. Help nurse prone patients - Involves team communication, flexibility while wearing PPE, exertion, and dynamic movement while wearing PPE. Give a checklist of steps the team must perform to prone the patient, forcing team communication. HYPOTHESIS: There will be barriers to each PPE type, which has the least contamination and is best for the purpose [30 mins]
7. Doffing Use WHO standard for donning PPE [5 mins]

1.1.3 Distractors

Visual and auditory distractors are a typical component of clinical care and they do affect accuracy and stress levels. They will be interspersed through all tasks and automatically programmed into the series

Visual: Hit a button once when the red light goes off, hit 2x for blue light, don't hit for yellow light

Auditory: Unexpected noise from the mannequin, another code

Functional Simulation Flow Chart Stanford

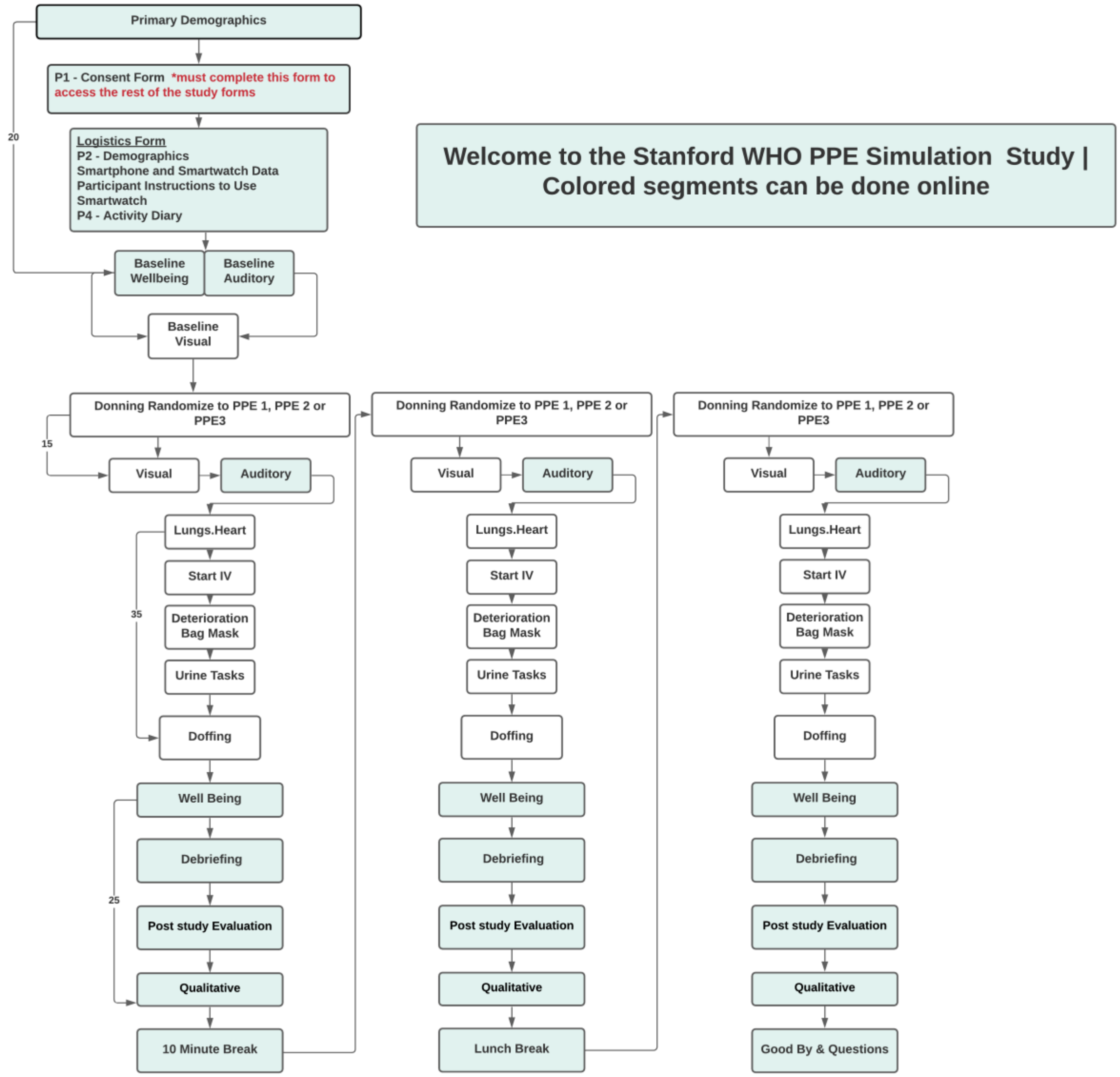

Functional Flow Chart Sao Paulo

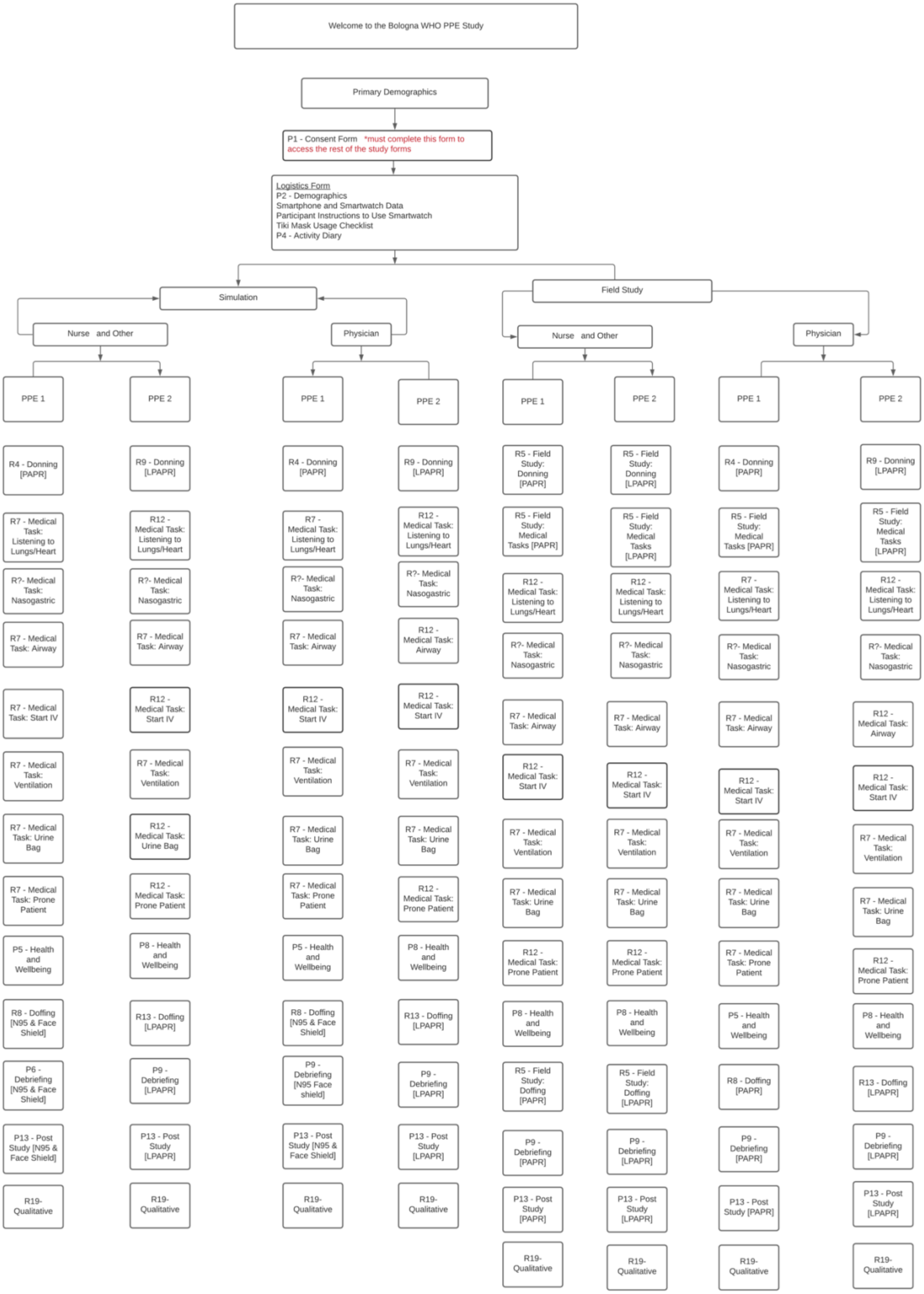

Functional Flow Chart Bologna

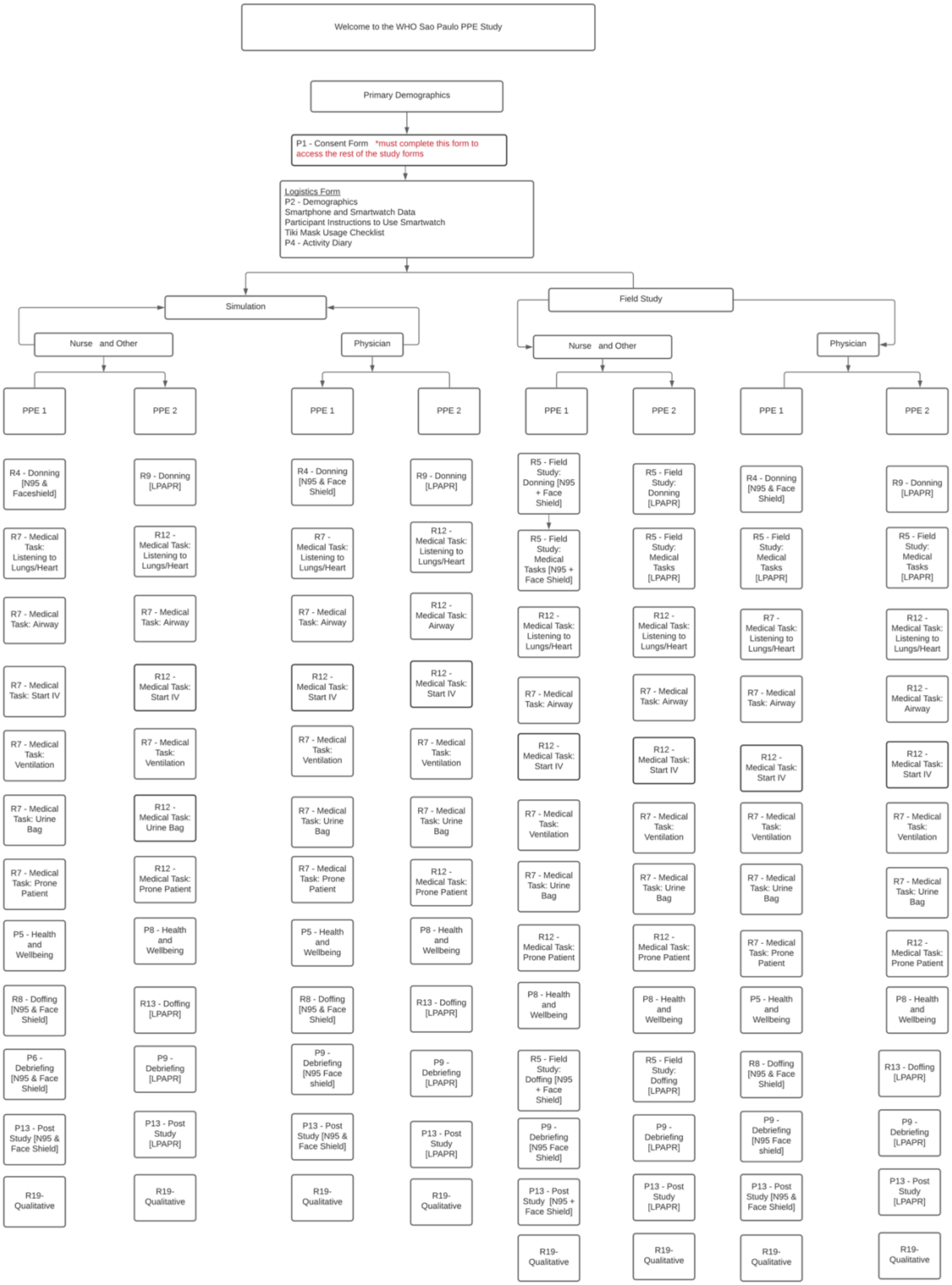

Sao Paulo and Bologna

Tasks: 3 (one physician and 2 nurses) or 2 (one physician and one nurse) participants per session x 2 sessions/day | 5 days | Comparator and intervention simulation will be randomized

Start	Stop	Time	Description
		0:15	Check-in for study and sign consent forms
		0:05	Donning | Change into PPE 1and we are monitoring the watch
		0:15	Acclimation period | note time for baseline vitals
		0:30	Task Series 1: 7 tasks total
		0:15	Survey (NASA-TLX and SUS) | Doffing
		0:05	Donning | Change into PPE 2
		0:15	Acclimation period | note time for baseline vitals
		0:30	Task Series 1
		0:15	Check vitals | Survey (NASA-TLX and SUS) | Doffing. Check-out
		2:25	Study end (total time)

Stanford Simulation task table

Start	Stop	Time	Description
		0:15	Sign consent and check in
		0:05	Donning | Change into PPE and smartwatch function check
		0:15	Acclimation period | take baseline vitals
		0:20	Stanford Only, visual and auditory testing
		0:35	Medical Task Series
		0:05	Doffing
		0:20	Take vitals | Survey (NASA-TLX and SUS)
		0:20	Qualitative interview
		0:05	Debriefing and checkout
		1:40	Time per PPE session

1.2 Annex 2: NASA Task Load Score

NASA - Task Load Score[1]

RATING SCALE DEFINITIONS		
Title	Endpoints	Descriptions
MENTAL DEMAND	*Low/High*	How much mental and perceptual activity was required (e.g., thinking, deciding, calculating, remembering, looking, searching, etc.)? Was the task easy or demanding, simple or complex, exacting or forgiving?
PHYSICAL DEMAND	*Low/High*	How much physical activity was required (e.g., pushing, pulling, turning, controlling, activating, etc.)? Was the task easy or demanding, slow or brisk, slack or strenuous, restful or laborious?
TEMPORAL DEMAND	*Low/High*	How much time pressure did you feel due to the rate or pace at which the tasks or task elements occurred? Was the pace slow and leisurely or rapid and frantic?
EFFORT	*Low/High*	How hard did you have to work (mentally and physically) to accomplish your level of performance?
PERFORMANCE	*Good/Poor*	How successful do you think you were in accomplishing the goals of the task set by the experimenter (or yourself)? How satisfied were you with your performance in accomplishing these goals?
FRUSTRATION LEVEL	*Low/High*	How insecure, discouraged, irritated, stressed and annoyed versus secure, gratified, content, relaxed and complacent did you feel during the task?

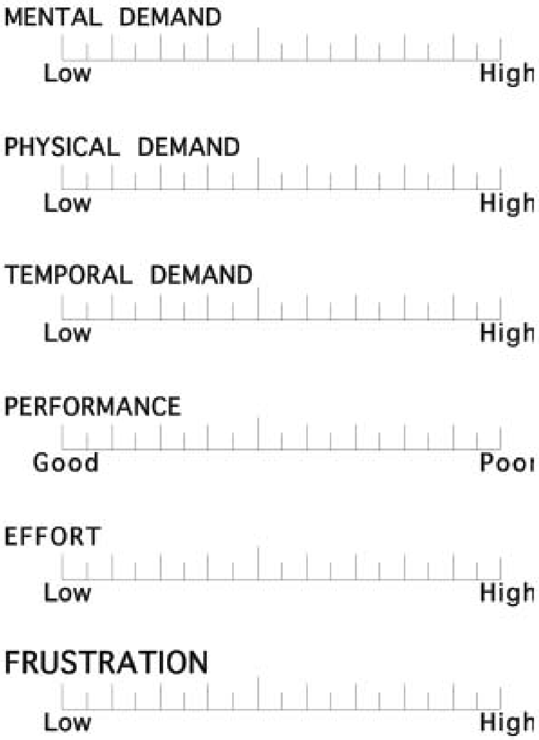

[1] Hart SG, Staveland LE. Development of NASA-TLX (Task Load Index): Results of empirical and theoretical research. Human mental workload. 1988;1(3):139-83.

1.3 Annex 3: System Usability Scale

System Usability Scale[2]

Participant ID: _____ Site: ___________________________ Date: ___/___/___

System Usability Scale

Instructions: For each of the following statements, mark one box that best describes your reactions to the Light PAPR.

		Strongly Disagree				Strongly Agree
1.	I think that I would like to use the Light PAPR frequently.	☐	☐	☐	☐	☐
2.	I found the Light PAPR unnecessarily complex.	☐	☐	☐	☐	☐
3.	I thought the Light PAPR was easy to use.	☐	☐	☐	☐	☐
4.	I think that I would need assistance to be able to use the Light PAPR.	☐	☐	☐	☐	☐
5.	I found the various functions of the Light PAPR were well integrated.	☐	☐	☐	☐	☐
6.	I thought there was too much inconsistency in the Light PAPR.	☐	☐	☐	☐	☐
7.	I would imagine that most people would learn to use the Light PAPR very quickly.	☐	☐	☐	☐	☐
8.	I found the Light PAPR very cumbersome/awkward to use.	☐	☐	☐	☐	☐
9.	I felt very confident using the Light PAPR.	☐	☐	☐	☐	☐
10.	I needed to learn a lot of things before I could get going with the Light PAPR.	☐	☐	☐	☐	☐

Please provide any comments about the Light PAPR:

[2] Brooke J. SUS: a "quick and dirty' usability. Usability evaluation in industry. 1996:189.

1.4 Annex 4: qualitative questionnaire

Questions adapted from: Consolidated Framework for Implementation Research (https://cfirguide.org)

Objective: The purpose of this questionnaire is to capture complementary information regarding the potential implementation of the light PAPR for routine care of patients with infectious diseases.

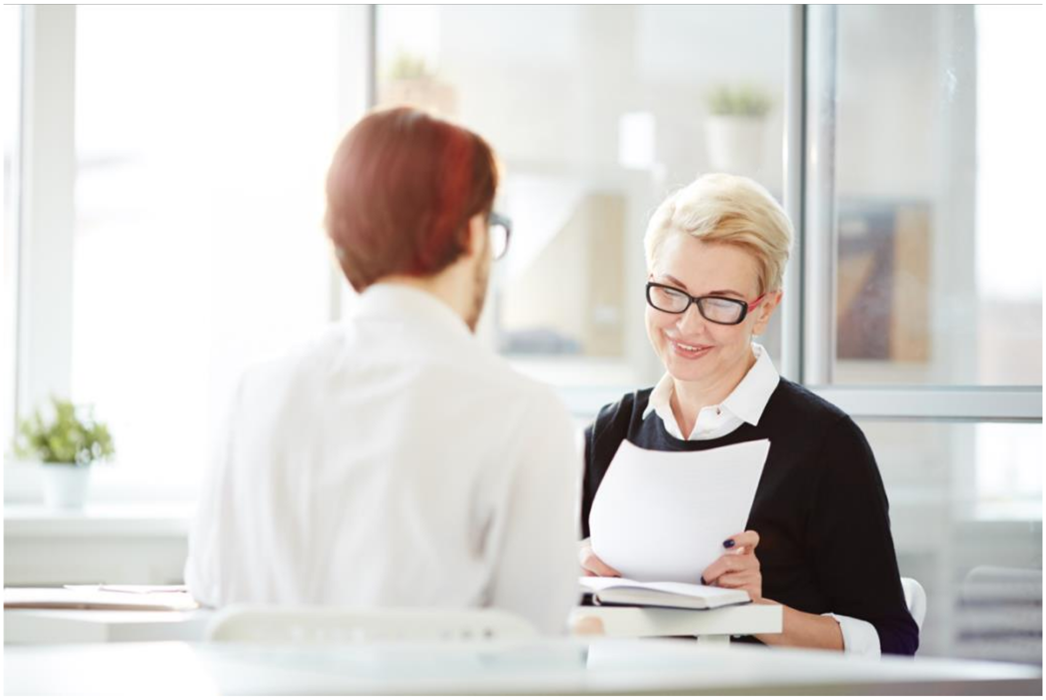

Annex 4, Respondent can be given a selection of photos showing different types of PPE available at the test site including the Light PAPR. This will ensure proper references are made during the interview to the devices being mentioned.

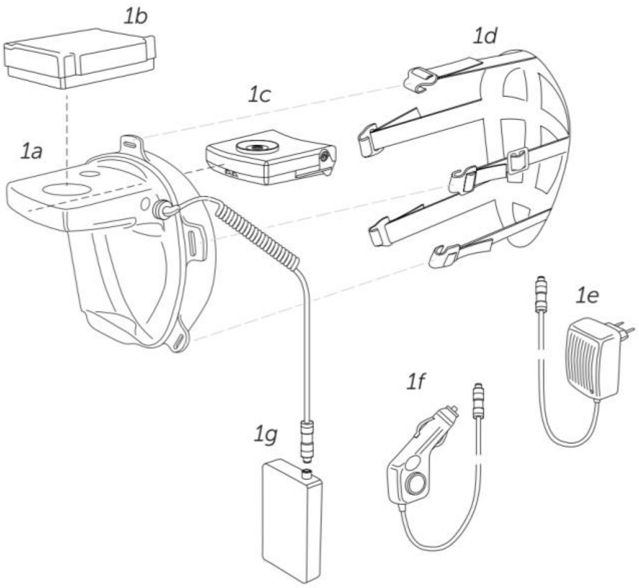

Annex 4, Figure 1 Schematic of the Light PAPR mask component sets

CFIR Domain	CFIR Construct<br>Or<br>Subconstruct	Question (adapted from CFIR Interview Guide Tool)
Intervention characteristics	Relative advantage	Do you perceive any advantage or disadvantage when using the light PAPR compared to other facial protection that you are used to?
		What was your experience when using the light PAPR compared to the other facial protection that you are used to?
	Adaptability	What kinds of changes or alterations could make the light PAPR more effective for use in your setting?
	Complexity	How complicated is the use of light PAPR in comparison to the other PPE you used in this study?
External setting	Patient needs & resources	What extra barriers might patients and their families face if a health workeruses thee light PAPR?
Inner setting	Structural characteristics	What kind of additional infrastructure or process change will be needed to accommodate the implementation of the light PAPR in care for patients with respiratory transmitted diseases?
	Tension for change [subconstruct]	Compared to other PPE do you think there is a strong need to implement light PAPRs for routine work to care for patients with respiratory transmitted diseases?
	Compatibility [subconstruct]	How does the implementation of the light PAPR or other PPE fit with the existingng practices in your setting? What barriers do you think might limit implementation?
Characteristicindividualiduals	Individual stage of change	How prepared are you to use the PAPR in your routine care of patients? Why?
Process	Key stakeholders	Who do you think are the key individuals to get on board to implement the use of the light PAPR for routine care?

Make any comments you think are relevant to the use of light PAPR or other PPE you have tested in this study

The simulation will be performed in four steps:

A) Recruitment of participants and information;
B) Preparation for simulation;
C) Scenario-based simulation;
D) Debriefing.

1.5 Background work

Meetings with the tears essential to align the studies. The coordinator of ICU nurses and the head physician of the ICU are contacted to recruit participants. Check LPAPR masks and Withings smartwatches for damage and replacement. Purchase Fit test kits perform for lightweight PAPRs. A poster is developed with instructions for donning and doffing the LPAPR mask

as well as a video tutorial with the same purpose. Nurses acting as facilitators will be involved to discuss the script of the simulation settings and to conduct training with the mannequin. Record cultural differences between the Brazilian and the Italian site (see Annexes 7,24 and 7.5)..

Participants are contacted by email and provided with an explanation of what the experimentation consisted in, the video tutorial, the banner, the consent form, the sociodemographic forms and smartwatch instructions form.

Preparation for he simulation, the participants will sign the informed consent form and socio-demographic form. All the participants will undertake two fit tests of the Light PAPR mask to ensure that the mask is the right size and sealed properly to the participant's face. The first will be a test with a flat piece of paper placed on top of the mask, as recommended by the manufacturer. The second test will be performed with a commercial kit (FT-30 Qualitative Fit Test Apparatus – Bitter, 3M, EUA for the Brazilian site; FT-10 Qualitative Fit Test Apparatus – Sweet, 3M for the Italian site) that follows the OSHA fit testing requirements for respirators (Figure 3.1).fittest test will be performed according to the manufacturer's guidelines.

The smartwatches will be worn by the participants to measure heart rate and number of steps. To provide a baseline of physiological for the purpose of comparison with the simulation, the participants will be asked to use the smartwatch for 24 hours before or after the simulation session. Participants will be asked to record their activities during the period wearing the smartwatches (sleep, physical exercise, work, meal and others) in a specific form (see item 9.4)

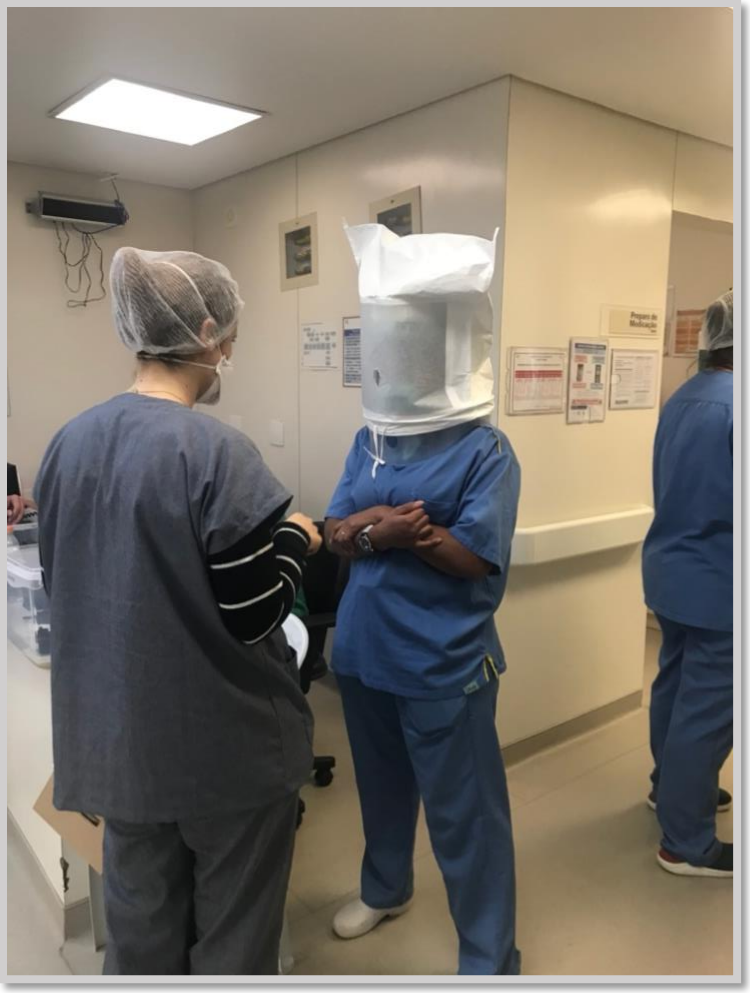

Figure 3. 1. Qualitative Fit Test for Light PAPR. São Paulo, Brazil, 2021.

1.5.1 Scenario-based simulation

I – Site: The simulation will be performed in the ICU unit (currently not in use) of Sant'Orsola Malpighi Hospital. Areas will mimic an ICU: anteroom (donning area) with the specific PPE for the session; nursing station where materials and supplies were available for health care; patient point of care where a mannequin was placed on the hospital bed, a mechanical ventilator, a multiparameter physiological monitor, infusion pumps, hospital tables; and a doffing area, divided into a clean and a contaminated area, where the participants can remove the PPE as well as decontaminated the face shields and the light PAPRs. (Figures 3.2 and 3.3).

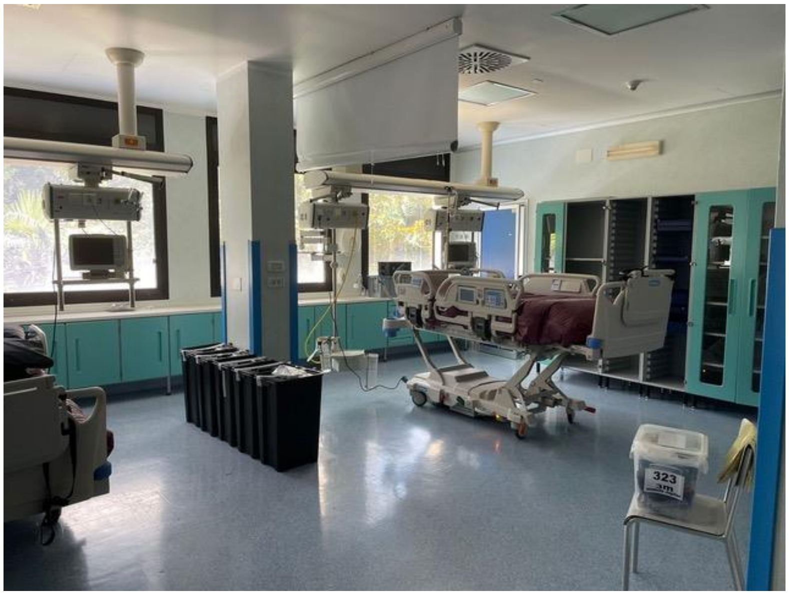

Figure 3.2: ICU unit identified for simulation laboratory setup. Bologna, Italy

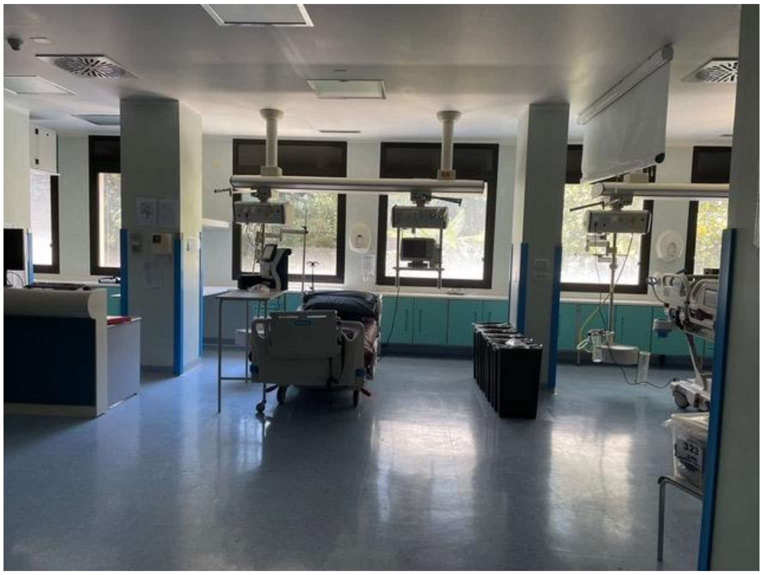

Figure 3.3: Patient care area for simulated patient proning activity. Bologna, Italy

II – Test Sessions: In the simulation phase, the participant sample size will be 10 participants. Five sessions of scenario-based simulations will be scheduled, such that a nurse-physician pair participated in each session. A pre-briefing will be performed to explain the objectives of the session and the sequence of activities, and to assure participants that the study's focus was on the PPE and not to assess the participants' expertise. The simulation scenario including the materials and equipment available will be presented to the participants as well the team and the role of each member will be explained. The activities of each member/participant of the simulation team are described in Table 3.1.

1.5.2 Clinical Practice Differences across Field Sites

Stanford site will conduct objective validated visual acuity and auditory acuity studies and will compare across 3 types of PPE. Stanford will not engage in the proning activity due to legal and policy restrictions. At the Bologna site, one clinical task was replaced and activity modifications were made to reflect the differences in clinical practice and PPE. In addition, traditional PAPRs and the associated donning were elaborated into detailed activities that were more numerous than those for the N95+faceshield PPE combination. The Sao Paulo task of lungs/heart auscultation was replaced with the task of nasogastric tube insertion for nurse participants in Bologna, as this task is not regularly performed by nurses. In this context, nurses rely on the physiological monitor to determine pulse rate. The remaining tasks will be performed at both sites. Detailed task differences are highlighted in red Sections 3.26 and 3.27.

1.6 Demographics form (P2)

FORM NAME	P2 –Sociodemographic Questionnaire			
SUBJECT	Participant’s Sociodemographic Data Collection			
STUDY SITE	☐Brazil ☐ Italy ☐ USA			
DATE	DAY	MONTH		☐ 2021
PPARTICIPANTSNAME	FIRST NAME		LAST NAME	
PARTICIPANT ID				
RESEARCH ASSISTANT NAME	FIRST NAME		LAST NAME	

PART I – SOCIODEMOGRAPHIC CHARACTERIZATION	
1. Gender:<br>a. Male<br>b. Female<br>c. Not binary	
2. Indicate the use of facial hair:<br>a. None<br>b. Mustache<br>c. Beard<br>d. Hair<br>e. Goatee	
3. Age (full years):	
4. Anthropometric data: Weight (kg): _______________ Height (cm): __________________	
5. Profession:<br>a. Nursing technician<br>b. Nurse<br>c. Physiotherapist<br>d. Physician	
PART II – PROFESSIONAL PROFILE	
6. Overall work experience in the healthcare field.<br>a. up to 1 year<br>b.5 to 10 years<br>c.10 to 20 years<br>d.more than 20 years	
7 experience working in the COVID-19 unit:<br>a. Up to 6 months<br>c. Between 1 and 2 years	

8. Job position:<br>a. Direct patient care<br>b. Direct patient care and technical supervision<br>c. Technical supervision	
PART III – EVALUATION OF PERSONAL PROTECTION EQUIPMENT	
9. Do you wear corrective lenses? ( ) Yes ( ) No<br>If yes, what type?<br>a. Corrective glasses<br>b. Transition lenses glasses<br>c. Contact lenses	
10. Please use the space below for any additional comments:	

Source: Adapted from GONZÁLEZ-GIL, María Teresa et al. Nurses' perceptions and demands regarding COVID-19 care delivery in critical care units and hospital emergency services. Intensive and Critical Care Nursing, v. 62, p. 10Instructor1.

1.7 Participant instructions for using the Withings smartwatch (P3)

FORM NAME	P3 - Form for watch use			
SUBJECT	Participant instructions for using the Withings smartwatch (P3			
RESEARCH LOCATION	☒ Brazil ☐ Italy ☐ USA			
DATE	DAY	MONTH		☐ 2021
PARTICIPANT NAME	FIRST NAME		LAST NAME	
PARTICIPANT ID				
RESEARCH ASSISTANT NAME	FIRST NAME		LAST NAME	
SMARTWATCH / SMARTPHONE REGISTRATION NUMBER	ENTER REGISTRATION NUMBER			

Dear participant, the purpose of using this watch is to monitor your heart rate and the number of steps, as an approximate way to evaluate the stress and physical effort in a period of 24 hours before and after the use of the PPE that is going to be tested.

To carry out this monitoring, non-invasive methods and equipment will be used. The heart rate and step count will be smartwatch sthe mart watch of the brand Withings, which should be used by you according to the guidelines described here. The information that will be collected through the proper use of the watch will be processed by in accordance with

the Brazilian Data Protection Law nº13703,2018,and with the General Data Protection Regulation (GDPR) of the European Union 2016/679.

On Day 0 (zero), the researcher will properly place and position the watch on your wrist. ItYou must remainith the watch properly positioned on your wrist until the end of the period stipulated for use (1 to 3 consecutive days). Do not remove the watch when you go to sleep or when performing any other activity, the watch is waterproof. At the end of the monitoring period, the research assistant will remove the watch that has been placed on your wrist.

Guidelines while using the watch:

To ensure that the smartwatch takes accurate captures of your heart rate, be careful to position the smartwatch carefully on your wrist. The smartwatch needs to be positioned about a centimeter below the wrist bones and the bracelet should be fastened tightly but comfortably around the wrist. Some activities that apply force in the wrist region may interfere with measurement accuracy. Check the proper positioning of the watch below: Figure below (the left).

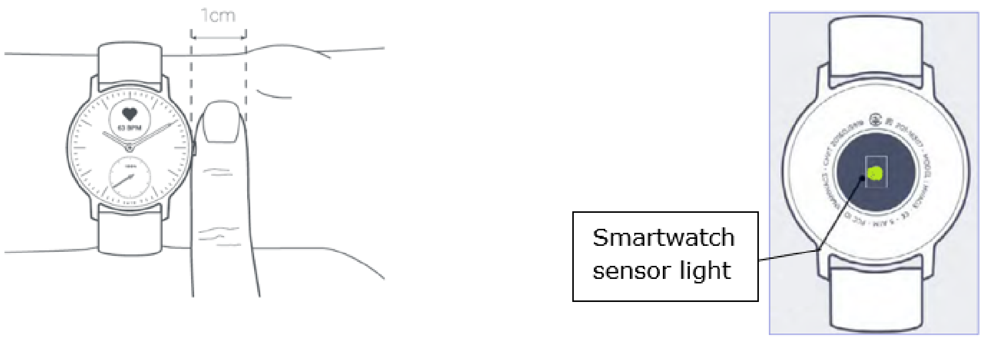

Figure 1 e 2: Adapted figures from User guide – Withings Steel https://support.withings.com/hc/en-us/articles/360015729594-Steel-HR-Steel-HR-Sport- User-Guide

The heart rate measurement technology used by Withings Steel HR is based on capturing the light absorbed by your skin and blood. Any permanent or temporary changes to your skin, such as tattoos, sweat, or even hair located in the region of the smartwatch light sensor, can affect the accuracy of the measurements. Check the location of the smartwatch light.

Water resistance

The watch is water-resistant and withstand pressure to 50 m deep. Therefore, you can use it in activities that contain water, such as hand sanitization, bathing, equipment hygiene

At the time of hand sanitization to start your work shift, also sanitize the display and bracelet of the watch with soap. It is not necessary to remove the watch to carry out this sanitization.

ATTENTION: the smartwatch button should not be pressed when it is submerged or surrounded by water.

To avoid failure or damage according to use, please pay attention to the instructions below:

- Use the product between temperatures -10°C and 45°C;
- Avoid any extreme temperature changes;
- Avoid any kind of shock, whether physical, thermal or otherwise,, as this can damage the internal and external components of your watch;
- Keep the Watch display away from sharp or hard objects to avoid any scratches.

- Do not use the smartwatch in extreme atmospheres, such as saunas or steam rooms, hot water, diving.

Signature:______________________________

Statement of Liability

I,____, VAT nº ____________________, I declare that I have received the equipment described below, to be used by me until _____ (date) of ____________ (month) of 2021, according to the guidelines that were provided to me by the team of researchers of the project "perceived mental and physical workload using traditional powered air purifying respirator and integrated respirator powered air purifying respirator", coordinated by Dr. Fabio Tumietto and Dr. Luciano Attard.

1 Withings brand smartwatch wristwatch, model Steel HR 36mm-HWA03b-36black-inter, MAC address: __________________ (place number).

I undertake to comply with the instructions for use of the equipment, and to return itas soon as the activities are done. In case of robbery or theft, make a police report and immediately communicate the coordination of the project, by email: estudopapr@gmail.com.

Bologna, _ _ _ _ , _____________ 2021.

____

Signature

Full name: ____

ID: ____

Full address: ____

Phone for contact: ____

1.8 Diary of activities while on the watch use (P4)

SUBJECT	P4- Diary of activities while on the watch use			
RESEARCH LOCATION	☒ Brazil ☐ Italy ☐ USA			
DATE	DAY	MONTH		☐ 2021
PARTICIPANT NAME	FIRST NAME		LAST NAME	
PARTICIPANT ID				
RESEARCH ASSISTANT NAME	FIRST NAME		LAST NAME	
SMARTWATCH / SMARTPHONE REGISTRATION NUMBER	ENTER REGISTRATION NUMBER			

Fill the table below with the activity times of the simulation day, as well as the previous and following days. The diary must contain at least three days.

Activities	Time		
	Date:__/__/___	Date:__/__/____	Date:__/__/____
Rest/Sleep	Example: 11pm to 7am	Example: 11pm to 7am	Example: 11pm to 7am
Meal			
Working activity while seated			
Working activity with movement			
Other activities that do not require physical effort			
Other activities that require physical effort			
Lowintensity activity			
Moderate intensity activity			
High-intensity activity			
Driving			

Did you consume caffeine or other stimulants?

If yes, indicate the dates, times and the name of the substance. Example: 7am : a cup of coffee
____

1.9 Check LPAPR Mask Comprehension Form (google forms)

DEAR PARTICIPANT,

WELCOME TO THE RESEARCH OF THE LPAPR MASK!

AFTER READING THE QUICK GUIDE AND WATCHING THE VIDEO WITH THE STEPS OF: ASSEMBLY OF THE LPAPR MASK, PLACEMENT, REMOVA,L, CL EANING AND DISIN,FECTION.

WE KINDLY ASK YOU TO ANSWER THE FOLLOWING QUESTIONS. DOUBTS WILL BE RESOLVED BEFORE THE START OF THE SIMULATION ACTIVITIES.

1) Check the correct alternative: which filter should be placed at the top of the LPAPR mask?

a) The green color exhalatory filter.

b) There is no need to put any filter, as the mask will always be assembled.

c) The inhalation filter of green color.

d) The white color exhalatory filter.

e) The white color inhalation filter

2) Should you use the mask without doing the sealing test? *

( ) True

( ) False

3) Is it necessary to press hard at the inhalation and exhalation filters for the fitting of those to occur at the time of the assembly of the mask? *

( ) True

( ) False

4) When placing the mask, the hood calibration procedure should be done. Mark the correct alternative that indicates how this procedure should be performed: *

a) Before putting on the LPAPR mask, turn on the equipment by holding the power button for 3 seconds.

b) Before putting on the LPAPR mask, adjust the speed of the blower by pressing the power button repeatedly.

c) Before putting on the LPAPR mask, press the power button and the flashlight button simultaneously for 6 seconds until the lights stop flashing.

d) Before putting on the LPAPR mask, turn on the equipment by holding the power and flashlight buttons for 6 seconds.

e) Calibrate the blower with the LPAPR mask on your face, holding the power button and flashlight for 4 seconds.

5) Remove the LPAPR mask by pulling it forward and the straps up. *

( ) True

( ) False

6) For the decontamination of the mask, analyze the sequence below: 1) With a cloth moistened with compatible disinfectant, perform external cleaning of the inhalation (white), expiratory ,(green) filters and the ,outer face of the LPAPR mask. 2) Take a new cloth moistened with disinfectant and perform cleaning of the inner part of the LPAPR mask. 3) Be careful with the inside of the blower. The LPAPR mask blower and filters should never be immersed in disinfectant solution. 4) After decontaminating the LPAPR mask, let it dry until the next use, place the clean where it belongs and and cover it. Ensure that the box is in a decontaminated area designated for storage of the LPAPR mask. After analyzing the text above, answer: this sequence of the mask cleaning and disinfection procedure is: *

( ) Right

( ) Wrong

1.10 Instructions - Session Check List (R0)

Name	R0 – Instructions – Session Check-list
Topic	Instructions about steps in simulation study and field study

Simulation Study - Morning					
Time	Duration (min)	Objective	Activity	Form(s)	Responsible
	0:00	Recruitment and acceptance	Send an email to participants with the invitation and LPAPR mask instructions video	☐ Recruitment poster for the simulation (S1), Consent form – simulation study (P1), LPAPR Quick Guide (S2), Watch form (P3 e P4), Check LPAPR Mask Comprehension Form (Google forms)	
	0:05	Day-Before	Participant Welcome and consent form signed	☐ Consent form – simulation study (P1)	Two research assistants (according scale)
	0 to:10		Orientation and demographics	☐ Demographics form (P2)	
	0:10		Smartphone and smartwatch orientation	☐ Watch form (P3 e P4) and Smartphone and Smartwatch data (R1 e S3)	
	0:10		Fit Test -3M and LPAPR Mask (paper)	☐ Demographics form (P2)	
	Total= 35				
08:00	0:30	Organization	Staff meeting and check setting	☐ Simulation check-list – pre-briefing (S4)	Coordinator, facilitators and o,bserver
Participants arriving					
08:20	0:10	Organization	Reception and Change clothof es	No form	
08:25	08:30	Organization	Team huddle	No form	Coordinator,, facilitators and observers
08:30	0:10	Pre-Simulation	O,rientation about procedures, areas and equipment for simulation	☐ Scenery script (S5)	Coordinator
08:40	0:15		Think aloud method and assembly PAPR	☐ PAPR assembly form (R2)	Coordinator and Observers
08:55	0:60	Simulation	Beginning observation PPE 1	☐ Support forms (S6-S10)	Observers and facilitators
			Clinical Tasks: PPE 1	☐ Observation Data Collect Form (R3)	Observers
09:55	0:10	Post-Simulation	PPE/PAPR SURVEY: PPE 1	☐ NASA/SUS forms (R4)	
10:05	0:60	Break	Break & Meal	No form	

11:05	00:05	Organization	Team huddle	No form	Coordinator, facilitators and observers
11:10	0:60	Pre-Simulation	Beginning observation: PPE 2	☐ Support forms (S6-S10)	Observers and facilitators
		Simulation	Medical Tasks: PPE 2	☐ Observation Data Collect Form (R3)	Observers
12:10	0:10	Post-Simulation – Participant 1	Debriefing - Participant 1	☐ Debriefing script (R4)	
12:20	0:05		PPE/PAPR Survey: PPE 2 - Participant 1	☐ NASA/SUS forms (R4)	
12:25	0:20		Qualitative interview – - Participant 1	☐ Qualitative form (S11)	
12:40	0:10	Post-Simulation – Participant 2	Debriefing - Participant 2	☐ Debriefing script (R4)	
12:50	0:05		PPE/PAPR Survey: PPE 2 - Participant 2	☐ NASA/SUS forms (R4)	
12:55	0:20		Qualitative interview – - Participant 2	☐ Qualitative form (S11)	
13:15	Total = 290				
	0:10		Tea,m huddle		Coordinator, facilitators and observers
Simulation Study – Afternoon					
Time	Duration (min)	Objective	Activity	Form(s)	Responsible
13:00	00:30	Organization	Staff meeting and check setting	☐ Simulation check-list – pre-bri,efing (S4)	Coordinator, facilitators and observers
Participants arriving					
	00f :10	Organization	Reception and Change clothes	No form	
13:25	13:30	Organization	Team, huddle	No form	Coordinator, facilitators and observers
13:30	0:10	Pre-Simul,ation	Orientation about procedures, areas and equipment for simulation	☐ Scenery script (S5)	Coordinator
13:40	0:15		Think aloud method and assembly PAPR	☐ PAPR assembly form (R2)	Coordinator and Observers
13:55	0:60	Simulation	Beginning observation PPE 1	☐ Support forms (S6-S10)	Observers and facilitators
			Clinical Tasks: PPE 1	☐ Observation Data Collect Form (R3)	Observers
14:55	0:10	Post-Simulation	PPE/PAPR SURVEY: PPE 1	☐ NASA/SUS forms (R4)	
15:05	0:60	Break	Break & Meal	No form	
16:05	00:05	Organization	Tea,m huddle	No form	Coordinator, facilitators and observers
16:10	0:60	Pre-Simulation	Beginning observation: PPE 2	☐ Support forms (S6-S10)	Observers and facilitators
		Simulation	Medical Tasks: PPE 2	☐ Observation Data Collect Form (R3)	Observers

17:10	0:10	Post-Simulation – Participant 1	Debriefing - Participant 1	☐ Debriefing script (R4)	
17:15	0:05		PPE/PAPR Survey: Ppe 2 - Participant 1	☐ NASA/SUS forms (R4)	
17:20	0:20		Qualitative interview – - Participant 1	☐ Qualitative form (S11)	
17:40	0:10	Post-Simulation – Participant 2	Debriefing - Participant 2	☐ Debriefing script (R4)	
17:50	0:05		PPE/PAPR Survey: PPE 2 - Participant 2	☐ NASA/SUS forms (R4)	
17:55	0:20		Qualitative interview – - Participant 2	☐ Qualitative form (S11)	
18:15	Total = 290				
	0:10,		Team huddle		Coordinator, facilitators and observers
Field Study Session					
Time	Duration (min)	Objective	Activity	Form(s)	
	0:10	Field study	Participant Welcome and consent form signed	☐ Consent form – field study (P5)	
	0:10		Orientation and demographics	☐ Demographics form (P2)	
	0:150		Beginningcollectionation: PPE 1	☐ Field Study – Data collect form (R5)	
	0:10	Post-Observation	PPE/PAPR Survey: PPE 1	☐ NASA/SUS forms (P4)	
	0:60	Break	Break and change of PPE		
	0:150		Beginning collection: PPE 2	☐ Field Study – Data collect form (R5)	
	0:10	Post-Observation	Debriefing	☐ Debriefing script (R4)	
	0:10		PPE/PAPR Survey: PPE 2	☐ NASA/SUS forms (R4)	
	0:20		Qualitative interview	☐ Qualitative form (S11)	
	Total = 430				

1.11 Smartphone and smartwatch data (R1)

FORMS NAME	R1 – Smartphone and smartwatch data		
CONTENT	Smartphone and smartSTUDY time stamp data; Synchronization		
SUTDY SITE	☐Brazil ☐ Italy ☐ USA		
DATE	DAY	MONTH	☐ 2021
NAME OF THE PARTICIPANT	FIRST AND MIDDLE NAME	LAST NAME	
PARTICIPANT ID			
NAME OF THE RESEARCH ASSISTANT	FIRST NAME	LAST NAME	

1. Fill in an X to indicate whicTimestamp used by this participant:

2. Time stamp of the activation and deactivation of the watch workout mode and confirmation of the synchronization of the watch data with the smartphone:

Simulation phase:
Enter the time of the workout mode activation ____:____
Enter the time of the workout mode deactivation ____:____
Was the synchronization of the watch with the smartphone performed right after the end of the simulation? YES ( ) NO ( )

Field phase (ICU):
Enter the time of the workout mode activation ____:____
Enter the time of the workout mode deactivation ____:____
Was the synchronization of the watch with the smartphone performed right after the enparticipant'sivities performed in the participants work shift? YES ( ) NO ( )

Conclusion of the monitoring period:
Was the synchronization of the watch with the smartphone performed?
YES ( ) NO ( )

1.12 N95 and Face Shield usage checklist

FORMS NAME – N95 Respirator and Face Shield usage checklist

CONTENT	N95 and Face Shield Donning and Doffing		
STUDY SITE	☐Brazil ☐ Italy ☐ USA		
DATE	DAY	MONTH	☐ 2021
NAME OF THE PARTICIPANT	FIRST AND MIDDLE NAME	LAST NAME	
PARTICIPANT ID			
NAME OF THE RESEARCHER	FIRST NAME	LAST NAME	

Start time of this activity	___________________

Initial instructions N95 and Face Shield
☐ Did you read the quick instruction guide, watch the video and answer the understanding questions?
Donniyour ng N95 and Face Sthe hield
☐ Have you tied hair up and back from face?
☐ Did you perform hand hygiene before the seal test?
☐ Did you put on the surgical cap/hair cover and make sure the ears are covered during patient care?
☐ Did you don the gown and secure the ties with a simple bow?
☐ Did you check that there was no hair and/or cap interference preventing the sealif ?
☐ Have you checked if the fit is correct, and the mask pressure is felt against the face when you inhale and exhale?
☐ Did you apply the Face Shield makin,g sure it is sealed to the forehead, clean, undamaged and that you have good visibility?
☐ Did you apply gloves and make sure they fit and are not damaged?
☐ Did you notice if there were any faults/air leaks in the N95 or damage on the Face Shield? If so, have you tried to find the cause and report this to the researcher?
Doffing N95 and Face Shield
☐ Did you use the disinfectant wipe to clean the standard patient care gloves before

removing the N95 and the gown and discarding them in the appropriate trash?
☐ Did you use the glove-in-glove technique to remove the gloves and discard them in the trash
☐ Did you remove the Face Shield and the Surgical cap and place them in the designated doffing area?
☐ Did you clean the Face Shield with the disinfectant wipe and place it in the designated doffing area?
☐ Did you perform hand hygiene?

Closing time of this activity	___________________

1.13 LPAPR mask usage checklist (R2)

FORMS NAME	R2 – LPAPR mask usage checklist		
CONTENT	Step by step description of LPAPR mask assembly, placement, disassembly and decontamination		
STUDY SITE	☐Brazil ☐ Italy ☐ USA		
DATE	DAY	MONTH	☐ 2021
NAME OF THE PARTICIPANT	FIRST AND MIDDLE NAME	LAST NAME	
PARTICIPANT ID			
NAME OF THE RESEARCH ASSISTANT	FIRST NAME	LAST NAME	

Start time of this activity	___________________

Initial instructions LPAPR
☐ Did you read the quick instruction guide, watch the video and answer the understanding questions (S2)?
☐ Have you performed hand hygiene before the seal test
Sealing test

☐ Have you adjusted the LPAPR mask to the face (without the fan and filters faceplate for comfortable fit?
☐ Have you put the paper on top of the face plate covering the two holes above? Have you covered the side hole with your finger?
☐ Have you tried to breathe carefully?
☐ Have you checked if the fit is correct, and the mask pressure against the face when trying to inhale and exhale? (If so, the LPAPR mask can be used).
☐ Did you notice if there were any faults/air leaks? If so, have you tried to find the cause and if needed, tighten the side straps?
☐ After the seal chec?, did you find air leaks after correcting and adjusting the mask. If so please report this to the research team, the LPAPR is unusable;
☐ Did you perform the 3M seal test? Result: ______________________________________
LPAPR Set-Up
☐ Did you carefully connect the charger to avoid damage to the fragile connector structure?
☐ Did you make sure there was no visible damage or residue on the LPAPR?
☐ Did you check if the LPAPR battery was charged?
☐ Did you connect the inhalation filter (white) to the top of the mask correctly?
☐ Did you connect the exhalation filter (green) on the bottom of the mask correctly?
To reset the LPAPR mask
☐ Did you hold the mask in an inverted vertical position (”upside down“) to reset?
☐ Did you press both buttons simultaneously (power button and flashlight button) until both stop flashing?
Donning the LPAPR mask
☐ Have you put on the mask correctly?

☐ Have you performed adjustment of the mask using first the straps from above?
☐ Were you careful to make sure the mask seal was correct?
☐ Did you check that there was no hair and/or cap preventing the seal?
☐ Did you locate the power and flashlight buttons?
☐ Have you powered on the LPAPR by holding the button power for 3 seconds?
☐ Did you take a deep breath a few times in order to check if the positive pressure calibration inside the mask occurred?
☐ Did you check if the light on the left side was on, but not flashing?
☐ Have you tested the adjustment of the fan speed(which ranges from 1 to 4 clicks)?
☐ Did the light on the right side start flashing? Did you check for adequate internal pressure of LPAPR or low battery ?
Doffing LPAPR mask
☐ Did you remove the LPAPR by pulling the straps from the harness webbing?
☐ Did you remove the LPAPR by pulling up and without touching the LPAPR against your clothes?
☐ Did you avoid touching the face or the front of the LPAPR?
☐ Did you leave the LPAPR in the specified area after doffing until cleaning and decontamination is carried out?
Cleaning the LPAPR mask and filters
☐ Have you removed the filters by turning them 30 degrees to the left?
☐ Have you removed the sealing screw on the left side of the mask?
☐ Did you moisten the cloth with specific disinfectant in sufficient quantity?
☐ Did you start cleaning with specific disinfectant on the outside?

☐ Did you clean the elastic straps carefully with specific disinfectant?
☐ Did you carefully clean the inside of the mask?
☐ Did you remember not to immerse filters in disinfectant?
☐ Did you place the LPAPR in the specified clean area without touching and other object until the next use?

Closing hours of this activity	___________________

1.14 PAPR mask usage checklist

FORMS NAME	PAPR mask usage checklist		
CONTENT	Step-by-step description of PAPR mask assembly, placement, disassembly and decontamination		
STUDY SITE	☐Brazil ☐ Italy ☐ USA		
DATE	DAY	MONTH	☐ 2021
NAME OF THE PARTICIPANT	FIRST AND MIDDLE NAME	LAST NAME	
PARTICIPANT ID			
NAME OF THE RESEARCH ASSISTANT	FIRST NAME	LAST NAME	

Start time of this activity	___________________

Initial instructions PAPR
☐ Did you read the quick instruction guide, watch the video and answer the understanding questions?
☐ Have you performed hand hygiene before the seal test
☐ After the seal check, did you find air leaks after correcting and adjusting the mask. If so please report this to the research team, the PAPR is unusable;
☐ Did you perform the 3M seal test? Result: _______________________________________

PAPR Set-Up
☐ Did you attach battery pack to bottom of motor/blower and hear the click?
☐ Did you place the filter into the cover and install the motor/blower and confirm cover latch is secure? (You will hear it click into place and filter label will show in cover window)
☐ Did you Insert the end of the breathing tube with the two small prongs into slots in the motor/blower air outlet? (Twist the breathing tube ¼ turn to the right to lock and attach the breathing tube to the hood or headcover by pushing the QRS end of the breathing tube (end with the blue pinch clip) onto the air inlet of the headgear and you will hear a click when it is secure).
☐ Did you perform flow check by inserting the air flow indicator into the outlet on the TR-300 motor/blower unit. 1) Start motor/blower and run for 1 minute. 2)With the airflow indicator in a vertical position, bottom of the floating ball must be at, or above, the minimum flow mark.
☐ Did you report to the researcher if floating ball is not above minimum mark? (respirator system is not safe for use)
Donning the PAPR
☐ Have you turned on the motor/blower by pressing and holding the blue on/off button. Next use the belt or backpack to attach PAPR to wearer. Now pull headgear over the head and adjust. Facial hair must not interfere with face seal.
☐ Have you used the belt or backpack to attach PAPR to the wearer?
☐ Were you careful to pull headgear over head and pull the face seal under the chin.
☐ Did you make sure facial hair does not interfere with the face seal
Doffing the PAPR
☐ Did you remove the LPAPR by pulling the straps from the harness webbing?
☐ Did you remove the PAPR by pulling up and without touching the LPAPR against your clothes?
☐ Did you avoid touching the face or the front of the PAPR?
☐ Did you place the LPAPR in the specified clean area without touching and other object until the next use?

Closing hours of this activity	___________________

1.15 Observation data collect form (R3_NN95)

R3 - Collect data form (Obsevation) - Phase 1 Simulation - NURSE N95

ID Participant (number ID and initials): ____________________

What PPE did the participant use in the session just completed? ____________

Tasks: expected time = 60 min

TASKS	Effectiveness of tasks performed without assistance (yes, no or not applicable)	Type of help / assistance (verbal / physical / both)	Number of errors	Number of PPE readjustments	Comunnication problems between the participants (yes or no)	Additional observations (describe)
**1 - Donning N95 and Faceshield (1) (expected time: 5 min)**						
**Start time: ____________**						
Perform Hand hygiene						
Donning the Gown						
Respirator: Prestretch top and bottom straps before placing respirator on the face						
Cup the respirator in your hand, position the respirator under your chin; Pull the bottom strap over your head and position it around the neck below the ears; Adjust the metal nosepiece						
To check the respirator-to-face seal, place both hands completely over the respirator and exhale sharply.						
Place the faceshield and adjust it to a comfortable position						
Put the surgical cap						
Put the gloves						
Total (for each task)						
**2. Listen to lungs-heart (3,4) (expected time: 5 min)**						
**Start time: ____________**						
Hand hygiene						
Listen to lungs and heart						
Perform stethoscope disinfection						
Hand hygiene						
Register the patient assessment						

1.16 Observation data collect form (R3_NLPAPR)

R3 - Collect data form (Obsevation) - Phase 1 Simulation - NURSE TIKI MASK

ID Participant (number ID and initials): ____________________

What PPE did the participant use in the session just completed?____________________

Tasks: expected time = 60 min

TASKS	Effectiveness of tasks performed without assistance (yes, no or not applicable)	Type of help / assistance (verbal / physical / both)	Number of errors	Number of PPE readjustments	Comunnication problems between the participants (yes or no)	Additional observations (describe)
**1 - Donning Tiki Mask (1) (expected time: 10 min) -**						
**Start time: ____________**						
Perform Hand hygiene						
Donning the gown						
Be careful with the fragile charger connector						
Ensure there was no visible damage or debris on the PAPR						
Check if the PAPR is charged						
Position the internal blower, the inhalation filter (white) and exhalation filter (green) correctly						
Reset the blower, pressing both buttons (on/off and the flashlight button) simultaneously until both buttons stop blinking						
Place the PAPR correctly, adjusting the straps						
Check that there was no hair or cap impeding the seal						
Turn on the PAPR by holding the power button for 3 seconds						
Take a few deep breaths to check that the positive pressure inside the PAPR was calibrated						
Check if the left light is on and not blinking						
Adjust the speed of the ventilator (1 from 4)						
Put the surgical cap						
Put the gloves						
Total (for each task)						
**2. Listen to lungs-heart (3,4) (expected time: 5 min)**						
**Start time: ____________**						

1.17 Observation data collection form (R3_PN95)

R3 - Collect data form (Obsevation) - Phase 1 Simulation - PHYSICIAN N95

ID Participant (number ID and initials): ______________________

What PPE did the participant use in the session just completed?______________

Tasks: expected time = 60 min

TASKS	Effectiveness of tasks performed without assistance (yes, no or not applicable)	Type of help / assistance (verbal / physical / both)	Number of errors	Number of PPE readjustments	Comunnication problems between the participants (yes or no)	Additional observations (describe)
**1- Donning N95 and Faceshield (1) (expected time: 5 min)**						
**Start time:** ____________						
Perform Hand hygiene						
Donning the Gown						
Respirator: Prestretch top and bottom straps before placing respirator on the face						
Cup the respirator in your hand, position the respirator under your chin; Pull the bottom strap over your head and position it around the neck below the ears; Adjust the metal nosepiece						
To check the respirator-to-face seal, place both hands completely over the respirator and exhale sharply.						
Place the faceshield and adjust it to a comfortable position						
Put the surgical cap						
Put the gloves						
Total (for each task)						
**2. Arrive at bedside e get patient status (expected time: 5 min)**						
**Start time:** ____________						
Approach the patient, introduces him/herself anch assess the general health status of the patient						
Total (for each task)						
**3. Check vital signs on physiological monitor (expected time: 5 min)**						
**Start time:** ____________						

1.18 Observation data collection form (R3_PAPR)

R3 - Collect data form (Obsevation) - Phase 1 Simulation - PHYSICIAN TIKI MASK

ID Participant (number ID and initials): ______________________________

What PPE did the participant use in the session just completed?__________________

Tasks: expected time = 60 min

TASKS	Effectiveness of tasks performed without assistance (yes, no or not applicable)	Type of help / assistance (verbal / physical / both)	Number of errors	Number of PPE readjustments	Comunnication problems between the participants (yes or no)	Additional observations (describe)
**1 - Donning Tiki Mask (1) (expected time: 10 min) -**						
**Start time:** ____________						
Perform Hand hygiene						
Donning the gown						
Be careful with the fragile charger connector						
Ensure there was no visible damage or debris on the PAPR						
Check if the PAPR is charged						
Position the internal blower, the inhalation filter (white) and exhalation filter (green) correctly						
Reset the blower, pressing both buttons (on/off and the flashlight button) simultaneously until both buttons stop blinking						
Place the PAPR correctly, adjusting the straps						
Check that there was no hair or cap impeding the seal						
Turn on the PAPR by holding the power button for 3 seconds						
Take a few deep breaths to check that the positive pressure inside the PAPR was calibrated						
Check if the left light is on and not blinking						
Adjust the speed of the ventilator (1 from 4)						
Put the surgical cap						
Put the gloves						
Total (for each task)						
**2. Arrive at bedside e get patient status (expected time: 5 min)**						
**Start time:** ____________						

1.19 Script of debriefing (R4)

FORMS NAME	R4 – script of debriefing		
CONTENT	Questions for debriefing		
SUTDY SITE	☐Brazil ☐ Italy ☐ USA		
DATE	DAY	MONTH	☐ 2021
NAME OF THE PARTICIPANT	FIRST AND MIDDLE NAME	LAST NAME	
PARTICIPANT ID			
NAME OF THE RESEARCH ASSISTANT	FIRST NAME	LAST NAME	

What PPE did the participant use in the session just completed?	☐ Light PAPR<br>☐ Traditional PAPR
The current time you are starting this Survey is:	________________

Debriefing

This debriefing aims at clarifying participants' concerns while collecting extra information with respect to their feelings, impressions, and opinions on light PAPR in comparison to other respirators they normally use.

Instruction: Participants should be observed during the simulation so that light PAPR adjustments and communication failure can be noticed. Right after PPE doffing, participants should proceed to a private room to answer the following questions:

1. What is your general impression about performing the simulation with this PPE?
2. Do you have any concerns about PAPR donning and doffing?
3. Was there any difficulty to move yourself or perceive other people and/or objects around you?
4. I noticed you (insert, if necessary, observation issues such as adjustments, communication problems, errors while performing tasks). Would you like to comment on that?
5. In general, how comfortable is the light PAPR if compared to the N95 respirator and Faceshield? or PAPR

- ☐ Much more comfortable
- ☐ More comfortable
- ☐ As comfortable as either PFF2 mask and Faceshield
- ☐ Less comfortable
- ☐ Much less comfortable

6. Check the discomfort areas in your face below

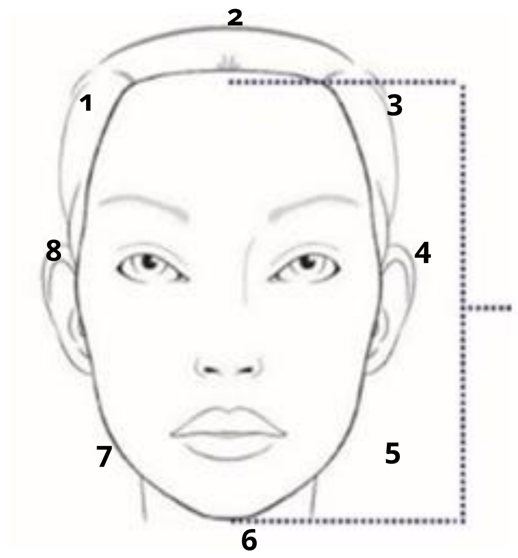

7. Specific Use Errors related to PAPR components (please circle the specific components that required attention during don/use/doff)

1.20 Field Study – Data Collect Form (R5)

Description of patient load

Total number of patients: _____________	Patient load – Participant A: __________rooms
Patients with COVID: _____________	Patient load – Participant B: __________ rooms
Category of ward/ICU: ☐ Covid ward ☐ Non-Covid ward	

What PPE did the participant use in this session?	☐ Light PAPR<br>☐ N95 + Face shield
The current time you are starting this Survey is:	_________________

General errors – Donning PPE1

Task	Errors (codes)	Observations

General errors – During the use PPE1

Task	Performed (yes or not)	Errors (codes)	Observations
☐ Aspiration/suction			
☐ Assessment patient status (clinical exam)			
☐ Assessment physiologic signs (2/2h)			
☐ Body hygiene			
☐ Boluses			
☐ Catheter care			
☐ Changing positioning			
☐ Clinical computer records			
☐ Dialysis			
☐ Discussion of clinical cases (medical team			
☐ Diuresis measurement			
☐ Dry body hygiene			
☐ ECMO			
☐ Exchange of orotracheal tube fixation			
☐ Facial Trichotomy			
☐ Injection preparation			
☐ Intubation			
☐ IV Infusions			
☐ Mechanical ventilation change parameters			
☐ Oral hygiene			
☐ Proning			
☐ Tracheotomy care			
☐ Wound care			
☐ ___________________			
☐ ___________________			
☐ ___________________			
☐ ___________________			
☐ ___________________			

General errors – Doffing PPE1

Task	Errors (codes)	Observations

Break time and change of PPE

The current time you are finishing this surve is:	___________________

Duration of break time: _______________min	Observations:

What PPE did the participant use in this session?	☐ Light PAPR<br>☐ PAPR<br>☐ N95 + Face shield
The current time you are starting this Survey is:	____________________

General errors – Donning PPE2

Task	Errors (codes)	Observations

General errors – During the use PPE2

Task	Performed (yes or not)	Errors (codes)	Observations
☐ Aspiration/suction			
☐ Assessment patient status (clinical exam)			
☐ Assessment physiologic signs (2/2h)			
☐ Body hygiene			
☐ Boluses			
☐ Catheter care			
☐ Changing positioning			
☐ Clinical computer records			
☐ Dialysis			
☐ Discussion of clinical cases (medical team			
☐ Diuresis measurement			
☐ Dry body hygiene			
☐ ECMO			
☐ Exchange of orotracheal tube fixation			
☐ Facial Trichotomy			
☐ Injection preparation			
☐ Intubation			
☐ IV Infusions			
☐ Mechanical ventilation change parameters			
☐ Oral hygiene			
☐ Proning			

☐ Tracheotomy care
☐ Wound care
☐ _______________
☐ _______________
☐ _______________
☐ _______________
☐ _______________

General errors – Doffing PPE2

Task	Errors (codes)	Observations

The current time you are finishing this Survey is:	_______________

Error Codes

1	Touching the face/mask/faceshield/PAPR in the patient care environment
2	Stumbling or falling
3	Errors in the urinary measurement or discard of urine
4	Touching chin at chest/shoulder
5	Place the Faceshield/PAPR (contaminated or clean) in the wrong area (clean or contaminated)
6	Incorrect or omission of decontamination of the Faceshield/PAPR
7	Incorrect adjustment of the mask/PAPR
8	Lift the visor or remove faceshield/PAPR in the patient care environment
9	Incorrect removal of mask/PAPR
10	Inadequate use of stethoscope (e.g.: put it against the chest, put over the cap in the ear; touching the stethoscope in the mask/faceshield/tiki)
11	Inadequate positioning of the blower and filters of PAPR
12	Forgetting change/use/remove of gloves when indicated
13	Forgetting change/remove gowns or aprons when indicated
14	Errors in the hand hygiene technique
15	Forgetting performing hand hygiene when indicated
16	Dropping objects, devices

17	Difficulty in visualizing/record site to be punctured, vital signs measured by the monitor; setting mechanical ventilator parameters
18	Loss/dislogement/disconection of devices (central venous catheter; nasoenteral catheter; peripheral catheter)
19	Brush up against other HW or equipment or furniture
20	Incorrect order or technique of donning or doffing PPE (gowns, caps, etc)
21	Error or omission of disinfection of surfaces in the patient care environment
22	Touch the mobile/phone in the faceshield/mask/PAPR

1.21 Simulation check list (S4)

FORM NAME	S4 – Simulation Checklist		
SUBJECT	Description of step-by-step simulation preparation and organization		
RESEARCH LOCATION	☐Brazil ☐ Italy ☐USA		
DATE	DAY	MONTH	☐ 2021
PARTICIPANT NAME	FIRST NAME	LAST NAME	
PARTICIPANT ID			
RESEARCH ASSISTANT NAME	FIRST NAME	LAST NAME	
FACILITATOR NAME 1	FIRST NAME	LAST NAME	
FACILITATOR NAME 2	FIRST NAME	LAST NAME	

Pre-study (previous day)

☐ Welcome the participant, guide them and ask them to sign the informed consent form

☐ Request the participant to fill out the sociodemographic form

☐ Instruct the participant on how to use the watch and ask them to sign the equipment's usage commitment agreement. Hand them one of the copies and file the other one.

☐ Hand them over a copy of the PAPR quick guide (S1), ensure that they have received a digital copy of the manual, and make the PAPR assembly and donning/doffing instruction video available. Request the participant to sign the assembly and donning/doffing instructions form

☐ Perform the Fit test (3M).

1. Simulation

A – Coordinator Role

- Participants will have already placed their belongings in the closets on the lower floor.

- Explain the following points:

☐ The simulation aims to evaluate the usability and comfort of the LPAPR mask, and not the technical performance of the participant;

☐ Participants can stop the simulation at any time if they so wish, for whatever reason;

☐ If they need any kind of support, facilitators can help;

☐ The scene will be played twice: once with the LPAPR safety mask, and then with N95 + Face shield (inform the randomic order that was previously set);

☐ The coordinator and facilitators will present and discuss the case with the participants. There will be two observers in the scene bearing clipboards to take notes. Introduce everyone to the participants;

☐ Participants should perform procedures as in everyday life, obeying biosecurity and infection control recommendations;

☐ There will be a one-hour break between scenes. During this break, participants may go out and eat;

☐ Present the scenario (donning, doffing, scrubbing, nursing post, patient box) and the manikin to the participants. It's important to remark:

Cardiac and pulmonary auscultation points are indicated on the manikin;

Impossibility of flexing the upper limbs;

Puncture on the left arm of the manikin;

Possibility of not returning blood in the puncture;

Checking vital signs only on the front part of the manikin's body;

Parameter that can be viewed on the monitor: heart rate and rhythm, other parameters – respiratory rate – should be evaluated with the help of the watch and stethoscope.

☐ The whole simulation will be recorded, but the images will not be exposed; they serve only to record the data.

Carry on to the LPAPR masks for first contact

☐ Guide them to say what they are doing (Think Aloud). Observers will be taking notes;

☐ Any doubts about the assembly of the mask can be solved by the facilitators if the participant presents distress, discomfort or something of the kind;

☐ Reassure them about the possibility of breaking the mask; no problem regarding this;

☐ Guide them to put on the suit to start the simulation.

With facilitators and donned observers, lead participants to the donning

☐ Record the schedules of the activities in the simulation

☐ Present the case

☐ Enter for prone positioning

☐ End scene

☐ Thank them and ask them to wait an hour for the next session, or that one of the participants follow Luciana to conduct an interview, while the other should wait for about half an hour

Guide participants to pause for an hour, explaining that the scene will be repeated, but with another respirator, and that they can leave or eat during the break. Say that there is a snack prepared for them.

For all participants and facilitators

☐ Contain your anxiety, anger, frustration, etc., if they arise;

☐ Make sure the watch is up and running;

☐ Make sure the questionnaires are printed on the clipboard in the correct order;

☐ Provide guidance on the objectives of the simulation and clarify that the participant is not being evaluated regarding their technical performance and that you can make comments at the end, during the debriefing;

☐ Manage the questions of the participants so as not to answer them, but to return the question to them. Do not respond the questions immediately;

☐ Do not answer questions directly. Return questions with other questions (without directing);

☐ Use appropriate language (do not laugh, do not use aggressive tone, encourage, thank). Beware of agreement and disagreement tones;

☐ For exploratory testing:

Remind them about the “Think aloud”;

- Be open to adjust the script on time;
- Keep the session on time, but allow for deeper exploration when an opportunity arises;
- Remember to make the participant comfortable and explore the mental model: what are they thinking? What are they trying to do?

☐ Make sure the sound and image equipment is turned on and working properly;

☐ Make sure that the simulator is set according to the agreed parameters (pulmonary, cardiac auscultation and vital signs)

☐ Make sure that the multiparameter monitor, mechanical fan and Infusion pumps are working and programmed properly);

☐ Make sure that the records of the activities of the doctor and nurse have been printed, as well as the result of the gasometry examination;

☐ Ensure that the PPE sequence for the activities is in accordance with the randomization previously performed;

☐ Check materials (see map with room layout)

Environment 1– Observation area

ok	Material (Sound and image)	Amount
	SSD Notebook with installed camera programs	01
	Soundboard	01
	Handheld microphone	01
	Headset	01
	Table	01
	Chair (Rodrigo and other technician)	02
	Tablet	01
	Nobreak	01
	Tape	01
	Regular mask (box)	01
	Cables and adapters	various
	Electric power extension	03

Environment 2 – Donning

ok	Material (general)	Amount
	Plate "DONNING" (printing on A4)	01
	Plastic box with participant name and mask inside	02
	LPAPR mask's step-by-step donning explanatory banner	01
	N95 and Faceshield's step-by-step donning banner (See model HC)	01
	Tripod for banner	01
	Alcohol pump bottle for hand sanitizing	01
	Procedure glove box (sizes S, M and L)	1 of each
	Apron	02
	Cap	02
	N95 (HC and 3M option)	02 of each
	Sachet for putting on the mask	02
	Face shield	02
	Mirror	01
	Screen	01
	Regular mask (box)	01

	Trash for disposal of the surgical mask	01
	Colored tape to delimit the donning counter	01

Environment 3 – Clinical assistance area

ok	Material (Sound and image)	Amount
	Router	01
	Soundboard	01
	Cameras installed according to project (IP, PTZ and/or GoPro – borrow from CELAB)	01
	Omnidirectional microphone (installed on the ceiling or on top of a surface – Set with Rodrigo)	01
	Tripod for camera	01
ok	Materials and equipment (room)	Amount
	Multiparameter monitor	01
	Mechanical blower (HC)	01
	"Fake" gas ruler with suction bottle*	01
	Drip support	01
	Infusion pumps	02
	Fake sedation solution prepared with saline solution 250ml	02
	Infusion pump equipment	02
	Patient nameplate (HC model)	01
	Prone time nameplate)	01
	Trash can	02
	Liquid for urine simulation	01
	Note-taking desk	01
	Drawing boards with prints (see list of prints)	02
	Clipboard with patient examinations	01
	Clipboard with observation script	02
	Pen	06
	Hamper*	01
ok	Clinical materials and equipment (bed)	Amount
	Stretcher with manikin with connected devices (endotracheal tube fixed with strings, trachcare, nasoenteral tube, CVC, electrodes, multiparametric monitoring, foley catheter and collecting bag, diaper), with identification bracelet and hydrocolloid plates fixed in the places determined by the HC prone protocol and indication of auscultation site	01
	Sheets (2 on the stretcher and 1 extra for the prone position)	03
	Rolls of cushion sheets	02
	Head cushion (prone position)	01
	Latex extension and suction tube*	01
	Diet and diet equipment*	01
ok	Clinical materials and equipment (counter)	Amount
	Procedure glove box (sizes S, M and L)	1 of each
	Alcohol pump bottle for hand sanitizing	01
	Almotolia alcohol 70%*	01
	Stethoscope	02
	Stainless steel or plastic tray*	01

	Container with non-sterile gauze	01
	Container with cotton	01
	Graduated urine collector container*	01
	Syringe 10ml	05
	Needle 40x12	05
	10ml ampoule of saline	05
	Peripheral venous catheter n18	05
	Peripheral venous catheter n20	05
	Tourniquet*	01
	Peripheral venous catheter fixator	05
	3-way tap	05
	Micropore roll	01
	Disposal for sharps	01
	Extra electrodes	10
	Graduated urine collector container*	01
	Disinfectant and decontamination cloth	01
	Nursing trolley*	02
	Calculator	01
	Apron	05

Environment 4 – Doffing environment

ok	Material (general)	Amount
	Plate "DOFFING" (printing on A4)	01
	Plate "CONTAMINATED AREA"	01
	Plate "clean area"	01
	Tape for separation of areas	01
	Screen	01
	Table/part of counter reserved for disposal of contaminated LPAPR mask and faceshield (identified as "contaminated area")	01
	Table/counter part reserved for clean LPAPR mask and faceshield (identified as "clean area")	01
	LPAPR mask's step-by-step doffing explainer banner	01
	N95 and Faceshield's step-by-step doffing explanatory Banner (See HC model)	01
	Tripod for banner	01
	Alcohol pump bottle for hand sanitizing	01
	Trash can*	01
	Disinfectant and decontamination cloth	01

Environment 5 – Debriefing room and qualitative interview

ok	Material (general)	Amount
	Clipboard with prints (see list of prints)	01

	Pen	02
	Voice recorder	01
	Computer with internet access	01
	Microphone to be connected to the computer	01

Environment 6 – Bathroom and support area of the participants

ok	Material (general)	Amount
	Packaging with private uniform with participant's name	01
	Lapel microphones	02
	Badge	02
	Hydrographic pen	01
	Hamper with bag (to dispose of worn clothes)	01
	Non-contact thermometer (confirm purpose)	01
	Pulse oximeter (confirm purpose)	01

Environment 7 – Break room

ok	Material (general)	Amount
	Snack kit for participants	2
	Gift for participants	2
	Water glasses	20
	Overhead projector pen	03
	Sellotape	02
	Scissors	02
	Tape	01
	Self adhesive tags	10 big units

* CELAB materials

3) Completion of the simulation

☐ If the task is not completed within the stipulated deadline, close the scenario, as this factor can cause stress among the participants as well as the slow pace in the execution of the tasks can reduce the degree of immersion in the scene.

☐ Refer the participant to debriefing

4) Discrimination of activities (confirm)

Pre-study (previous day)

- HC room (Research Assistants)

☐ Watch guidelines

☐ Delivery of the guide

☐ Request the completion of the Sociodemographic Form

☐ Signature of the Consent form

☐ Fit testing?

☐ Guidance on storing belongings in the closet in the CELAB building

☐ Resolution of other questions

Simulation

Facilitators

☐ Must arrive 30 minutes in advance and don

☐ Check the operation of the "work clock"?

☐ Present the manikin and the room to the participants

☐ Review the simulation scene

☐ Solve any problems, answer questions

*They will be on the scene before the entrance of the participants in the preparation

** Remain on the scene after simulation in the ICU room

Research Assistant 1

☐ Makes sure that the signs with the indication of CELAB are placed in the lobby and in the marquee

☐ Welcomes participants at the marquee

☐ Guides them on storing belongings in the closet of the building

☐ Offers surgical mask and zip bag if participant is wearing fabric mask

☐ Checks the use of the watch

☐ Explain the dynamics of the activity (manikin and scene presentation, PPE1 simulation, NASA and SUS, pause/snack, PPE2 simulation, NASA and SUS, debriefing and qualitative questionnaire)

☐ Solve any questions

☐ Assists in recording and organizing participants during the simulation

☐ Offer the snack

Research Assistant 2

☐ Assists in scenario preparation

☐ Tracks and observes simulations

☐ Acts as the fifth element in prone positioning

☐ Checks participants ' errors with research assistants 2 and 3

☐ Prepares the room for post-simulation questionnaires

Research assistants 3 and 4

☐ Prepares the scenarios

☐ Observe the simulation

☐ Guide facilitators, if any

1.22 Scenery Script (S5)

FORM NAME	S5-Simulation scenario		
SUBJECT	Description of the scenario and procedures to be performed during the simulation		
RESEARCH LOCATION	☒ Brazil ☐ Italy ☐ USA		
DATE	DAY	MONTH	☐ 2021
PARTICIPANT NAME	FIRST NAME	LAST NAME	
Participant ID			
RESEARCH ASSISTANT NAME	FIRST NAME	LAST NAME	

Context: Adult intensive care unit-COVID-19

Distraction: None planned

Goal: Perform the described procedures, using scrubbing, gowning and gloving technique, according to the randomization of the study.

Team: To carry out the study, at least two medical professionals must be called to perform the tasks of the study related to their professional field.

The activities of each simulation team member are described in the table below:

Role	Professional category	Activities
Coordinator	Does not apply (team researcher)	Check equipment operation; start recording. Your interference in the scene should be minimal, only when there are some technical problems or some situation that the facilitators could not control.
Observer 1	Research assistant	Follow the observation script and record the actions of the medical professional or nurse, in the absence of the medical

		professional at the session. They should not perform any interference in the scene.
Observer 2	Research assistant	Follow the observation script and record the actions of the nursing professional. At the moment of positioning the patient in a prone position, The Observer 2 should assist in the mobilization, positioning themselves next to the left lower limb, in front of the nurse (who will be next to the right lower limb), so that they can continue observing this professional.
Facilitator 1	Nurse	Act as a member of the assistance team and dialogue with the research participants, according to the script.
Facilitator 2	Nurse	Act as a member of the assistance team and dialogue with the research participants, according to the script. In the absence of a medical professional in the simulation section, you must act as a doctor in the scene.
Participant 1	Doctor or nurse	Carry out the activities provided on the script, according to the professional category. Study sample subject. In the impossibility of participation of the medical professional, the participants of the research will be two nurses and the facilitator 2 will act as a doctor.
Participant 2	Nurse	Carry out the activities provided on the script, according to the professional category. Study sample subject.

Contextualization of the scenario

(The Observer 1 or Coordinator directs the speech to the two participants, who will be in the scrubbing, gowning nd gloving area. The other members of the simulation team will be in the assistance environment)

In this simulation you are part of the care team responsible for the day shift of this adult ICU, intended for patients with COVID-19. In the proposed scenario, you are starting the work shift at this time; therefore, you should do scrubbing, gowning and gloving in the anteroom, according to the recommendations and guidelines received. Here are some posters with the instructions (point to the banner referring to the PPE that will be evaluated). Inside the room, you will be informed about the patient's clinical situation. The purpose of this study is not to evaluate your technical ability or professional performance, so carry out the activities with peace of mind (see coordinator's checklist).

(The participants perform scrubbing, gowning and gloving in the anteroom, while they are observed by the observers. After finishing the process, the participants enter the assistance unit and the observers fill the observations reports)

(The coordinator will explain where the materials are and that the simulation manikin has some restrictions, such as: the impossibility of flexing the upper limbs; checking vital signs being only possible in the anterior part of the manikin's body; easier points to perform cardiac and pulmonary auscultation; the only parameter that can be visualized on the monitor is the heart rate and rhythm; other parameters – respiratory rate – should be evaluated with the help of the watch and stethoscope).

(Coordinator directs the speech to the team)

Patient Mrs. Julia, 52 years old, with a history of diabetes mellitus and arterial hypertension, was admitted on the previous shift, with a diagnosis of COVID-19 confirmed by RT-PCR. The symptoms started 7 days ago. Due to tachypnea, decreased oxygen saturation, hypoxemia and failure in ventilatory and oxygenation measures previously done, it was decided to perform orotracheal intubation. All procedures, such as central venous catheter insertion, nasoenteral probe and bladder delay catheter, have already been performed.

(Facilitator 1 directs speech to the doctor)

You, Dr _____________ , are responsible for evaluating this patient, including a brief general condition assessment as well as cardiac and pulmonary auscultation. As soon as you do, record your findings in this form, please (Facilitator 1 delivers a clipboard with printed form for registration and after the participant performs the registration. Facilitator 1 shows the auscultation points on the manikin. The noise of vesicular murmurs with bilateral snoring should be auscultated, RR = 28 mov/min and in cardiac auscultation: 2NSR, without wheezing. After this procedure, speech is once again directed to them).

Dr ____________, we also need a record of the present heart rate and heart rythm on the monitor. Here is a sheet for you to make this record. Record here also the current parameters of the mechanical ventilator.

(Facilitator 1 is responsible for monitoring the activity and observing if there is any difficulty both in viewing the signals on the monitor and in recording on paper. The evaluation results should be as follows: HR = 94bpm, heart rate = sinus, MV parameters: assisted controlled pressure, FiO_2 =70%, PEEP = 6; peak pressure = 32mmHg).

(After the completion of the first task, facilitator 1 directs the speech to the doctor)

Dr _____________ , the patient does not yet have satisfactory ventilation and oxygenation parameters, as well as hemoglobin and hematocrit levels below normal benchmarks (show a printed Exam Result).

(Facilitator 1 and the doctor discuss the results of the examinations and then facilitator 1 directs the speech to the doctor again)

Dr _____________, can you tell me the sedation and muscle blocker and infusion rate?

(Facilitator 1 waits for the doctor to look at the infusion pump the drip speed and requests that they verbalize the values reported).

So, Dr _____________ , the infusion of muscle blocker for the patient has been initiated, sedation has been optimized (point to the infusion pump) and parameters of the mechanical ventilator were adjusted. However, it is asynchronous with the ventilator, the pH of the arterial gasometry is 7.21. Will you help me calculate the partial pressure of oxygen / fraction of inspired oxygen ratio (PaO_2/FIO_2)? (facilitator 1 discusses the gasometry result with the participant – see printed result – and waits for him to do the calculations manually or with the aid of a calculator, while the nursing professionals perform the proper procedures. The calculations result is 87 mmHg). Prone positioning had already been considered on the previous shift, let's do it now! I'll inform the nursing staff.

(Facilitator 1 informs the other participants about the procedure. The manikin should already be prepared, with skin protectors applied and cushions ready. Observe the procedure of the prone positioning and if there are any failures in understanding verbal commands during the mobilization steps).

(Concomitantly, facilitator 2 should direct the speech to the nurse).

(In sessions where there are two nurses, two mannequins will be available for each professional to perform the procedures separately. Only the prone positioning will be performed together, with the whole team in a single manikin).

Mx _____________, the nursing care plan for this patient is our shift's responsibility. Therefore, you must conduct the general condition assessment, including cardiac and pulmonary auscultation. Here is a form for you to record your findings.

(The nurse participant will perform auscultation on the manikin and fill the cardiac and pulmonary auscultation report. Facilitator 2 shows the auscultation points on the manikin. It should be auscultated the noise of vesicular murmurs with bilateral snoring, RR = 28 mov/min and in cardiac auscultation: 2NSR, without wheezing; HR = 94bpm). Once the form is completed, facilitator 2 directs the speech to the participant)

Mx _____________ , in addition, considering the abnormal values of hemoglobin and hematocrit, she needs a peripheral venous access to receive two bags of packed blood cells. You may perform the venipuncture while I check if the packed red blood cells are already available. Thus, the patient can receive the hemocomponent transfusion as soon as possible.

(After completing these tasks, facilitator 2 questions the medical professional about the prone positioning procedure)

Dr____________, it seems that Mrs. Julia has a prone positioning prescribed?

(At this point, facilitators should allow the team to communicate for a few moments, to prepare the prone positioning. Then, facilitator 2 directs the speech to the nurse).

Mx _____________ , this patient's urine bag is full. I think it is better to empty it before performing the prone positioning, so we avoid the risk of this collecting bag bursting during the patient's mobilization. I'll get the volumetric flask so you can measure the urine volume.

(Facilitator 2 delivers the graduated vial and provides a form to fill with the discarded urine volume. The volume contained in the bag that must be registered is of 450ml).

(If the nurse does not understand the need to assist in the prone positioning, facilitator 2 should provide some clues).

(Observer 2 should be positioned next to the LLE to observe the nurse during the mobilization procedure).

(The designated task leader – the medical professional or, in the absence of one, a facilitator acting as the doctor – introduces himself to the team and stands in front of the patient's head (manikin). Two professionals are on each side of the patient's torso, and two on each side near the lower limbs, as shown in Figure 1).

Figure 1. Demonstration of the positioning of professionals during pronation.

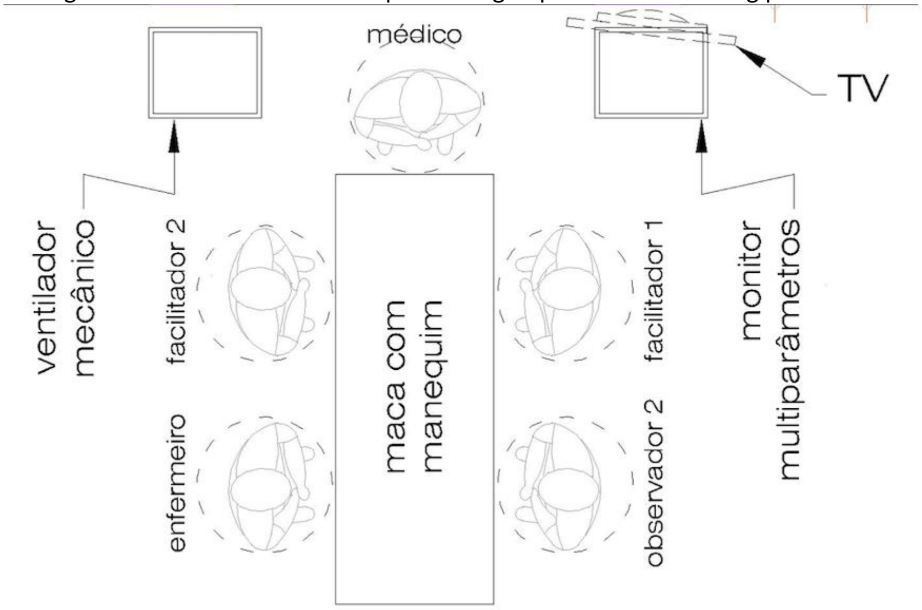

Source: project authors

(If none of the participants mentions the need to check the positions and fixation of the devices, one of the facilitators should question whether the endotracheal tube, the closed tracheal aspiration system, the intravascular and urinary catheters, as well as the nasoenteral probe are all properly fixed and positioned. Similarly, the other facilitator will question whether hydrocolloid dressings are applied to the skin, in the following regions: forehead, chin, chest, shoulders, elbows and knees. At this time, facilitators will act to encourage communication between participants so that observers can identify whether PPE interfere with communication).

(The electrodes should be taken from the anterior part of the chest and new electrodes should be obtained. A clean sheet should be placed so that it covers the patient, forming an "envelope" with the bottom sheet – Figure . The cushions will be positioned on the chest and on the patient's pelvis, as shown in Figure . The team can choose to position the cushions after the patient mobilization).

Figure 2. Formation of the envelope with the sheets.

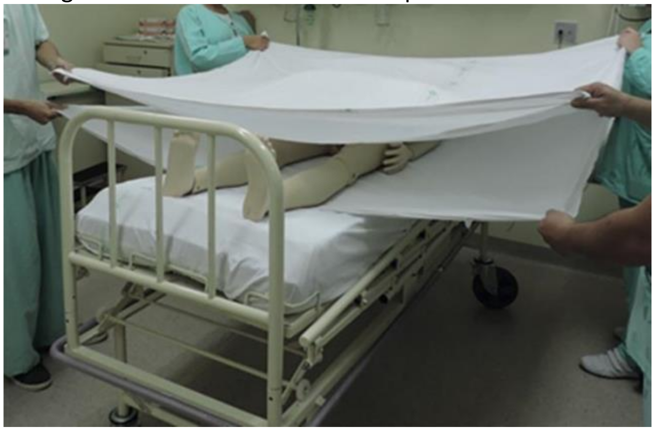

Source: Oliveira et al., 2017

Figure 3. Positioning of cushions and sheets.

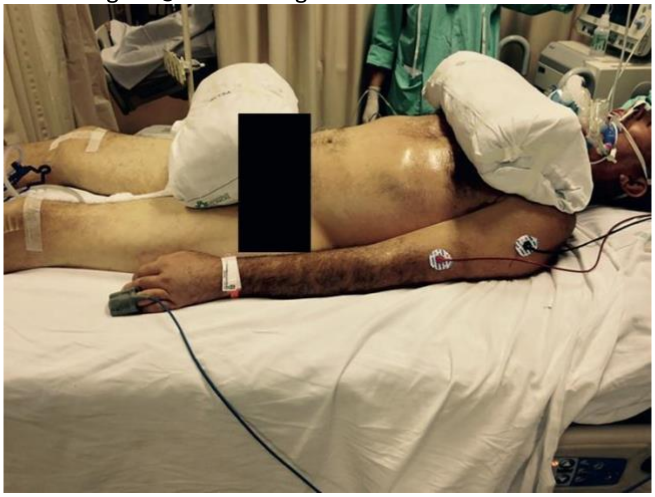

Source: Oliveira et al., 2017

(Next, the task leader must give the command to start moving the patient horizontally on the bed, in the opposite direction of the mechanical ventilator. The hands of the professionals should be positioned on the sides of the sheet envelope, while the professional who is positioned next to the patient's head holds the endotracheal probe and other devices, as shown in Figure).

Figure 4. Positioning of professionals and patient mobilization.

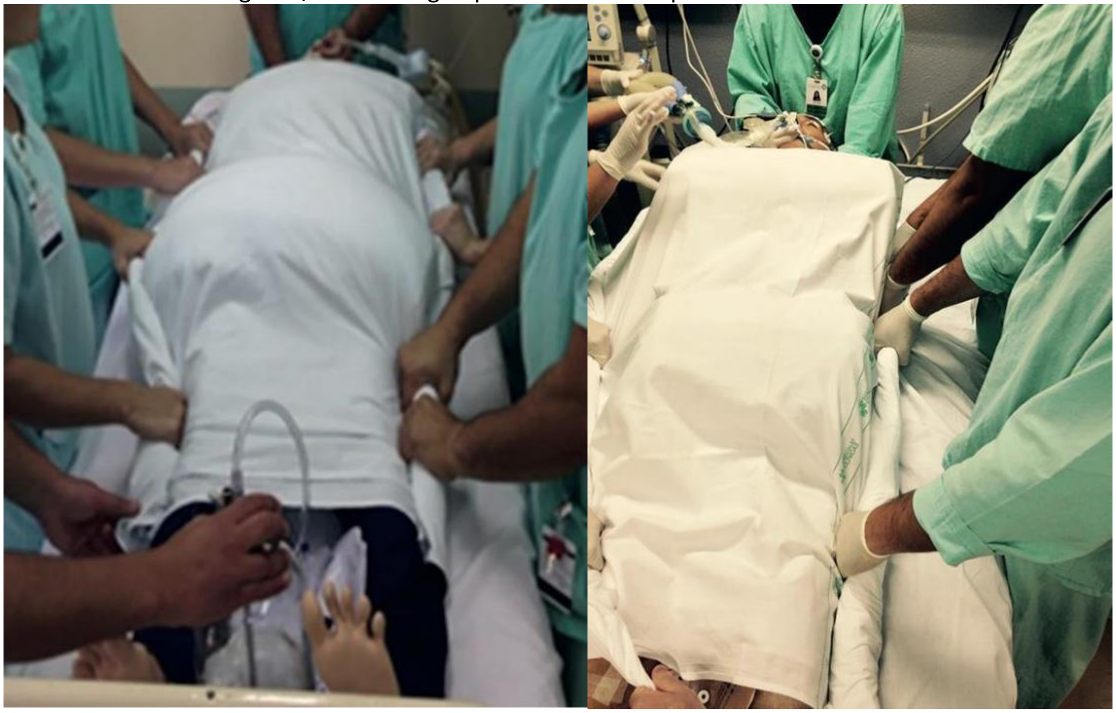

Source: Oliveira et al., 2017

(The task leader gives the command to turn the patient into lateral position. Then, they inform that the professionals must perform the maneuver of changing hands between the team, placing one hand on the left side and another on the right side of the patient, as shown in Figure 5).

Figure.5 Patient mobilization.

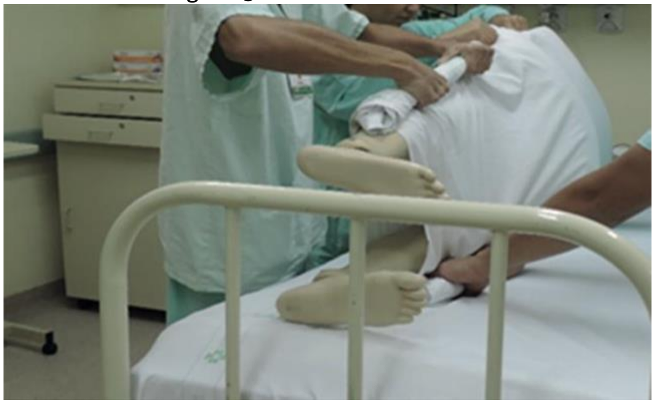

Source: Oliveira et al., 2017

(The task leader gives the command to finish the rotation of the patient, mobilizing him at 180 degrees, as shown in

Figure 6).

Figure 6. Final prone position.

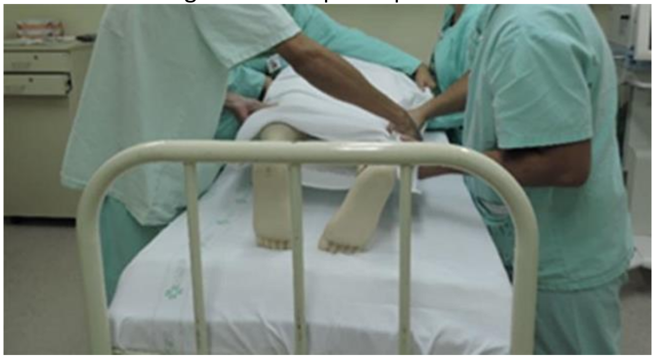

Source: Oliveira et al., 2017

(The task leader gives the commands for checking the devices, positioning the cushions, positioning the patient's upper limbs and cephalic lateralization. Facilitators should make notes if participants do not perform post-prone positioning activities: guarantee that the endotracheal probe, the closed aspiration tracheal system, intravascular catheters, bladder delay catheter and nasoenteral probe are fixed and positioned. New electrodes should be applied to the posterior chest. Vital signs should be checked on the multiparameter monitor in order to assess the clinical stability of the patient. A cushion should be placed under the patient's head. Cushions should be placed under the chest and pelvis to minimize pressure on the abdomen. Team members should position the patient as a swimmer – one arm raised and the head turned towards the raised arm; the other arm is positioned next to the body. as shown in **Error! Reference source not found.**7).

(Once the team performs the positioning of the patient, the facilitator must indicate the need to remove the gloves and sanitize the hands.

Then, facilitator 1 directs the speech to the doctor)

Dr _____________, I think it's prudent for you to make adjustments to the ventilator parameters now so we can perform the alveolar recruitment. Ventilating mode should be volume assisted/controlled, FiO_2 won't need any adjustment (the mechanical ventilator used in this simulation does not allow adjustments to FiO_2). Values should be Tidal volume of 320mL, PEEP of 10 and RR of 24 mov/min.

(Facilitator 2 must direct the speech to the nurse, while the medical professional adjusts the mechanical ventilator parameters).

Mx _____________, in this form we will take note of the prone position start time, so we can program the head lateralization schedule (provide a printed form to the nursing professional and, once it's filled, fix it at the bed foot).

(Facilitator 1 directs speech to the team).

All right, guys! Now we will need to wait for the patient's condition to stabilize, so in an hour a new gasometry should be collected to evaluate if there was an improvement in the parameters.

(Coordinator or observer 1 directs the speech to the team).

We are now ending the assistance-related activities. Now, you can proceed to remove the PPE. Apron and gloves may be removed while in this room. The LPAPR mask or N95 and face shield must be removed in the doffing area (point to the identified area). You must also disassemble the LPAPR safety mask as well as decontaminate the face shield or LPAPR mask according to recommendations. The N95 mask can be disposed of.

(Observe the removal of PPE, decontamination of face shield or LPAPR mask, and disposal of single-use PPE. End the simulation and move on to the completion of instruments).

1.22.1 Reference:

São Paulo Regional Nursing Council Manual of Clinical Simulation for nursing professionals / São Paulo Regional Nursing Council - São Paulo-SP, 2020. Available at: https://portal.coren-sp.gov.br/wp-content/uploads/2020/12/Manual-de-Simulação-Cl%C3%ADnica-para-Profissionais-de-Enfermagem.pdf.

INACSL Standards Committee (2016, December). INACSL standards of best practice: SimulationSM Facilitation. Clinical Simulation in Nursing, 12 (S), S16-S20. http://dx.doi.org/10.1016/j. ecns.2016.09.007.

Kaneko RMU, Lopes MHBM. Realistic health care simulation scenario: what is relevant for its design? Rev ESC sick USP. 2019; 53: e03453. DOI: http:// dx.doi.org/10.1590/S1980-220X2018015703453.

Mitchell DA, Seckel MA. Acute Respiratory Distress Syndrome and Prone Positioning. AACN Advanced Critical Care. 2018.29(4):415-25.

Oliveira VM et al. Safe prone checklist: construction and implementation of a tool for performing the prone maneuver. Rev. bras. have. intensive. 2017;29(2):131-41.

1.23 Arterial blood gas analysis (S6)

GASOMETRY RESULTS

Patient identification data

Name: Julia Vitoria da Silva	DOB: 28/03/1969	Age: 52y
HC registration: 56523	Date of admission:	
Date:	Time of collection: 09:15 am	
Diagnostic hypothesis: SARS-CoV 2 - COVID-19		

ARTERIAL GASOMETRY

Result		Reference range
ph = 7.21		ph = 7.35–7.45
pO2 = 61mmHG		pO2 = 80–100 mmHg
pCO2 = 45mmHG		pCO2 = 35–45 mmHg
HCO3- = 22mmol/L		HCO3- = 22-27 mmol/L
BE = +2		BE = 0 +/- 2mEq/L
O2Sat = 89%		O2Sat = 94-100%

1.24 Lab exams (S7)

LABORATORY TEST RESULTS

Name: Julia Vitoria da Silva	DOB: 28/03/1969	Age: 52y
HC registration: 56523	Date of admission:	
Date of collection:	Time of collection: 09:15 am	
Diagnostic hypothesis: SARS-CoV 2 - COVID-19		

COMPLETE BLOOD COUNT

ERYTHROGRAM

Parameter	Result	Reference range
Erythrocytes	2.70 $x10^6$/mm3	4.20–6.10
Hemoglobin	7.4 g/dL	12.0–18.0
Hematocrit	23.7%	37.0–52.0
MCV	88.0 µm3	80.0–99.0
MCH	27.4 pg	27.0–31.0
MCHC	31.1 g/dL	32.0–36.0
RDW	12.2%	11.5–15.5

LEUKOGRAM

Parameter			Reference range
Leukocytes		18,200 /mm3	4,400 - 10,500
	%	$x10^3$/mm3	
Cells Blasts		0.00	-
Promyelocytes	0.0	0.00	-
Myelocytes	0.0	0.00	-
Metamyelocytes	0.0	0.00	-
Rods	5.0	0.91	-
Segmented	84.0	15.29	48.0–71.5%
Eosinophils	0.0	0.00	0.0–5.0%
Basophils	0.0	0.00	0.0–3.0%
Lymphocytes	3.0	0.55	19.0–50.0%
Lymph. Atypical	0.0	0.00	-
Monocytes	8.0	1.46	2.0–12.0%

Plasmocytes	0.0	0.00	-
Platelets	365 x10³/mm3		130–400 x10³/mm3

COAGULOGRAM

PROTHROMBIN TIME

	Value	Reference range
PT	11.0s	
Standard PT	11.5s	
PA	100%	
INR	1.00	<1.20
aPTT	24.6s	
Standard aPTT	29.1s	
RATIO	0.85	<1.25

BIOCHEMISTRY

Test	Value	Reference range
Calcium	7.9 mg/dL	8.6-10.3 mg/dL
Magnesium	2.1 mg/dL	1.9-2.7 mg/dL
C-Reactive Protein	42.1 mg/L	Mild inflammation: 10-50 mg/l
		Moderate inflammation: 50-100 mg/l
		Severe inflammation: >100 mg/L
Potassium	4.0 mol/L	3.5–5.1 mol/L
Sodium	147.0 mmol/L	136-145 mmol/L
Urea	86.1 mg/dL	17.0–43.0 mg/dL
Creatinine	1.52 mg/dL	Males: 0.70-1.30 mg/dL
		Females: 0.60-1.20 mg/dL
GPT/ALT	59.1 U/L	Less than 52 U/L
GOT	39 U/L	<40U/L

1.25 Nurse assessment (S8)

EVALUATION RECORD - NURSE

Which PPE is the professional wearing in this session?	☐ LPAPR Safety Mask<br>☐ N95 + Face shield
What is the start time for this activity?	___________________

Patient identification data

Name: Julia Vitoria da Silva	DOB: 28/03/1969	Age: 52
HC registration: 56523	Date of admission: 30/04/2021	
Date:	Time:	
Diagnostic Hypothesis:		

The record below the findings obtained from your evaluation, as well as the procedures carried out:

Pulmonary auscultation	
Right Hemithorax:<br>☐ Missing/diminished sounds<br>☐ Vesicular murmurs<br>☐ Snoring<br>☐ Fine Rales<br>☐ Coarse Rales<br>☐ Pings	Left Hemithorax:<br>☐ Missing/diminished sounds<br>☐ Vesicular murmurs<br>☐ Snoring<br>☐ Fine Rales<br>☐ Coarse Rales<br>☐ Pings
Respiratory rate: _ _ _ _ _ _ mov / min	

Cardiac auscultation
☐ 2 BNF, rhythmic, no audible murmurs<br>☐ 2 BNF, rhythmic, with audible murmurs<br>☐ 2 BNF, arrhythmic, no audible murmurs<br>☐ 2 BNF, arrhythmic, no audible murmurs<br>☐ Others: ____
Heart rate: _ _ _ _ _ _ bpm

Peripheral venous puncture

Site of peripheral venous puncture:
Device gauge:

Measurement of urinary flow

Urine Volume (mL):
Characteristics of urine (ex: staining):

Prone position

Prone position time:	Prediction of conclusion:
Cephalic lateralization:<br>☐ Right<br>☐ Left	Upper limbs (indicate flexed member)<br>☐ Left Upper Limb<br>☐ Right Upper Limb

Participant name:	

1.26 Physican assessment (S9)

EVALUATION RECORD – PHYSICIAN

Which PPE is the professional wearing in this session?	☐ LPAPR Safety Mask<br>☐ N95 + Face shield
What is the start time for this activity?	__________________

Patient identification data (mock sample)

Name: Julia Vitoria da Silva	DOB: 28/03/1969	Age: 52
HC registration: 56523	Date of admission: 30/04/2021	
Date:	Time:	
Diagnostic Hypothesis:		

Report below the findings obtained during the evaluation, as well as the physiological parameters verified on the monitor:

General Condition Assessment

☐ Flushed<br>☐ Pale<br>☐ Jaundice<br>☐ Cyanotic<br>☐ Other: __________________	☐ Hydrated<br>☐ Dehydrated ___________
☐ Alert and oriented<br>☐ Somnolent<br>☐ Stuporous<br>☐ Comatose<br>☐ Sedated	☐ Good condition<br>☐ Fair condition<br>☐ Serious condition

Respiratory pattern	
☐ Breathing air<br>☐ Oxygen therapy<br>Type of ventilatory assistance: _____________________________________<br>Saturation of O2: ________________ Respiratory rate: ___________ mov/min	
Pulmonary auscultation	
Right Hemithorax:<br>☐ Missing / diminished sounds<br>☐ Vesicular murmurs<br>☐ Snoring<br>☐ Fine Rales<br>☐ Coarse Rales<br>☐ Pings	Left Hemithorax:<br>☐ Missing / diminished sounds<br>☐ Vesicular murmurs<br>☐ Snoring<br>☐ Fine Rales<br>☐ Coarse Rales<br>☐ Pings
Cardiovascular pattern	
Heart rate: _ _ _ _ _ _ bpm	Blood pressure: ____________mmHg
Heart rate (observed on multi-parameter monitor)<br>☐ Sinus	

☐ Irregular. Specify: ____
☐ 2 BNF, rhythmic, no audible murmurs<br>☐ 2 BNF, rhythmic, with audible murmurs<br>☐ 2 BNF, arrhythmic, no audible murmurs<br>☐ 2 BNF, arrhythmic, no audible murmurs<br>☐ Other: ____

Report below the parameters set on the mechanical ventilator:

Mechanical ventilation

Mechanical ventilation mode: _______________________________________	
FiO2:	PEEP:
Plateau pressure:	Tidal volume:
Respiratory rate:	ETT Depth (cm):

Participant name:	

1.27 Qualitative interview (S11)

QUALITATIVE INTERVIEW

1. Do you perceive any advantage or disadvantage when using the light PAPR compared to other facial protection that you we have tested or are used to?
2. What was your experience when using of the light PAPR compared to the other facial protection that you are used to?
3. What kinds of changes or alterations could you make the light PAPR more effective for use in your setting?
4. How complicated is the use of light PAPR in comparison to the other PPE you used in this study?
5. Which support material (posters, marketing materials, standard operation procedures, etc) did you find to be most effective for understanding and application of light PAPR use?
6. What extra barriers might patients and their families face if a health worker uses the light PAPR?
7. What kind of additional infrastructure or process change will be needed for implementation of the light PAPR in care for patients with respiratory transmitted diseases?
8. Compared to other PPE do you think there is a strong need to implement light PAPRs for routine work to care for patients with respiratory transmitted diseases?
9. How does implementation of the light PAPR or other PPE fit with existing work process and practices in your setting? What barriers do you think might limit implementation?
10. How prepared are you to use a PAPR in your routine care of patients? Why?
11. Who do you think are the key individuals to get on board to implement a new PPE in the routine care?
12. Make any comments you think are relevant to the use of light PAPR or the other PPE you have tested in this study.

1.28 Clinical tasks across site (N95 respirator and traditional PAPR)

Sao Palo	Bologna
1 - Donning N95 and Faceshield	1 - Donning 3M Respirator
Perform Hand hygiene	Perform Hand hygiene
Donning the Gown	Check if the battery is charged
Respirator: Prestretch top and bottom straps before placing respirator on the face	Combine the battery to the motor blower
Cup the respirator in your hand, position the respirator under your chin; Pull the bottom strap over your head and position it around the neck below the ears; Adjust the metal nosepiece	Ensure there was no visible damage or debris on the PAPR
To check the respirator-to-face seal, place both hands completely over the respirator and exhale sharply.	Turn on the motor blower
Place the faceshield and adjust it to a comfortable position	Connect/Check the airflow indicator and wait for the airflow to stabilize
Put the surgical cap	Install the breathing tube
Put the gloves	Wear the PAPR belt
TOTAL NUMBER OF ACTIVITIES: 8	Donning the gown ensuring that the tube does not interfere with it
	Put the surgical cap
	Connect the free extremity of the tube to the helmet
	Wear the PAPR helmet
	Put the gloves
	TOTAL NUMBER OF ACTIVITIES: 13
2. Listen to lungs-heart	2. Installation of the Nasogastric Feeding Tube
Hand hygiene	Hand hygiene
Listen to lungs and heart	Select the material for the Nasogastric Feeding Tube
Perform stethoscope disinfection	Humidify the tube
Hand hygiene	Placement of the tube
Register the patient assessment	Check the stagnation
TOTAL NUMBER OF ACTIVITIES: 5	Hand hygiene
	Register the practice
	TOTAL NUMBER OF ACTIVITIES: 7
3. Procedure accomplishment - Start IV	3. Procedure accomplishment - Start IV
Hand hygiene	Hand hygiene
Perform tray-bench disinfection	Perform tray-bench disinfection
Select necessary material for the procedure	Select necessary material for the procedure
Prepare system for vein aseptically salinization (syringe filled with 0.9% saline)	Prepare system for vein aseptically salinization (syringe filled with 0.9% saline)
Hand hygiene	Hand hygiene
Introduce yourself and explain the procedure to patient	Introduce yourself and explain the procedure to patient
Put on gloves	Put on gloves
Select puncture site using the tourniquet	Select puncture site using the tourniquet
Perform skin disinfection via friction movement, waiting for product to naturally dry	Perform skin disinfection via friction movement, waiting for product to naturally dry
Insert venous catheter by safely moving	Insert venous catheter by safely moving
After visualizing blood return, completely insert catheter extension and remove the needle	After visualizing blood return, completely insert catheter extension and remove the needle
Release the tourniquet	Release the tourniquet
Install stopcock while stabilizing catheter with one hand	Install stopcock while stabilizing catheter with one hand
Slowly wash the joint with saline	Slowly wash the joint with saline
Hold catheter in place with dressing	Hold catheter in place with dressing

Register the date of venous puncture and size of catheter peripheral venous access	Register the date of venous puncture and size of catheter peripheral venous access
Dispose of a mandrel in a sharps disposal box	Dispose of a mandrel in a sharps disposal box
Identify catheter with its number, puncture date and professional responsible for procedure	Identify catheter with its number, puncture date and professional responsible for procedure
Porperly discard sharps	Porperly discard sharps
Remove gloves	Remove gloves
Organize the unit	Organize the unit
Hand Hygiene	Hand Hygiene
TOTAL NUMBER OF ACTIVITIES: 22	TOTAL NUMBER OF ACTIVITIES: 22

4. Read urine volume in bag (3) (expected time: 5 min)	4. Read urine volume in bag (3) (expected time: 5min)
Hand hygiene	Hand hygiene
Separate graduated flask to measure diuresis volume	Read the urine volume in the graduated bag
Clamp urine bag	Empty the urine volume in a flask
Measure urine drainage in a graduated flask	Remove gloves and dispose of them in a trash bin
Remove gloves and dispose of them in a trash bin	Hand hygiene
Hand hygiene	Register the measured diuresis volume in a specific form
Register the measured diuresis volume in a specific form	TOTAL NUMBER OF ACTIVITIES: 6
TOTAL NUMBER OF ACTIVITIES: 7	

5. Prone patient (4,5) (expected time: 20 min)	5. Prone patient (4,5) (expected time: 20 min)
Hand Hygiene	Hand Hygiene
The leader team is designated, introduces himself to the team and stands in front of patiet head	The leader team is designated, introduces himself to the team and stands in front of patiet head
Two professionals stand on each side of the patient's trunk and two on each side next to the legs.	Two professionals stand on each side of the patient's trunk and two on each side next to the legs.
Ensure that endotracheal tube, the closed aspiration tracheal system, intravascular and urinary catheters, and gastric tubes are secured	Ensure that endotracheal tube, the closed aspiration tracheal system, intravascular and urinary catheters, and gastric tubes are secured
Check that the hidrocolloid dressings are placed over the following areas: forehead, chin, chest, shoulders, elbows and knees.	Check that the hidrocolloid dressings are placed over the following areas: forehead, chin, chest, shoulders, elbows and knees. + Add the protective glasses
Remove the electrodes from the anterior chest	Remove the electrodes from the anterior chest
Place a new linen over the patient, forming an "envelope"	Place a new linen over the patient, forming an "envelope"
Provide cushions to support the face, chest, pelvis, wrist and anterior leg region. They can be positioned before or after move the patient	Join and wrap the top and bottom sheet as closely as possible to the patient's body
Join and wrap the top and bottom sheet as closely as possible to the patient's body	Move the patient horizontally away from the ventilator
Move the patient horizontally away from the ventilator	Place the patient's hand on the rotating side under the buttock
Place the patient's hand on the rotating side under the buttock	Move the patient into the final complete prone position (horizontal at 180 degrees)
Move the patient into the final complete prone position (horizontal at 180 degrees)	Provide cushions to support the face, chest, pelvis, wrist and anterior leg region. They can be positioned before or after move the patient
Place new electrodes on the back	Place new electrodes on the back
Place the transverse rolls under the pelvis and the chest	Place the transverse rolls under the pelvis and the chest
Place the face turned to the right or the left side	Provide cushion for face with the hole
Place the arms in swimming "crawl" position	Place the face inside the cushion
Provide cushion for face	Place the arms in swimming "crawl" position
Ensure that endotracheal tube, the closed aspiration tracheal system, intravascular and urinary catheters, and gastric tubes are secured	Ensure that endotracheal tube, the closed aspiration tracheal system, intravascular and urinary catheters, and gastric tubes are secured
Check the vitals signs on physiological monitor.	Check the vitals signs on physiological monitor.
Register the time of the beginning of prone positioning.	Register the time of the beginning of prone positioning.

Hand hygiene	Hand hygiene
TOTAL NUMBER OF ACTIVITIES: 21	TOTAL NUMBER OF ACTIVITIES: 21
6- Doffing N95 + Faceshield	6- Doffing 3M respirator
Inside room - correct sequence	Inside room - correct sequence
Remove gloves	Remove gloves
Hand hygiene	Hand hygiene
Remove the Gown	Remove the Gown
Hand hygiene	Hand hygiene
Outside room / antechamber - Correct sequence	Outside room / antechamber - Correct sequence
Remove the surgical cap	Remove the belt
Remove the face shield	Remove the helmet
Decontaminate the face shield, cleaning with a wipe and disinfectant the outside firstly	Remove the surgical cap
With a new wipe, decontaminate the inside of the Faceshield.	Decontaminate the helmet, the tube and the belt, cleaning with a wipe and disinfectant the outside firstly
Place the faceshield on a cleaned surface.	Place the PAPR's component in a clean box
Cup respirator in hand to maintain position on face and pull top strap over head to remove respirator	Perform Hand hygiene
Disposal the respirator	TOTAL NUMBER OF ACTIVITIES: 10
Perform Hand hygiene	
TOTAL NUMBER OF ACTIVITIES: 12	

1.29 Clinical tasks across site (L-PAPR)

SAN PAOLO	BOLOGNA
1 - Donning LPAPR Mask	1 - Donning LPAPR Mask
Perform Hand hygiene	Perform Hand hygiene
Donning the gown	Donning the gown
Be careful with the fragile charger connector	Be careful with the fragile charger connector
Ensure there was no visible damage or debris on the PAPR	Ensure there was no visible damage or debris on the PAPR
Check if the PAPR is charged	Check if the PAPR is charged
Position the internal blower, the inhalation filter (white) and exhalation filter (green) correctly	Position the internal blower, the inhalation filter (white) and exhalation filter (green) correctly
Reset the blower, pressing both buttons (on/off and the flashlight button) simultaneously until both buttons stop blinking	Reset the blower, pressing both buttons (on/off and the flashlight button) simultaneously until both buttons stop blinking
Place the PAPR correctly, adjusting the straps	Place the PAPR correctly, adjusting the straps
Check that there was no hair or cap impeding the seal	Check that there was no hair or cap impeding the seal
Turn on the PAPR by holding the power button for 3 seconds	Turn on the PAPR by holding the power button for 3 seconds
Take a few deep breaths to check that the positive pressure inside the PAPR was calibrated	Take a few deep breaths to check that the positive pressure inside the PAPR was calibrated
Check if the left light is on and not blinking	Check if the left light is on and not blinking

Adjust the speed of the ventilator (1 from 4)	Adjust the speed of the ventilator (1 from 4)
Put the surgical cap	Put the surgical cap
Put the gloves	Put the gloves
TOTAL NUMBER OF ACTIVITIES: 15	TOTAL NUMBER OF ACTIVITIES: 15
2. Listen to lungs-heart	2. Installation of the Nasogastric Feeding Tube
Hand hygiene	Hand hygiene
Listen to lungs and heart	Select the material for the Nasogastric Feeding Tube
Perform stethoscope disinfection	Humidify the tube
Hand hygiene	Placement of the tube
Register the patient assessment	Check the stagnation
TOTAL NUMBER OF ACTIVITIES: 5	Hand hygiene
	Register the practice
	TOTAL NUMBER OF ACTIVITIES: 7
3. Procedure accomplishment - Start IV	3. Procedure accomplishment - Start IV
Hand hygiene	Hand hygiene
Perform tray-bench disinfection	Perform tray-bench disinfection
Select necessary material for the procedure	Select necessary material for the procedure
Prepare system for vein aseptically salinization (syringe filled with 0.9% saline)	Prepare system for vein aseptically salinization (syringe filled with 0.9% saline)
Hand hygiene	Hand hygiene
Introduce yourself and explain the procedure to patient	Introduce yourself and explain the procedure to patient
Put on gloves	Put on gloves
Select puncture site using the tourniquet	Select puncture site using the tourniquet
Perform skin disinfection via friction movement, waiting for product to naturally dry	Perform skin disinfection via friction movement, waiting for product to naturally dry
Insert venous catheter by safely moving	Insert venous catheter by safely moving
After visualizing blood return, completely insert catheter extension and remove the needle	After visualizing blood return, completely insert catheter extension and remove the needle
Release the tourniquet	Release the tourniquet
Install stopcock while stabilizing catheter with one hand	Install stopcock while stabilizing catheter with one hand
Slowly wash the joint with saline	Slowly wash the joint with saline
Hold catheter in place with dressing	Hold catheter in place with dressing
Register the date of venous puncture and size of catheter peripheral venous access	Register the date of venous puncture and size of catheter peripheral venous access
Dispose of a mandrel in a sharps disposal box	Dispose of a mandrel in a sharps disposal box
Identify catheter with its number, puncture date and professional responsible for procedure	Identify catheter with its number, puncture date and professional responsible for procedure
discard sharps	discard sharps
Remove gloves	Remove gloves
Organize the unit	Organize the unit
Hand Hygiene	Hand Hygiene
TOTAL NUMBER OF ACTIVITIES: 22	TOTAL NUMBER OF ACTIVITIES: 22
4. Read urine volume in bag (3) (expected time: 5 min)	4. Read urine volume in bag (3) (expected time: 5min)

Hand hygiene	Hand hygiene
Separate graduated flask to measure diuresis volume	Read the urine volume in the graduated bag
Clamp urine bag	Empty the urine volume in a flask
Measure urine drainage in a graduated flask	Remove gloves and dispose of them in a trash bin
Remove gloves and dispose of them in a trash bin	Hand hygiene
Hand hygiene	Register the measured diuresis volume in a specific form
Register the measured diuresis volume in a specific form	TOTAL NUMBER OF ACTIVITIES: 6
TOTAL NUMBER OF ACTIVITIES: 7	
5. Prone patient (4,5) (expected time: 20 min)	5. Prone patient (4,5) (expected time: 20 min)
Hand Hygiene	Hand Hygiene
The leader team is designated, introduces himself to the team and stands in front of patiet head	The leader team is designated, introduces himself to the team and stands in front of patiet head
Two professionals stand on each side of the patient's trunk and two on each side next to the legs.	Two professionals stand on each side of the patient's trunk and two on each side next to the legs.
Ensure that endotracheal tube, the closed aspiration tracheal system, intravascular and urinary catheters, and gastric tubes are secured	Ensure that endotracheal tube, the closed aspiration tracheal system, intravascular and urinary catheters, and gastric tubes are secured
Check that the hydrocolloid dressings are placed over the following areas: forehead, chin, chest, shoulders, elbows and knees.	Check that the hidrocolloid dressings are placed over the following areas: forehead, chin, chest, shoulders, elbows and knees. + Add the protective glasses
Remove the electrodes from the anterior chest	Remove the electrodes from the anterior chest
Place a new linen over the patient, forming an "envelope"	Place a new linen over the patient, forming an "envelope"
Provide cushions to support the face, chest, pelvis, wrist and anterior leg region. They can be positioned before or after move the patient	Join and wrap the top and bottom sheet as closely as possible to the patient's body
Join and wrap the top and bottom sheet as closely as possible to the patient's body	Move the patient horizontally away from the ventilator
Move the patient horizontally away from the ventilator	Place the patient's hand on the rotating side under the buttock
Place the patient's hand on the rotating side under the buttock	Move the patient into the final complete prone position (horizontal at 180 degrees)
Move the patient into the final complete prone position (horizontal at 180 degrees)	Provide cushions to sup,port the face, chest, pelvis, wrist and anterior leg region. Tmovingcan be positioned before or after move the patient
Place new electrodes on the back	Place new electrodes on the back
Place the transverse rolls under the pelvis and the chest	Place the transverse rolls under the pelvis and the chest
Place the face turned to the right or the left side	Provide cushion for face with the hole
Place the arms in swimming "crawl" position	Place the face inside the cushion
Provide cushion for face	Place the arms the in swimming "crawl" position
Ensure that endotracheal tube, the closed aspiration tracheal system, intravascular and urinary catheters, the and gastric tubes are secured	Ensure that endotracheal tube, the closed aspiration tracheal system, intravascular and urinary catheter tubes are secured
Check the vital signs on the monitor.	Check the vital signs on physiological monitor.
Register the time of the beginning of prone positioning.	Register the time of the beginning of prone positioning.
Hand hygiene	Hand hygiene

TOTAL NUMBER OF ACTIVITIES: 21	TOTAL NUMBER OF ACTIVITIES: 21
6 - Doffing and Decontaminate -LPAPR Mask	6 - Doffing and Decontaminate -LPAPR Mask
Inside room - correct sequence	Inside room - correct sequence
Remove gloves	Remove gloves
Hand hygiene	Hand hygiene
Remove the Gown	Remove the Gown
Hand hygiene	Hand hygiene
Outside room / antechamber - Correct sequence	Outside room / antechamber - Correct sequence
Remove the surgical cap	Remove the surgical cap
Remove the mask by pulling on the clips, without touching clothing.	Remove the mask by pulling on the clips, without touching clothing.
Avoid touching the face and the front of the PAPR	Avoid touching the face and the front of the PAPR
Place the PAPR on a designated surface until cleaning and decontamination have been performed	Place the PAPR on a designated surface until cleaning and decontamination have, been performed
To decontaminate the PAPR, first rem,ove the filters	To decontaminate the PAPR, first remove the filters
Remove the screw seal on the left side of the mask.	Remove the screw seal on the left side of the mask.
Remove the blower	Remove the blower
Take a wipe and add a generous amount of disinfectant and clean the PAPR in this sequence: outside, strips, clips,	Take a wipe and add a generous amount of disinfectant and clean the PAPR in this sequence: outside, strips, clips,
With a new wipe with disinfectant, cleanthe the inside of the mask, including the inside of blower and unit filters, without immersing the filters and blower in the disinfectant.	With a new wipe with disinfectant, cleanthe the inside of the mask, including the inside of blower and unit filters, without immersing the filters and blower in the disinfectant.
Place the PAPR in a cleaned plastic box until the next use	Place the PAPR in a cleaned plastic box until the next use
Perform Hand hygiene	Perform Hand hygiene
TOTAL NUMBER OF ACTIVITIES: 15	TOTAL NUMBER OF ACTIVITIES: 15
