# Supplementary material for: Perceived Workload Using Separate (Filtering Facepiece Respirator and Face Shield) and Powered Air-Purifying Respirator and Integrated Lightweight Protective Air-Purifying Respirator: Protocol for an International Multisite Human Factors Randomized Crossover Feasibility Study
Source: JMIR Res Protoc. 2022 Dec 1;11(12):e36549. doi: 10.2196/36549 (PMC9756122; doi:10.2196/36549)
Supplement: Multimedia Appendix 2 [file resprot_v11i12e36549_app2.pptx]

## Slide 1
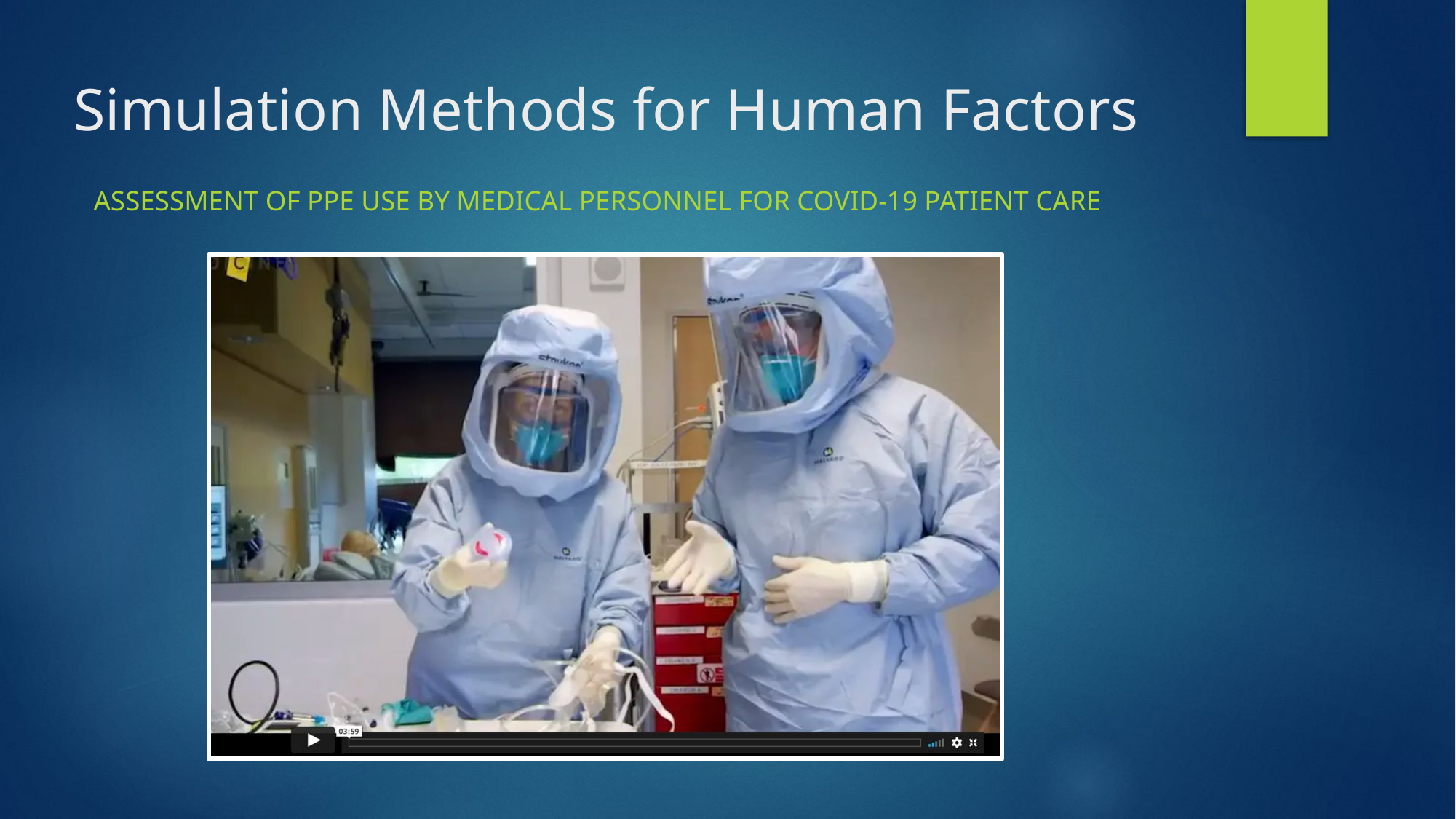

# Simulation Methods for Human Factors
Assessment of PPE use by Medical Personnel for COVID-19 Patient Care

## Slide 2
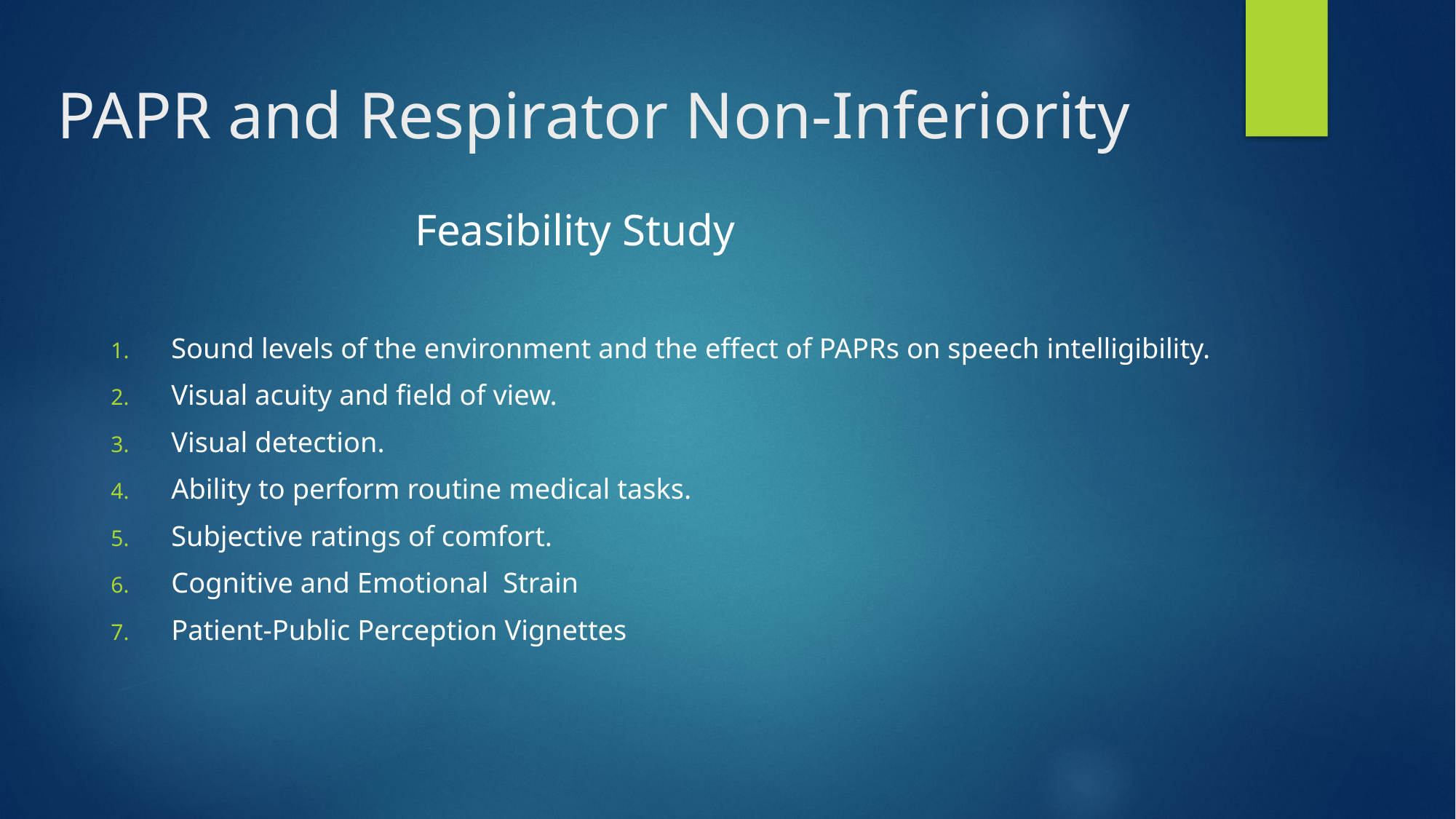

# PAPR and Respirator Non-Inferiority
Feasibility Study
Sound levels of the environment and the effect of PAPRs on speech intelligibility.
Visual acuity and field of view.
Visual detection.
Ability to perform routine medical tasks.
Subjective ratings of comfort.
Cognitive and Emotional Strain
Patient-Public Perception Vignettes

## Slide 3
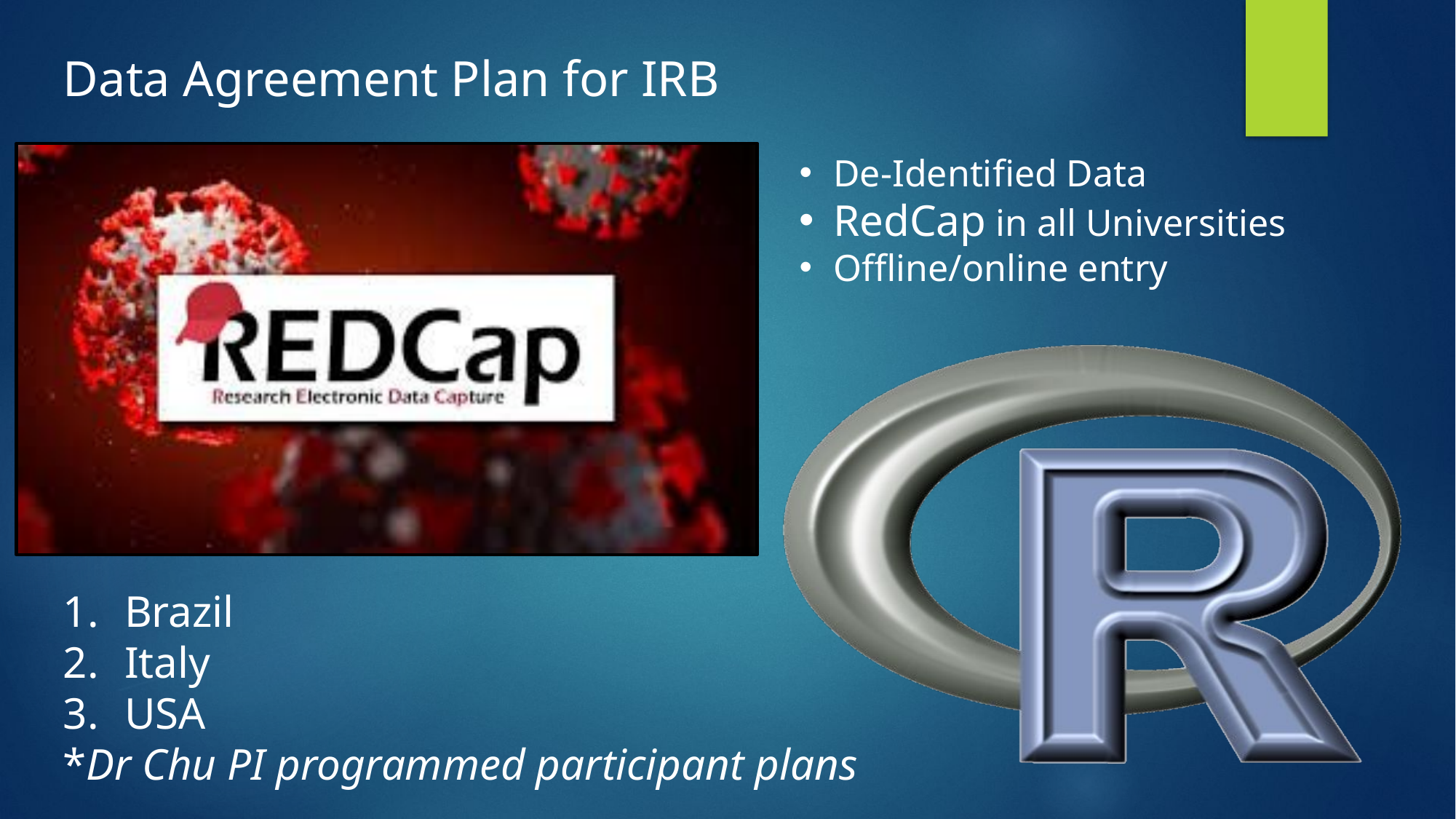

Data Agreement Plan for IRB
De-Identified Data
RedCap in all Universities
Offline/online entry
Brazil
Italy
USA
*Dr Chu PI programmed participant plans

## Slide 4
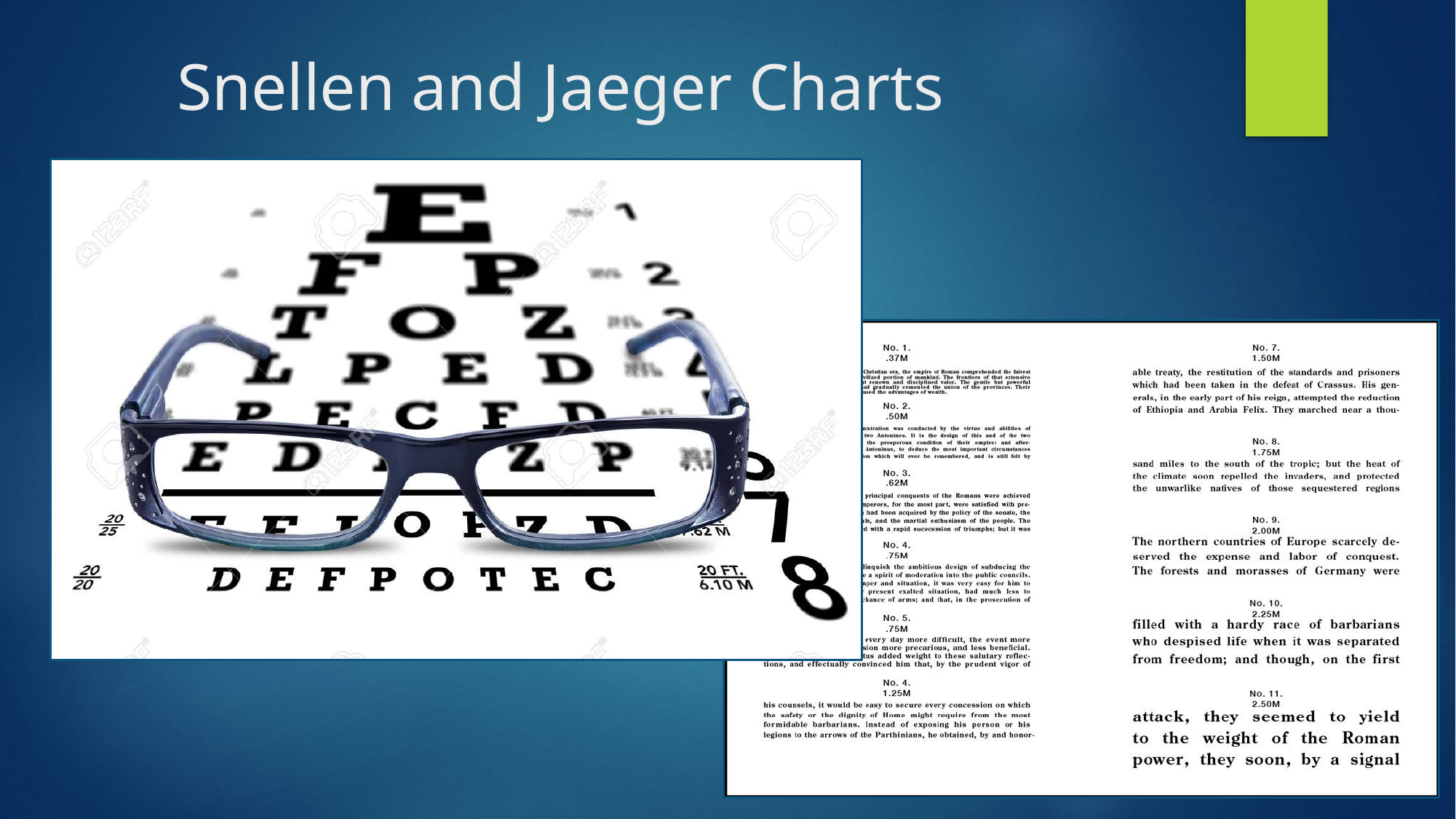

# Snellen and Jaeger Charts

## Slide 5
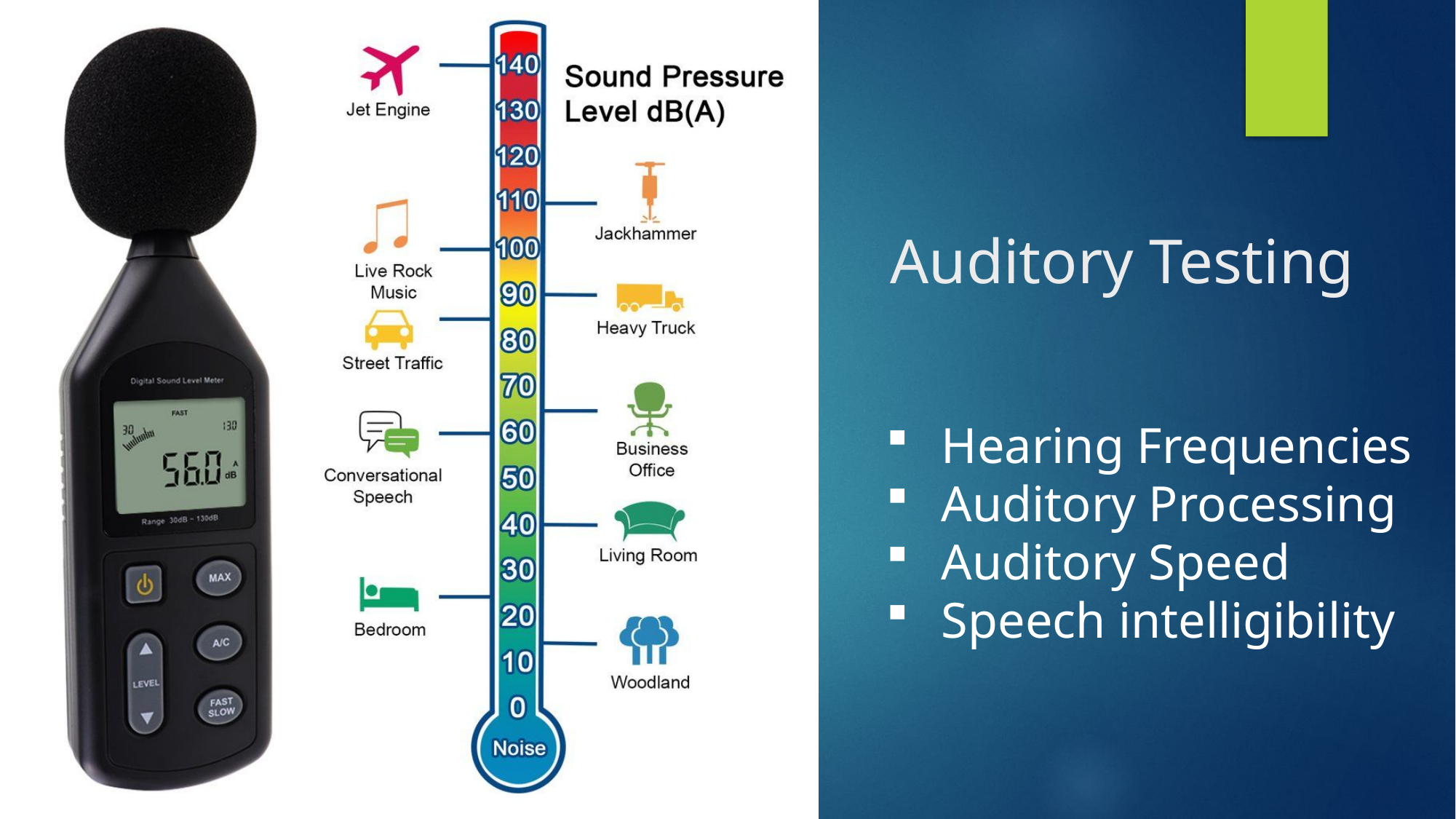

# Auditory Testing
Hearing Frequencies
Auditory Processing
Auditory Speed
Speech intelligibility

## Slide 6
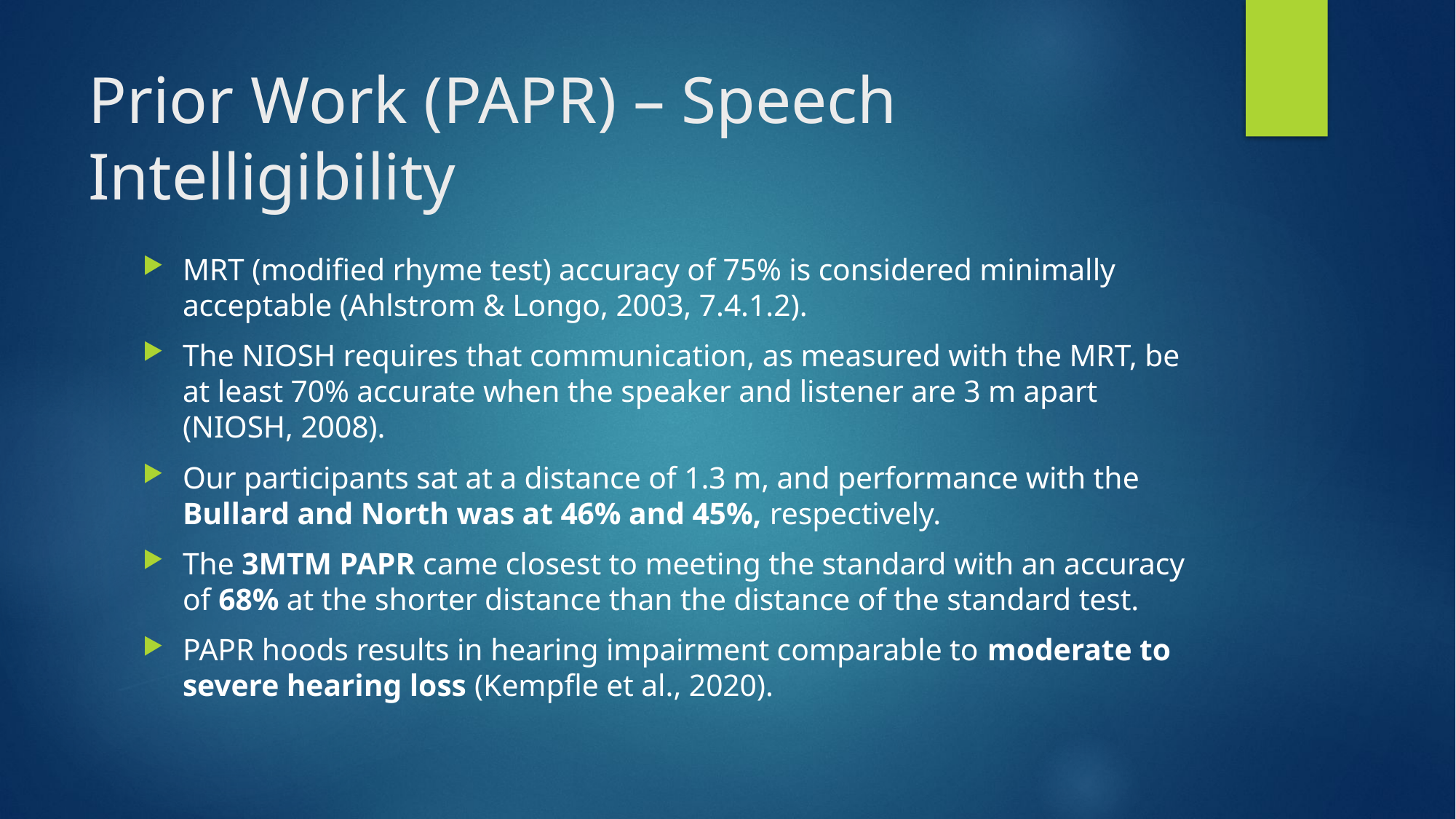

# Prior Work (PAPR) – Speech Intelligibility
MRT (modified rhyme test) accuracy of 75% is considered minimally acceptable (Ahlstrom & Longo, 2003, 7.4.1.2).
The NIOSH requires that communication, as measured with the MRT, be at least 70% accurate when the speaker and listener are 3 m apart (NIOSH, 2008).
Our participants sat at a distance of 1.3 m, and performance with the Bullard and North was at 46% and 45%, respectively.
The 3MTM PAPR came closest to meeting the standard with an accuracy of 68% at the shorter distance than the distance of the standard test.
PAPR hoods results in hearing impairment comparable to moderate to severe hearing loss (Kempfle et al., 2020).

## Slide 7
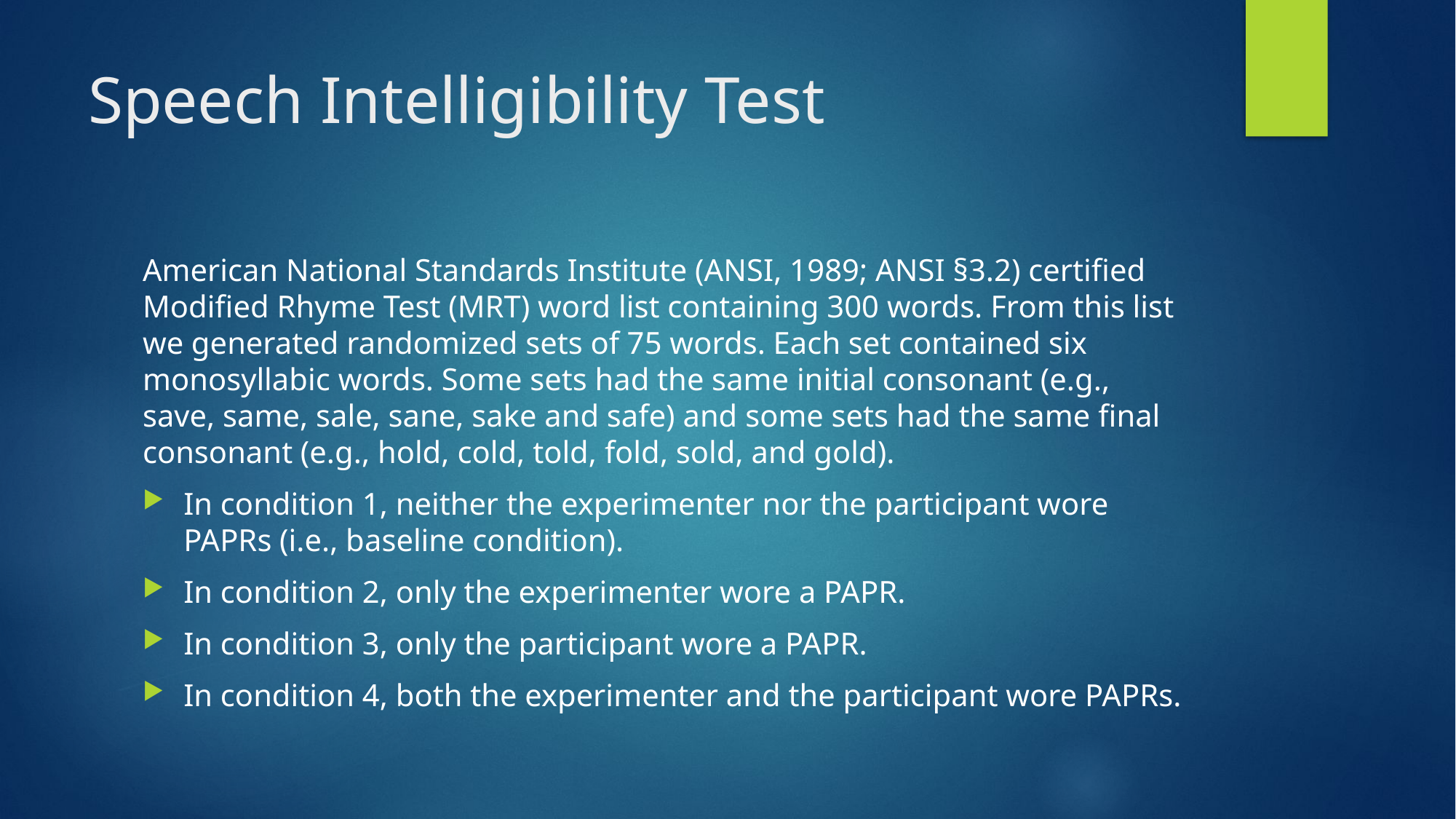

# Speech Intelligibility Test
American National Standards Institute (ANSI, 1989; ANSI §3.2) certified Modified Rhyme Test (MRT) word list containing 300 words. From this list we generated randomized sets of 75 words. Each set contained six monosyllabic words. Some sets had the same initial consonant (e.g., save, same, sale, sane, sake and safe) and some sets had the same final consonant (e.g., hold, cold, told, fold, sold, and gold).
In condition 1, neither the experimenter nor the participant wore PAPRs (i.e., baseline condition).
In condition 2, only the experimenter wore a PAPR.
In condition 3, only the participant wore a PAPR.
In condition 4, both the experimenter and the participant wore PAPRs.

## Slide 8
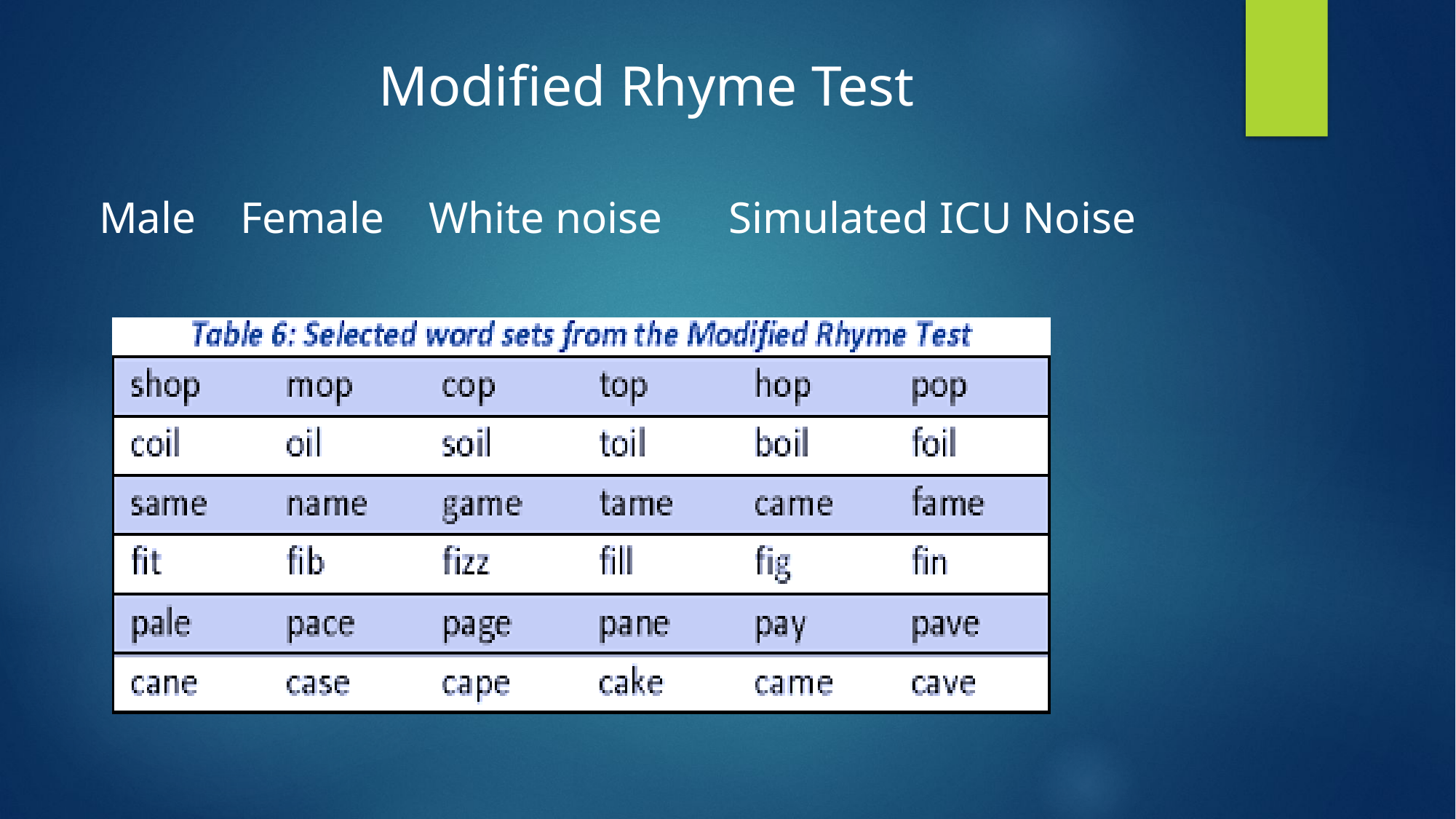

Modified Rhyme Test
Male Female White noise Simulated ICU Noise

## Slide 9
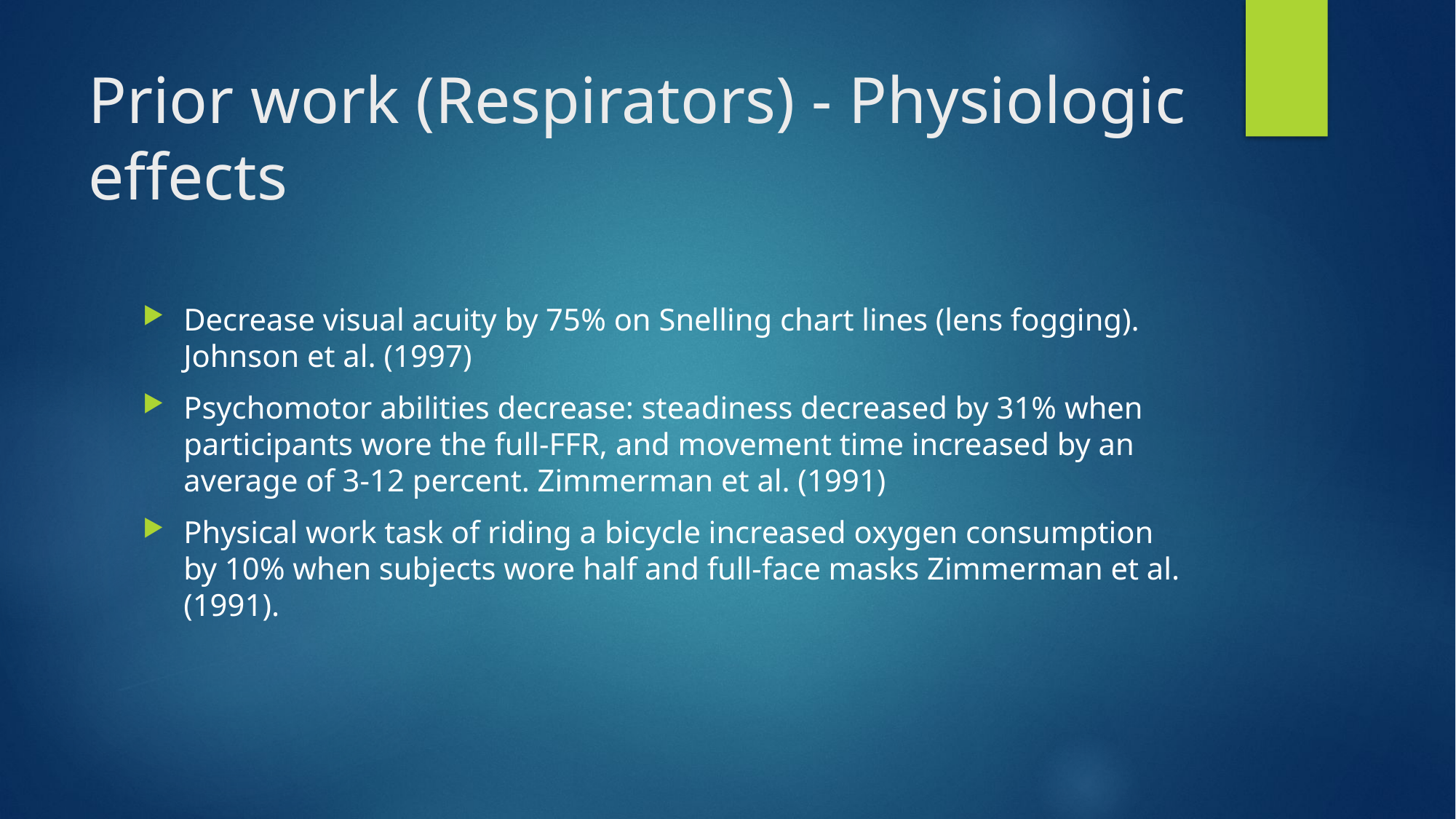

# Prior work (Respirators) - Physiologic effects
Decrease visual acuity by 75% on Snelling chart lines (lens fogging). Johnson et al. (1997)
Psychomotor abilities decrease: steadiness decreased by 31% when participants wore the full-FFR, and movement time increased by an average of 3-12 percent. Zimmerman et al. (1991)
Physical work task of riding a bicycle increased oxygen consumption by 10% when subjects wore half and full-face masks Zimmerman et al. (1991).

## Slide 10
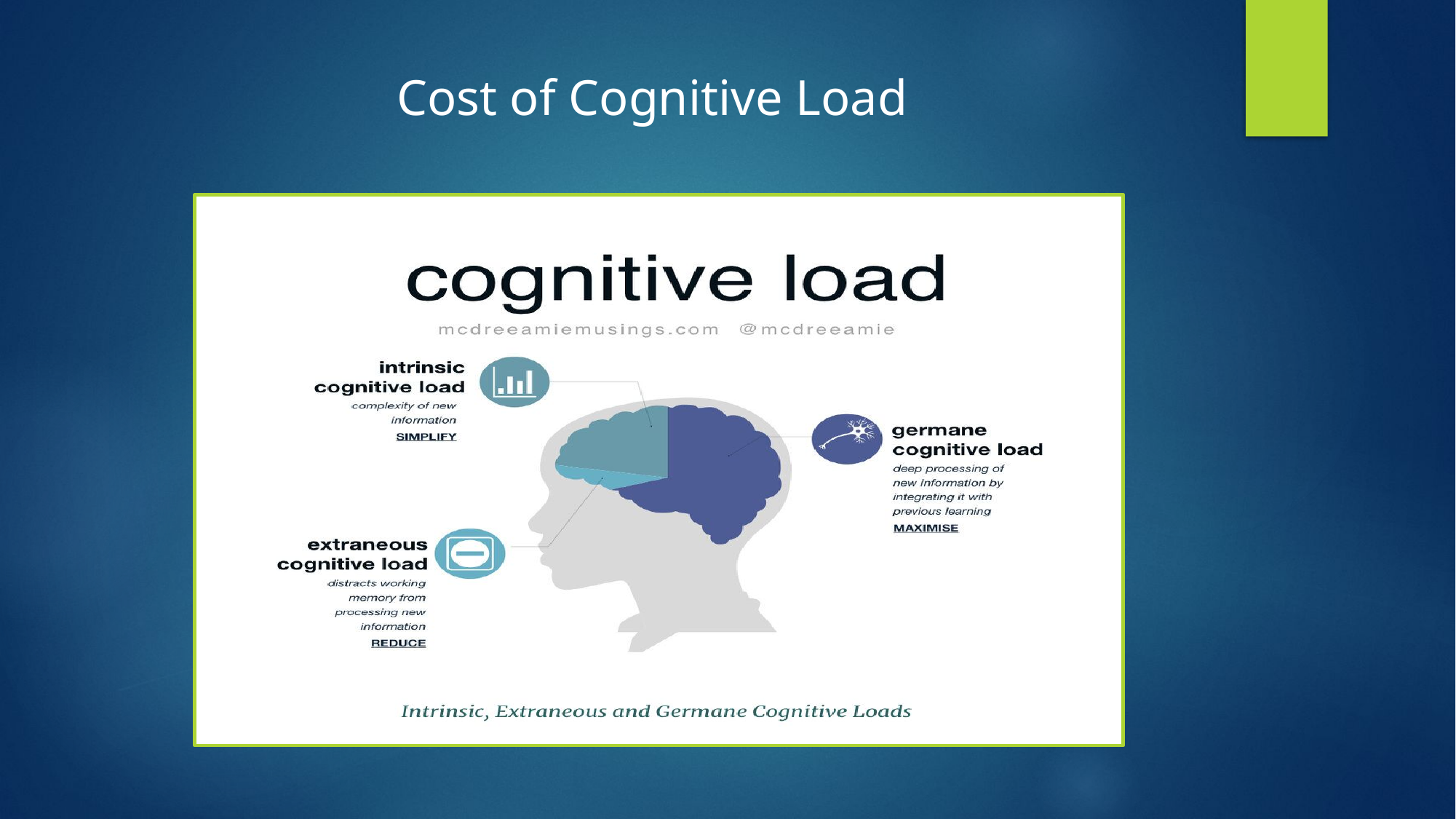

Cost of Cognitive Load

## Slide 11
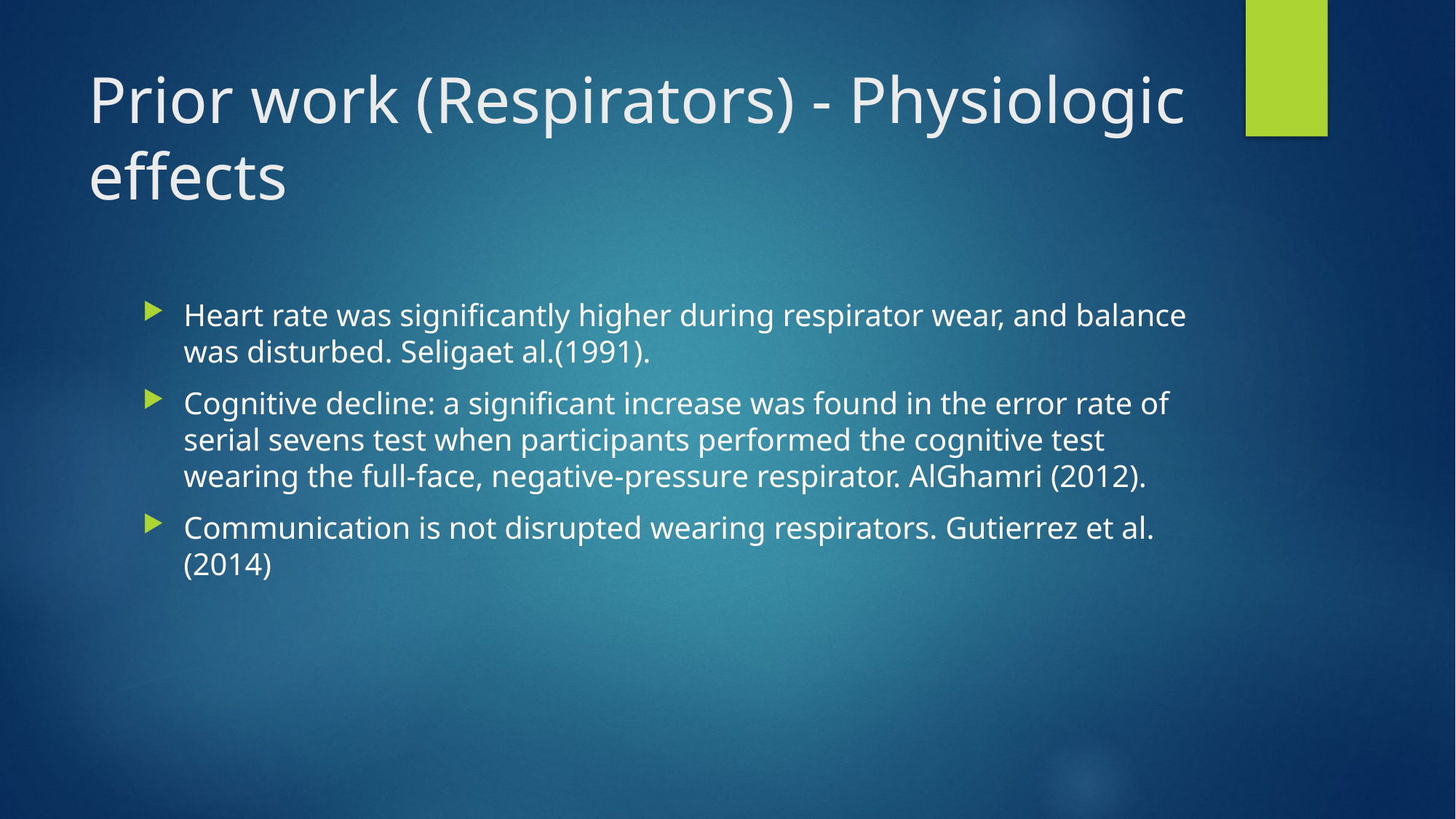

# Prior work (Respirators) - Physiologic effects
Heart rate was significantly higher during respirator wear, and balance was disturbed. Seligaet al.(1991).
Cognitive decline: a significant increase was found in the error rate of serial sevens test when participants performed the cognitive test wearing the full-face, negative-pressure respirator. AlGhamri (2012).
Communication is not disrupted wearing respirators. Gutierrez et al. (2014)

## Slide 12
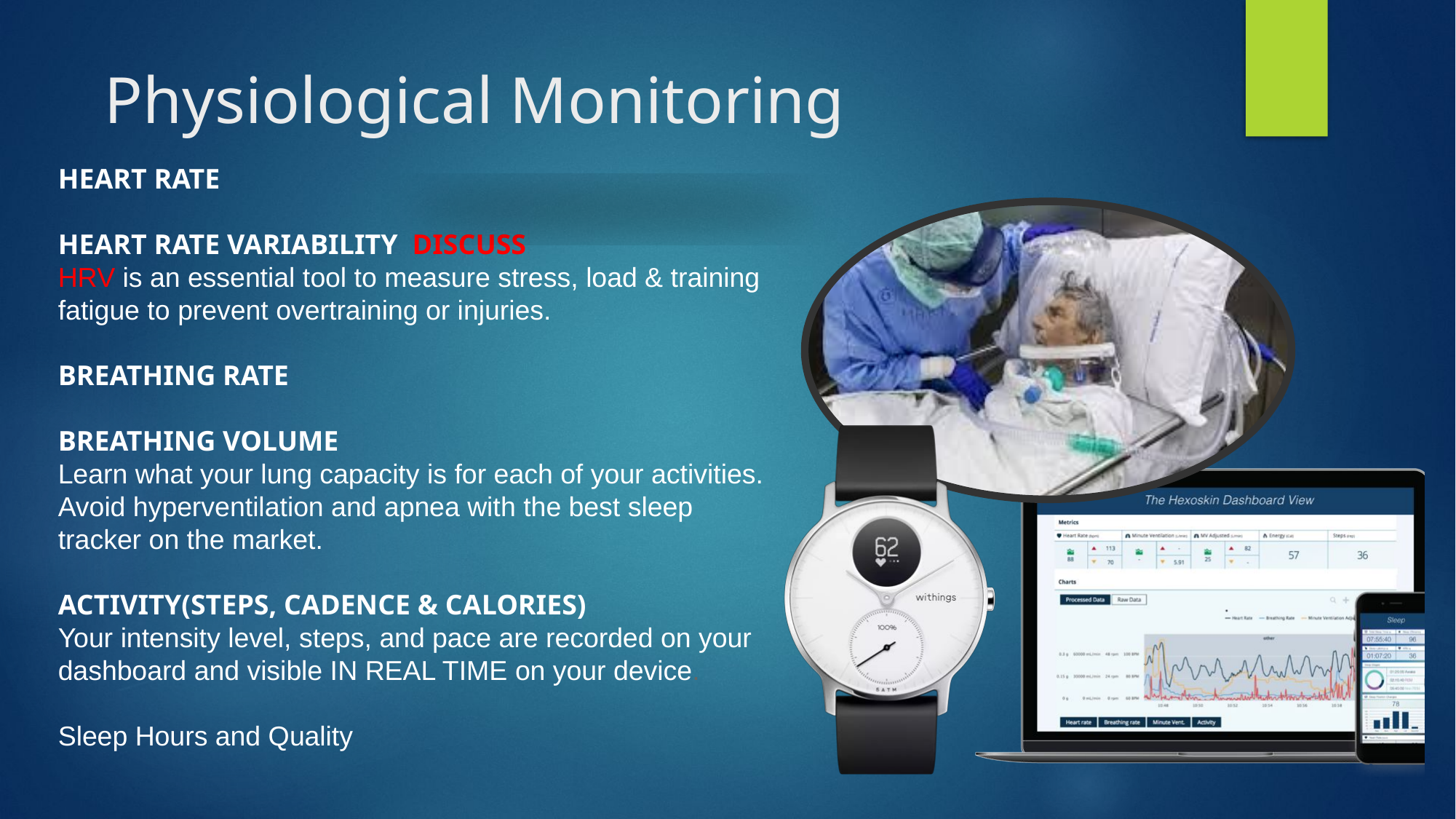

# Physiological Monitoring
HEART RATE
HEART RATE VARIABILITY DISCUSS
HRV is an essential tool to measure stress, load & training fatigue to prevent overtraining or injuries.
BREATHING RATE
BREATHING VOLUME
Learn what your lung capacity is for each of your activities. Avoid hyperventilation and apnea with the best sleep tracker on the market.
ACTIVITY(STEPS, CADENCE & CALORIES)
Your intensity level, steps, and pace are recorded on your dashboard and visible IN REAL TIME on your device.
Sleep Hours and Quality

## Slide 13
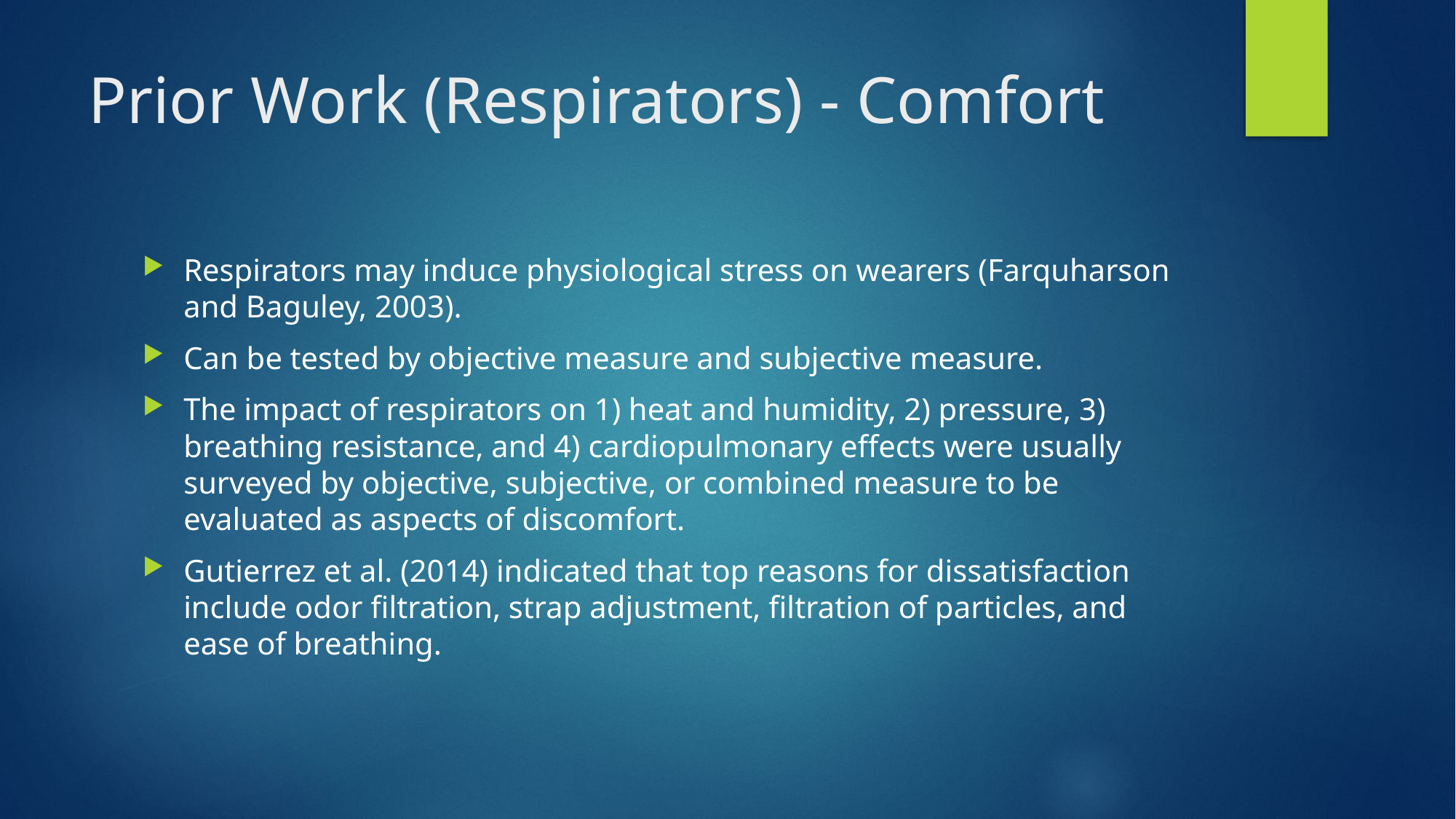

# Prior Work (Respirators) - Comfort
Respirators may induce physiological stress on wearers (Farquharson and Baguley, 2003).
Can be tested by objective measure and subjective measure.
The impact of respirators on 1) heat and humidity, 2) pressure, 3) breathing resistance, and 4) cardiopulmonary effects were usually surveyed by objective, subjective, or combined measure to be evaluated as aspects of discomfort.
Gutierrez et al. (2014) indicated that top reasons for dissatisfaction include odor filtration, strap adjustment, filtration of particles, and ease of breathing.

## Slide 14
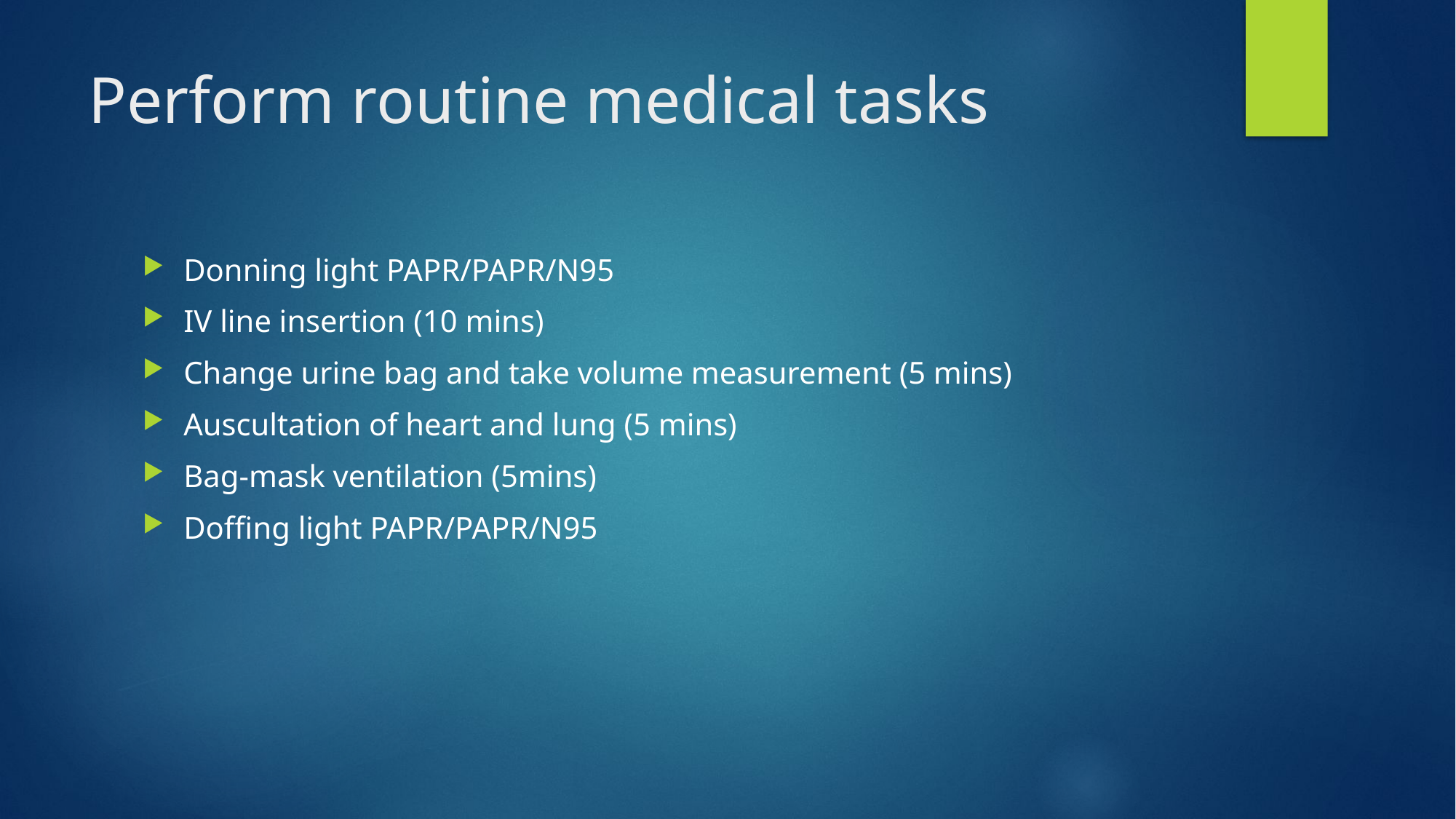

# Perform routine medical tasks
Donning light PAPR/PAPR/N95
IV line insertion (10 mins)
Change urine bag and take volume measurement (5 mins)
Auscultation of heart and lung (5 mins)
Bag-mask ventilation (5mins)
Doffing light PAPR/PAPR/N95

## Slide 15
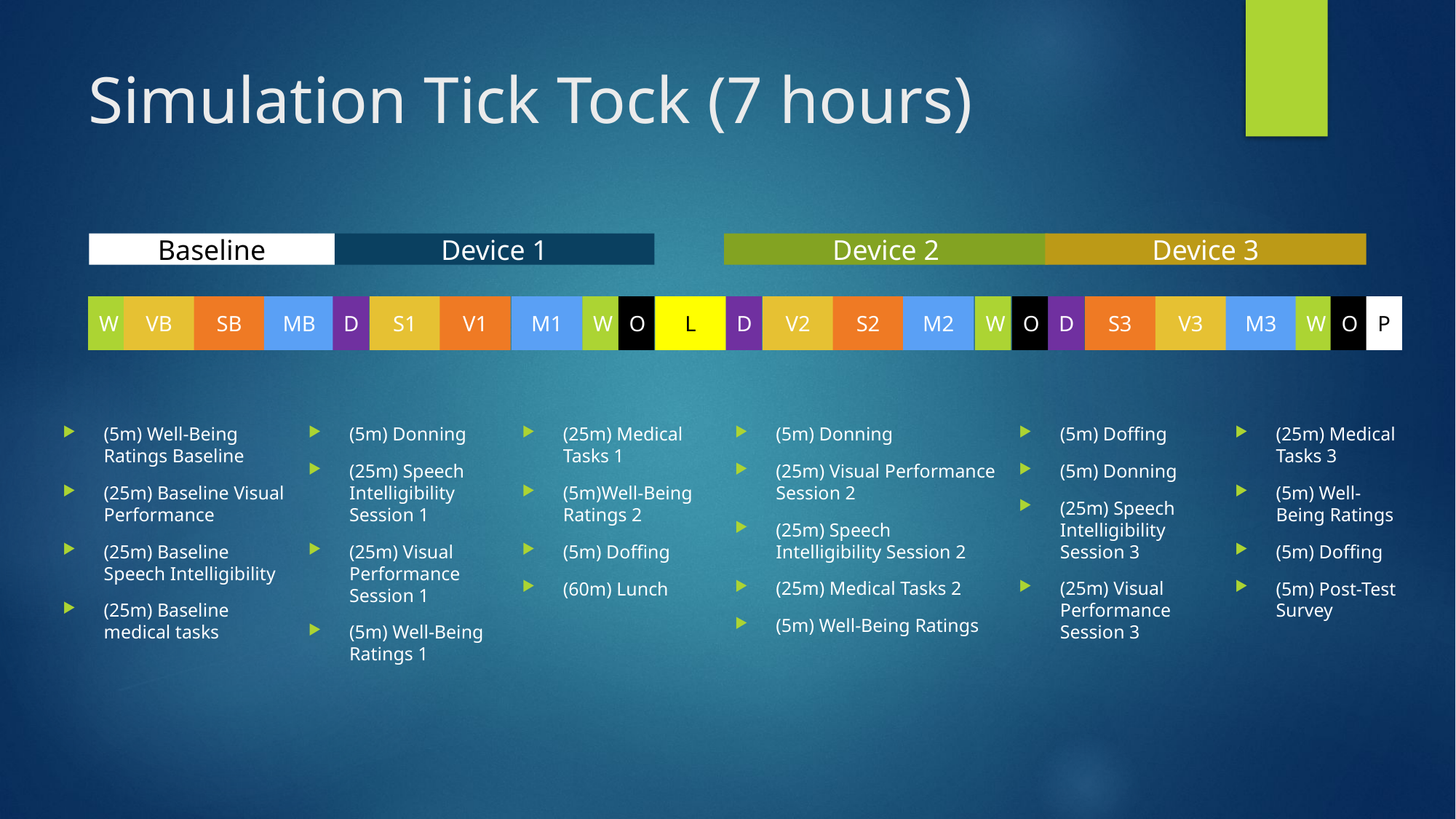

# Simulation Tick Tock (7 hours)
Baseline
Device 1
Device 2
Device 3
W
VB
SB
MB
D
S1
V1
M1
W
O
L
D
V2
S2
M2
W
O
D
S3
V3
M3
W
O
P
(5m) Well-Being Ratings Baseline
(25m) Baseline Visual Performance
(25m) Baseline Speech Intelligibility
(25m) Baseline medical tasks
(5m) Donning
(25m) Speech Intelligibility Session 1
(25m) Visual Performance Session 1
(5m) Well-Being Ratings 1
(25m) Medical Tasks 1
(5m)Well-Being Ratings 2
(5m) Doffing
(60m) Lunch
(5m) Donning
(25m) Visual Performance Session 2
(25m) Speech Intelligibility Session 2
(25m) Medical Tasks 2
(5m) Well-Being Ratings
(5m) Doffing
(5m) Donning
(25m) Speech Intelligibility Session 3
(25m) Visual Performance Session 3
(25m) Medical Tasks 3
(5m) Well-Being Ratings
(5m) Doffing
(5m) Post-Test Survey

## Slide 16
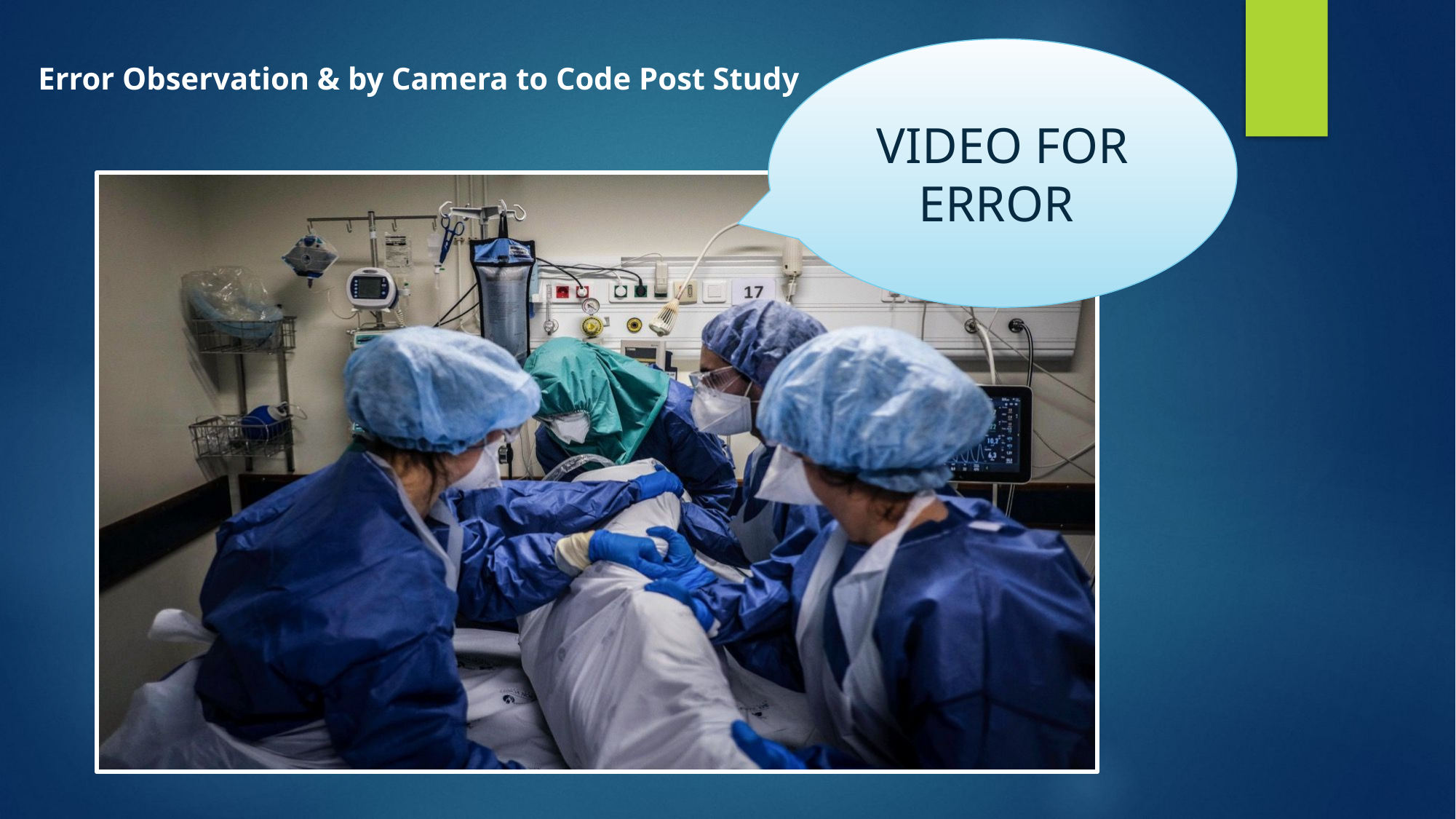

VIDEO FOR ERROR
Error Observation & by Camera to Code Post Study

## Slide 17
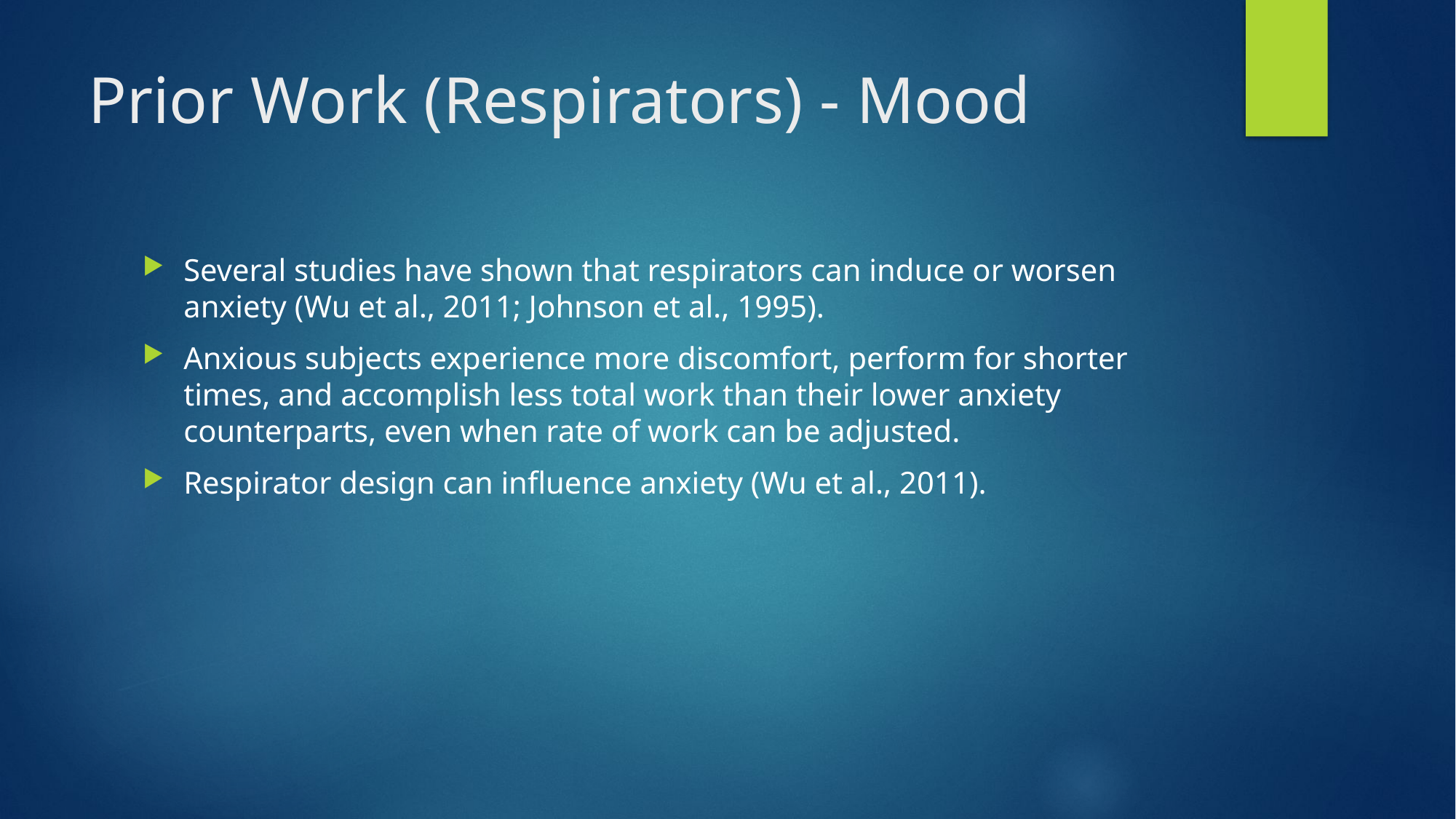

# Prior Work (Respirators) - Mood
Several studies have shown that respirators can induce or worsen anxiety (Wu et al., 2011; Johnson et al., 1995).
Anxious subjects experience more discomfort, perform for shorter times, and accomplish less total work than their lower anxiety counterparts, even when rate of work can be adjusted.
Respirator design can influence anxiety (Wu et al., 2011).

## Slide 18
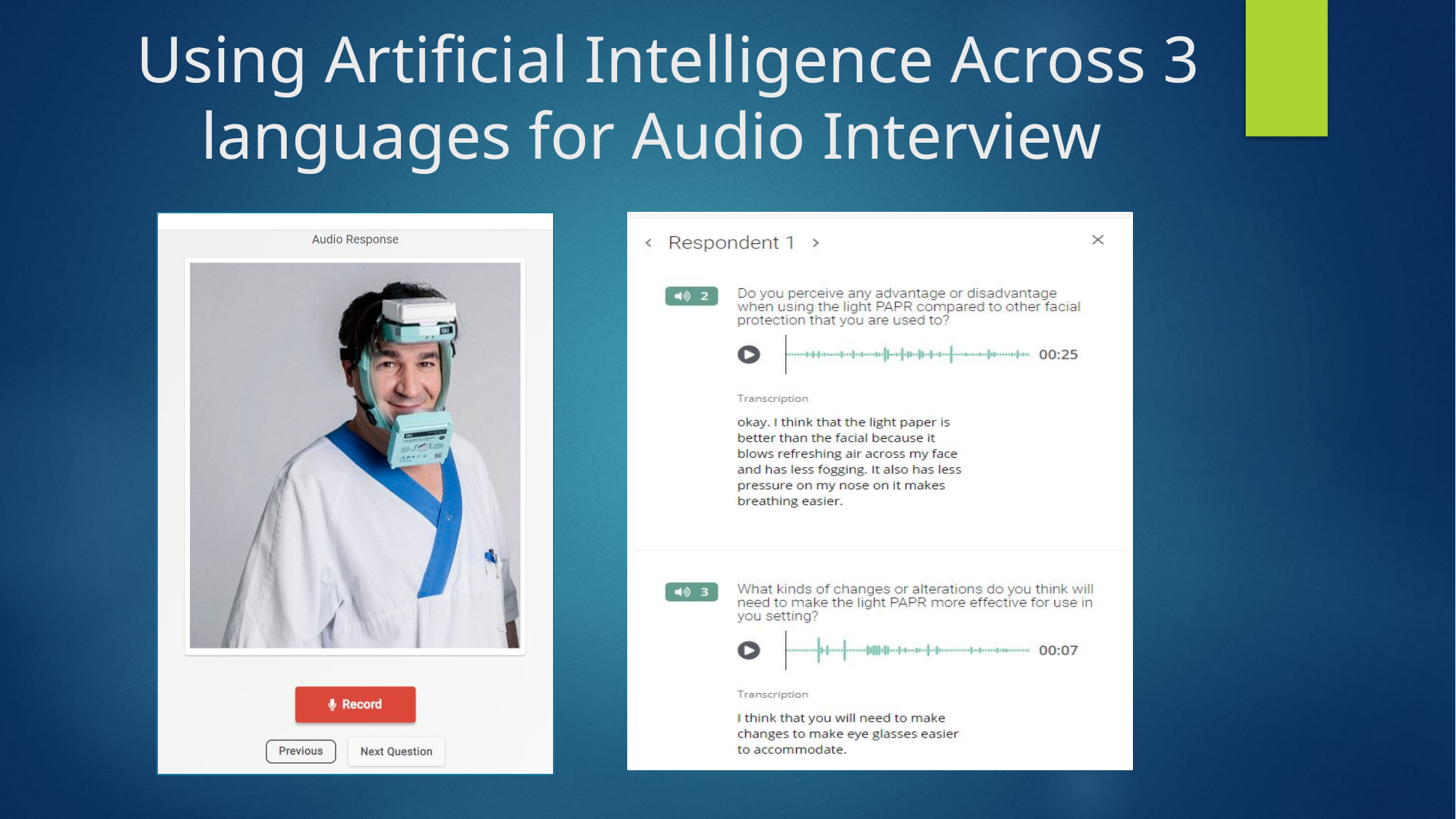

# Using Artificial Intelligence Across 3 languages for Audio Interview

## Slide 19
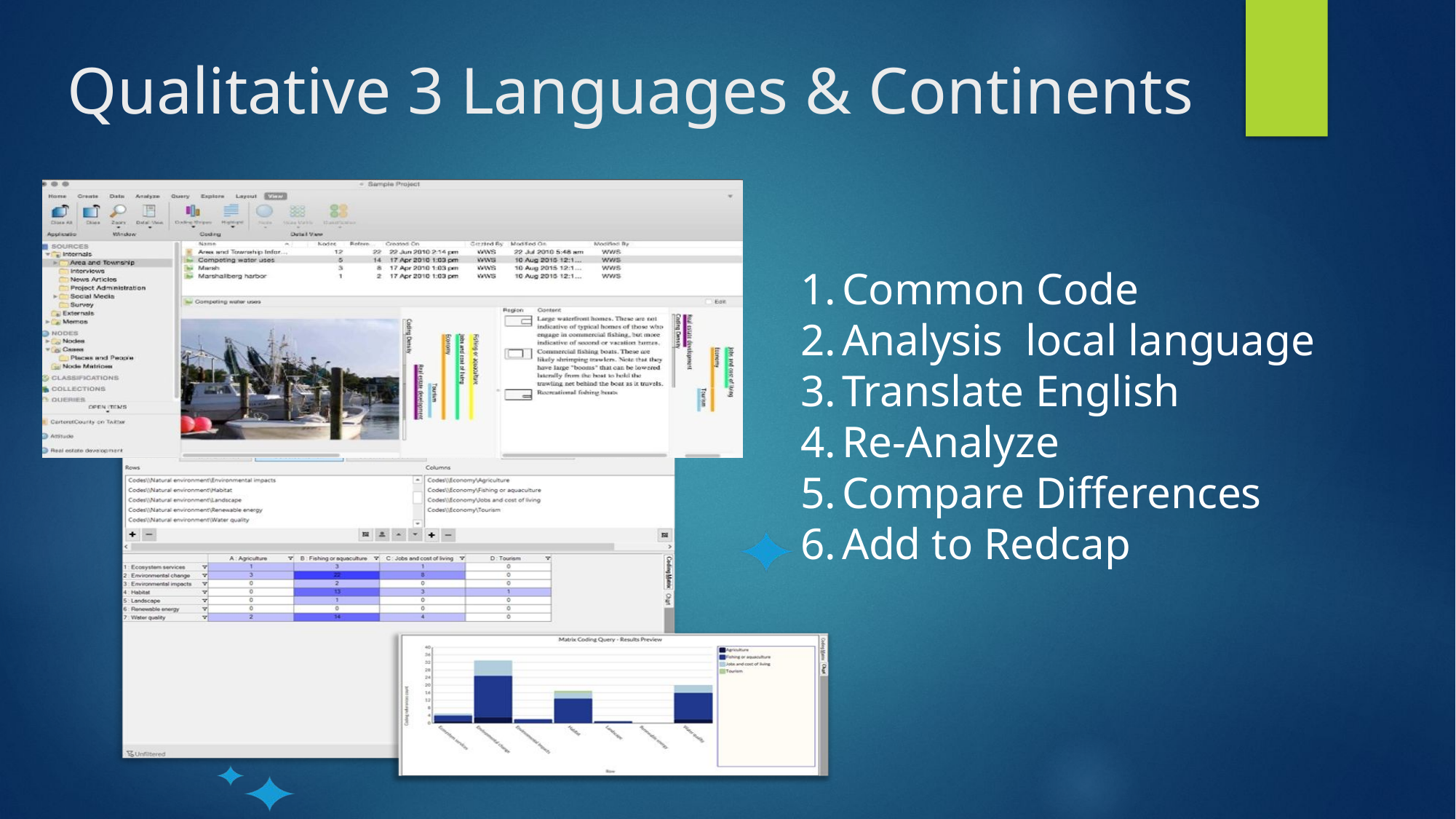

# Qualitative 3 Languages & Continents
Common Code
Analysis local language
Translate English
Re-Analyze
Compare Differences
Add to Redcap

## Slide 20
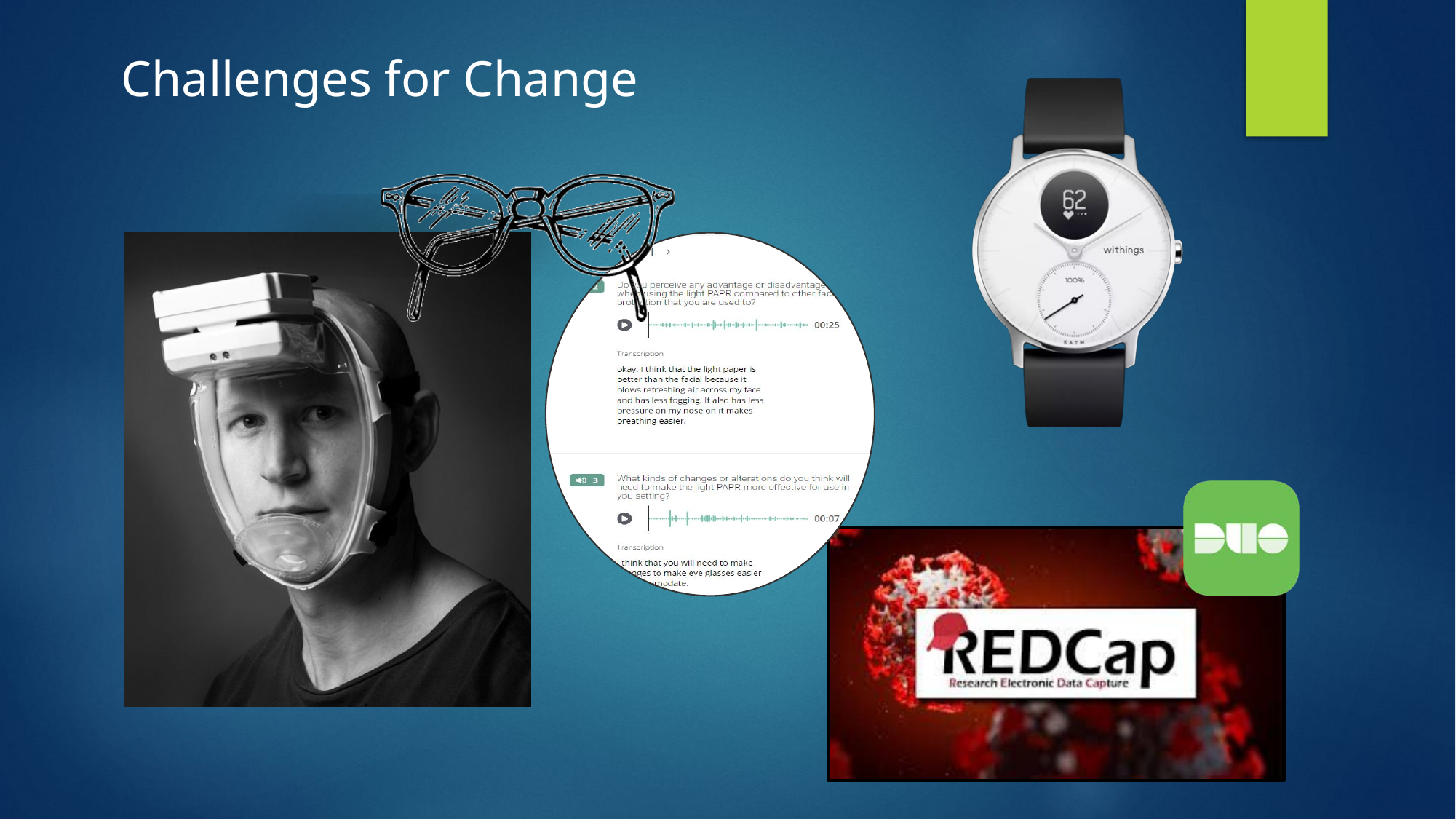

Challenges for Change

## Slide 21
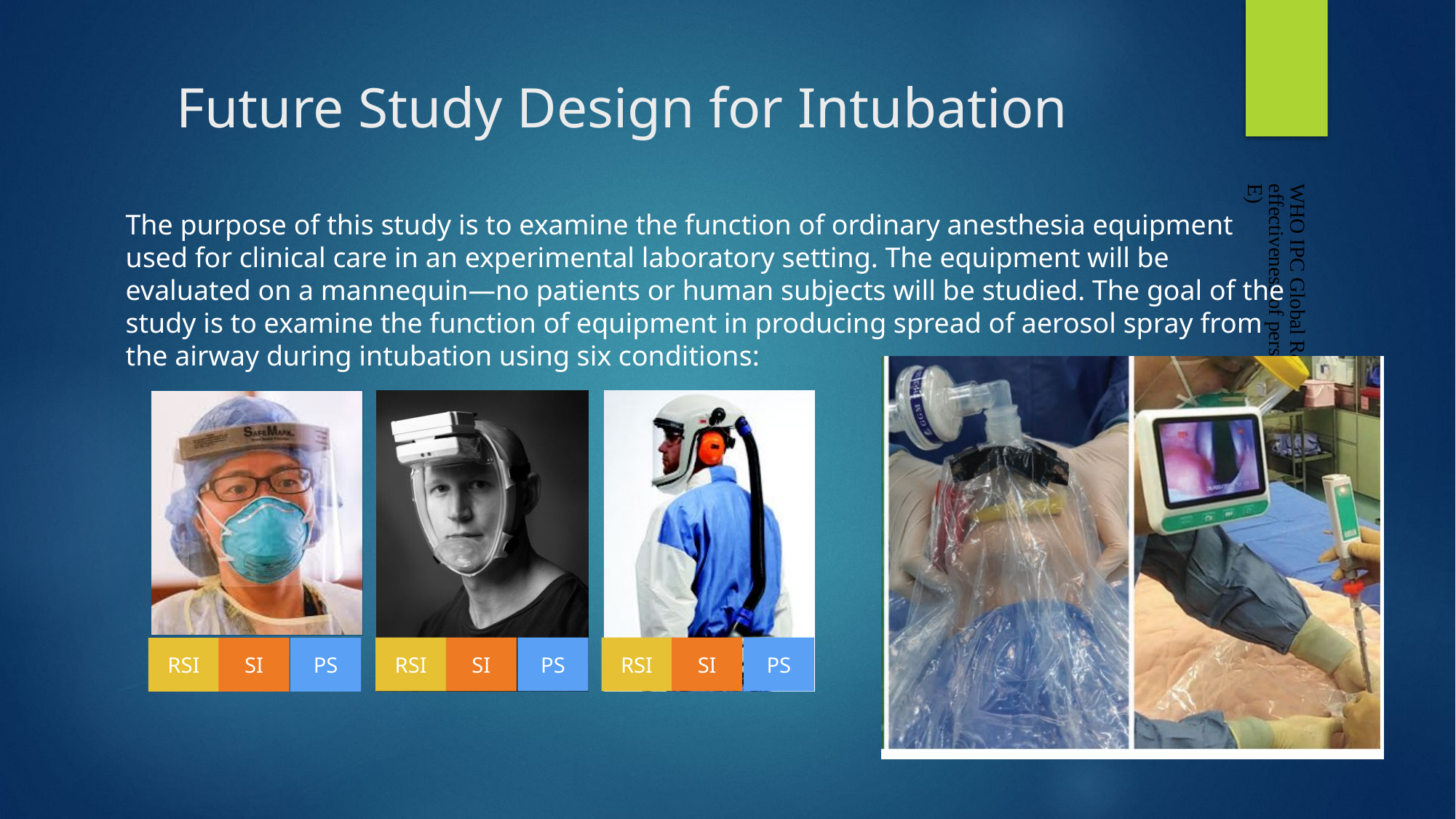

# Future Study Design for Intubation
The purpose of this study is to examine the function of ordinary anesthesia equipment used for clinical care in an experimental laboratory setting. The equipment will be evaluated on a mannequin—no patients or human subjects will be studied. The goal of the study is to examine the function of equipment in producing spread of aerosol spray from the airway during intubation using six conditions:
WHO IPC Global R&D Objective 2: Optimize the effectiveness of personal protective equipment (PPE)
RSI
SI
PS
RSI
SI
PS
RSI
SI
PS

## Slide 22
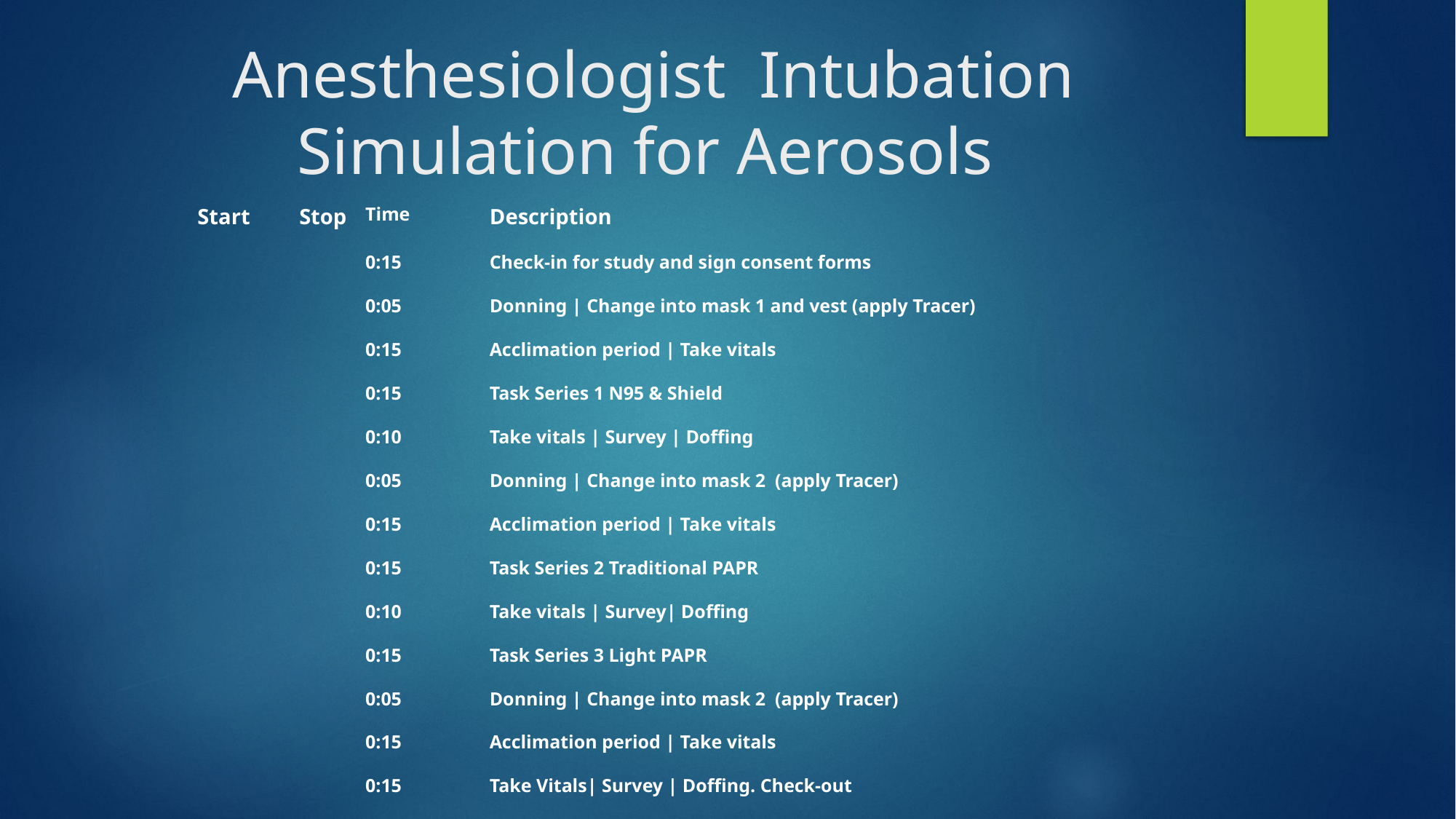

# Anesthesiologist Intubation Simulation for Aerosols
| Start | Stop | Time | Description |
| --- | --- | --- | --- |
| | | 0:15 | Check-in for study and sign consent forms |
| | | 0:05 | Donning | Change into mask 1 and vest (apply Tracer) |
| | | 0:15 | Acclimation period | Take vitals |
| | | 0:15 | Task Series 1 N95 & Shield |
| | | 0:10 | Take vitals | Survey | Doffing |
| | | 0:05 | Donning | Change into mask 2 (apply Tracer) |
| | | 0:15 | Acclimation period | Take vitals |
| | | 0:15 | Task Series 2 Traditional PAPR |
| | | 0:10 | Take vitals | Survey| Doffing |
| | | 0:15 | Task Series 3 Light PAPR |
| | | 0:05 | Donning | Change into mask 2 (apply Tracer) |
| | | 0:15 | Acclimation period | Take vitals |
| | | 0:15 | Take Vitals| Survey | Doffing. Check-out |
| | | 2:05 | Study end (total time) |

## Slide 23
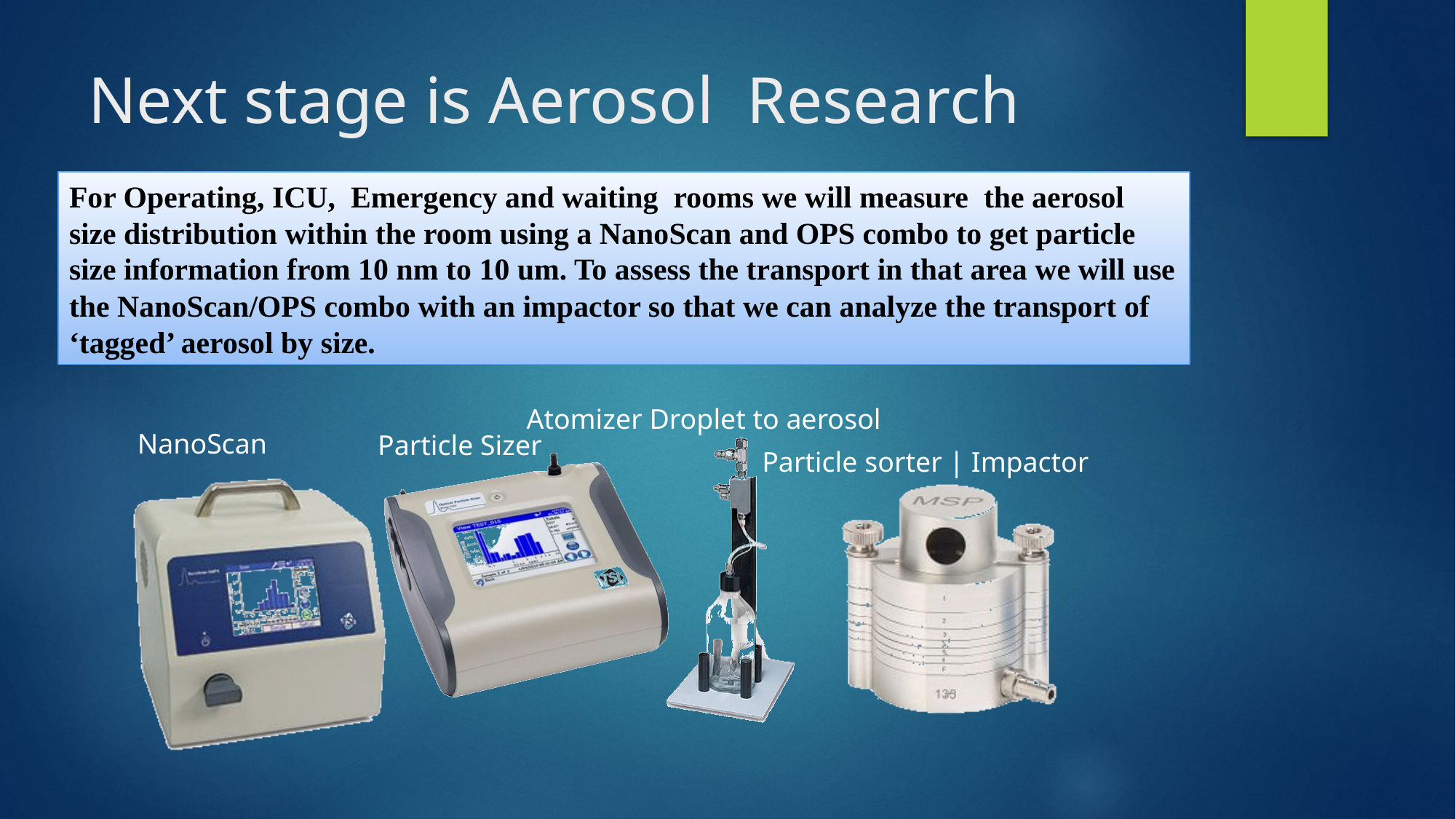

# Next stage is Aerosol Research
For Operating, ICU, Emergency and waiting rooms we will measure the aerosol size distribution within the room using a NanoScan and OPS combo to get particle size information from 10 nm to 10 um. To assess the transport in that area we will use the NanoScan/OPS combo with an impactor so that we can analyze the transport of ‘tagged’ aerosol by size.
Atomizer Droplet to aerosol
NanoScan
Particle Sizer
Particle sorter | Impactor

## Slide 24
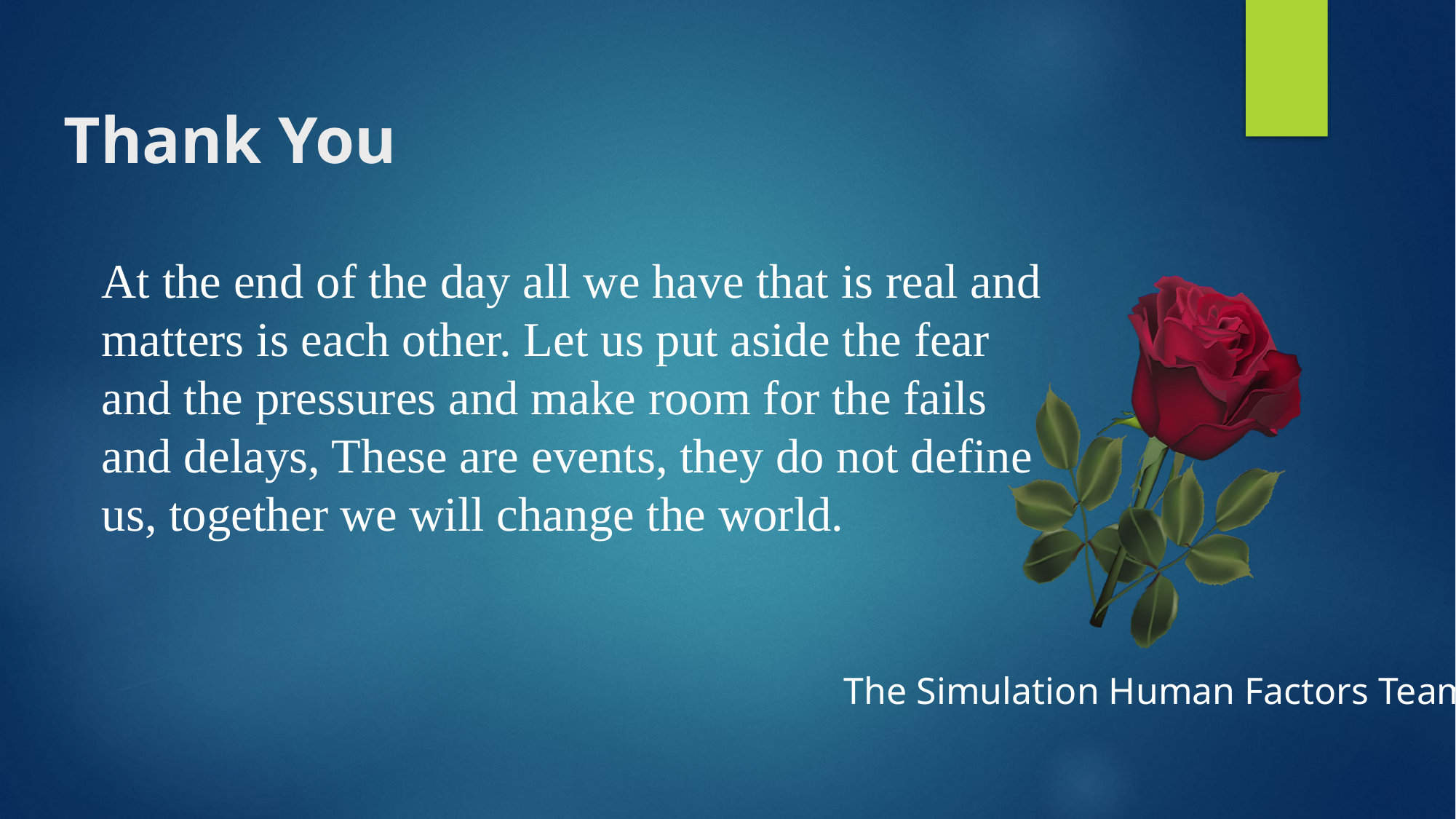

# Thank You
At the end of the day all we have that is real and matters is each other. Let us put aside the fear and the pressures and make room for the fails and delays, These are events, they do not define us, together we will change the world.
The Simulation Human Factors Team
